# VicPred: A *Vibrio cholerae* Genotype Prediction Tool

**DOI:** 10.3389/fmicb.2021.691895

**Published:** 2021-09-09

**Authors:** Imchang Lee, Sung-Min Ha, Min-gyung Baek, Dong Wook Kim, Hana Yi, Jongsik Chun

**Affiliations:** ^1^School of Biological Sciences, Seoul National University, Seoul, South Korea; ^2^Institute for Biomaterials, Korea University, Seoul, South Korea; ^3^Interdisciplinary Program in Precision Public Health, Korea University, Seoul, South Korea; ^4^Department of Public Health Sciences, Korea University, Seoul, South Korea; ^5^Department of Pharmacy, College of Pharmacy, Institute of Pharmacological Research, Hanyang University, Ansan, South Korea; ^6^School of Biosystems and Biomedical Sciences, Korea University, Seoul, South Korea

**Keywords:** cholera, *Vibrio cholera*, 7th pandemics, O serogroup, CTXφ, SXT, VPI, VSP

## Abstract

Genomic information can be used to predict major pathogenic traits of pathogens without the need for laboratory experimentation. However, no *Vibrio cholerae* genome-based trait identification tools currently exist. The aim of this study was to develop a web-based prediction tool to identify *Vibrio* pathogenic traits using publicly available 796 whole-genome sequences of *V. cholerae*. Using this application, 68 structural O-antigen gene clusters belonging to 49 serogroups of *V. cholerae* were classified, and the composition of the genes within the O-antigen cluster of each serogroup was identified. The arrangement and location of the CTX prophage and related elements of the seventh cholera pandemic strains were also revealed. With the versatile tool, named VicPred, we analyzed the assemblage of various SXTs (sulfamethoxazole/trimethoprim resistance element) and major genomic islands (GIs) of *V. cholerae*, and the increasing trend in drug-resistance revealing high resistance of the *V. cholerae* strains to certain antibiotics. The pathogenic traits of newly sequenced *V. cholerae* strains could be analyzed based on these characteristics. The accumulation of further genome data will expedite the establishment of a more precise genome-based pathogenic traits analysis tool.

## Introduction

The seventh pandemic of *Vibrio cholerae* is distinct from previous pandemics owing to the emergence of new serotypes and biotypes (*V. cholerae* O1 biovar. El Tor and *V. cholerae* O139), which spread rapidly over a wider area and cause new disease patterns with relatively moderate symptoms lasting longer. These strains, the causative agents of the seventh pandemic, had different traits than the former strain that caused the last pandemic. In fact, those strains have been found to have a different serogroup (e.g., *V. cholerae* O139) from the previous pathogenic *V. cholerae* (*V. cholerae* O1) or a new type of toxin (e.g., cholera toxin prophage CTX-El Tor) (Ramamurthy et al., 1993). Researches on new mutant strains have shown that the integration of cholera toxin-related phages is dynamic and variable in combination (Ramamurthy et al., 1993; Bik et al., 1996; Dalsgaard et al., 2001; Lee et al., 2009; Kim et al., 2014b, 2015). Although these pioneering studies have broadened our knowledge and understanding of cholera, the number of O serogroups to be analyzed increases, and more investigations are needed to determine the exact type of cholera toxin (Das et al., 2010). Considering the continuous variation in *V. cholerae* that appeared in the recent seventh pandemic times, the emergence of a strain with novel phenotypic characteristics is expected. Therefore, the accumulation of knowledge about cholera based on the understanding of *V. cholerae* strains will play a crucial role in the accurate research of new cholera that may appear in the future.

The O-antigen of *V. cholerae* is the major protective epitope that allows pathogens to evade pre-existing immunity to cholera. The O-antigen gene cluster (OAGC) is composed of various genes, including biosynthesis-related genes, various transferases, and several transposons or mobile elements (Reeves et al., 1996). O-antigens are known to have more than 200 phenotypes due to various combinations of these genes. For O1, which is the representative serogroup of *V. cholerae*, an estimated 19 genes that generate the O1-specific antigen are distributed between the epimerase gene, *gmhD*, and cleavage gene, *rjg*, which are involved in the major stages of lipopolysaccharide (LPS)-core generation (Chatterjee and Chaudhuri, 2004). Among them, the 13th–15th open reading frame (ORF) gene, *wbeT* (previously called *rfbT*), is a methyltransferase gene known to determine the O1 serotypes Ogawa, Inaba, and Hikojima, which are serotypes of O1 (Stroeher et al., 1992). Although the structures of OAGC for O1, a major pandemic serogroup, and O139 that newly appeared in the seventh pandemic have been relatively well studied, to date, studies on the OAGC structure of most of the other serogroups have not been sufficient.

The cholera enterotoxin genes (*ctxAB*) are part of a lysogenic CTX genome, which is composed of nine genes integrated into *V. cholerae* chromosomes. *ctxAB* encodes the A and B subunits of CT, while the other genes are involved in the lysogenic conversion of CTXφ. Two genetic elements, RS1 and TLC (toxin-linked cryptic), are also important external genetic elements related to the replication and integration of the CTX phage, respectively. The RS1 element is approximately 2.6 kb (*rstRABC*), which is known to be carried by the satellite phage RS1φ. The *rstC* is a unique gene that encodes an anti-repressor that promotes CTX gene expression. TLC elements refer to the five genes (TLC1, TLC2, TLC3, TLC4, and TLC5) that are carried by a satellite phage, TLC, and are known to facilitate the integration of CTXφ and RS1φ by causing structural changes at the *dif* site of *V. cholerae* (Hassan et al., 2010). CTX phages could be integrated into either or both chromosomes, and some *V. cholerae* strains contain multiple copies of TLC, CTX prophage, and RS1 element (Hassan et al., 2010).

Recently, the antibiotic resistance of *V. cholerae* has been reported to be steadily increasing. In a study of cholera in Bangladesh in 1979, 16.7% of isolates were reported to be resistant to antibiotics, and more than 10% of them were reported to have multiple drug resistance (Glass et al., 1980). Subsequent cholera studies in 1991 reported that 70% of cholera strains have multiple antibiotic resistance, making antibiotic resistance a major issue (Siddique et al., 1992). In addition, a study in East Africa in the mid-1990s found that all *V. cholerae* strains isolated in Tanzania and Rwanda were resistant to tetracycline, which was often prescribed for cholera (Materu et al., 1997). Drug-resistance studies on co-trimoxazole in Somalia and Kenya reported only 15% resistance in 1994, but 90% in 1996, indicating that the antibiotic resistance of the cholera bacteria is markedly increasing (Materu et al., 1997). Accordingly, studies conducted between the 1970s and the 1990s have shown that antibiotic resistance in *V. cholerae*, although partial and regional, has been increasing in various ways.

Although chromosome I of *V. cholerae* has a relatively low mutation rate compared to that of other species (Sung et al., 2016), the mobile genetic element-driven pathogenicity islands contribute to rapid evolutionary changes in the virulence of cholera pathogens. *V. cholerae* strains harbor several genomic islands that contribute to the mobility of genes and other metabolic functions (Mutreja et al., 2011). Most of the 7th pandemic *V. cholerae* strains harbor four important mobile genetic elements, including VPI-1,2 (*Vibrio* pathogenic Island-1,2) and VSP-1,2 (*Vibrio* 7th pandemic island-1,2). As a result, non-endogenous, horizontally acquired genomic islands (GI) and pathogenic *V. cholerae* strains have crucial pathogenic features, including sporadic distribution, DNA recombination in the region of *dif* loci, variation in GC content, and genomic instability (Kumar et al., 2020). Consequently, in addition to the general features, including O serogroups, typing of CTXφ (and related genetic elements), and drug resistance, more information about GIs is needed to analyze the pathogenicity of *V. cholerae*.

Currently, more than one thousands *V. cholerae* raw assemblies, including the classical type and El Tor type, exist in the NCBI assembly database. Therefore, the demand for a data-based integrated research platform that can analyze *V. cholerae* is increasing. In this context, we developed a web-based genotype prediction tool to identify *Vibrio* pathogenic traits. To demonstrate the performance of this web-based prediction tool, 796 *V. cholerae* genome sequences collected from a public database were analyzed for their major features, including O-antigen types, CTXφ, antibiotic resistance, and GIs. The web-based prediction tool, VicPred (*Vibrio cholerae* genotype prediction tool), is freely available at.^1^

## Materials and Methods

### Data Collection

All *V. cholerae* genome data were retrieved from the EzBioCloud (Yoon et al., 2017). Although there are roughly 1,000 or more *V. cholerae* genomes available in public databases, we used the EzBioCloud database to collect high-quality genome data only. According to the EzBioCloud pipeline, genomes from public databases were collected and curated using a sophisticated filtering pipeline for the validated data (Yoon et al., 2017). The genomes of duplicate strains were excluded, and assemblies suspected of contamination were excluded using the CheckM (Parks et al., 2015) and ContEst16S (Lee et al., 2017) programs in the filtering pipeline of EzBioCloud. A total of 796 *V. cholerae* genome data were used in this study (Supplementary Table 1). All programs were developed using the JAVA and JAVASCRIPT programming languages.

### O-Antigen Prediction (*V. cholerae* O-Antigen Serotyping Program)

#### Extraction of Full OAGC

The genes, *gmhD* (locus tag = VC_0240 of NC_002505.1, protein id = WP_000587795.1, synonyms: *rfaD*, *hldD*, *waaD*, *nbsB*, *htmM*, *ECK3609*, *b3619*, and *JW3594*) of *V. cholerae* N16961 and *ysh1* (locus tag = VC_0264 of NC_002505.1, protein id = WP_000454280.1), known as the right junction gene (*rjg*) of the O-antigen gene cluster, were used at the start and end of the OAGC, respectively (Bik et al., 1996; Sozhamannan et al., 1999). To find the beginning and end of the OAGC, the USEARCH (v8.0.1517) program (Edgar, 2010) was used with the following threshold: identity > 90% and query coverage > 0.9. The 90% identity cutoff was selected as the minimum nucleotide identity by comparing all the *gmhD* and *rjg* genes in all *V. cholerae* genomes.

#### CDS Assignment of OAGC

To predict the coding sequence (CDS) within OAGC, a prodigal 2.6.1 (Hyatt et al., 2010) using dynamic programming of prokaryotic gene finding algorithm was used. The UniProt (Apweiler et al., 2004), KEGG (Kanehisa and Goto, 2000), and NCBI NR databases (Pruitt et al., 2005) were used to assign the extracted CDS. When the CDS name was different in each database, the CDS name was matched to the bacterial polysaccharide gene nomenclature (BPGN) protocol (Reeves et al., 1996). If the CDS could not be assigned a name, the CDS was named a hypothetical protein.

#### Selection of the OAGC Standard Gene Set

All the CDSs of OAGCs extracted from all genomes were clustered based on a minimum nucleotide identity of 90% using the USEARCH program. The representative sequence of each group was selected using the following criteria: (1) the sequence that occupies the most frequency in each cluster, (2) the length of the sequence should be within the top 10%, (3) exclusion of sequences containing ambiguous nucleotide sequences (N), and (4) exclusion of sequences with missing information.

#### Construction of a Representative Binary Array Set

The *V. cholerae* O-antigen Serotyping program (V.O.S.) uses the standard gene set representing each CDS cluster, a present/absent (1/0) list of arrays for the OAGCs of all the genomes, indicating whether each gene exists in the OAGCs. For all genomes, the criterion of the USEARCH identity (> 0.9) was applied to create a binary array containing 1 (gene presence) and 0 (absence) in the order of the standard gene set. After the Jaccard index (JI) comparison of the binary arrays between genomes, the genomes with the same values (*JI* = 1.0) were grouped. The genome with the highest genome quality or of the most studied strain was used as the representative genome for each group. The binary arrays of representative genomes were collected for use as representative binary array sets.

#### Decision of the Serogroups

**FIGURE 1:**
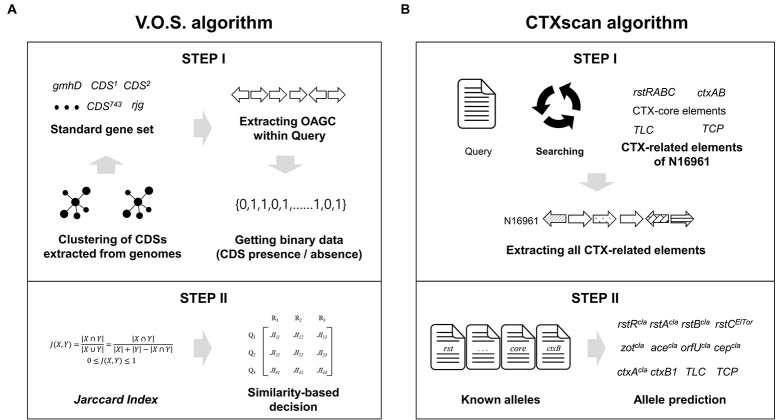
Schematic diagram of the V.O.S. and CTXscan algorithms. **(A)** All the CDS of the OAGC were extracted from the *V. cholerae* genome dataset, and clustering was performed to create a standard gene set. To create a representative binary vector containing information on whether CDS is present or absent, the USEARCH program was used. Finally, the serogroup type was determined by comparing JI between a binary vector of the query and representative. **(B)** To extract the CTX-related elements from the query genome, the known prophage gene contents of N16961 were used. The extracted elements were compared against known alleles previously reported in other studies. The phage type prediction was performed based on sequence similarity.

As a first step, two representative genes, *gmhD* and *rjg*, were identified with 90% identity using the USEARCH program. If both genes exist and are in the same contig, the V.O.S. extracted the site between two genes by judging whether they had a full OAGC. Next, using the standard gene set, the extracted OAGC was examined to determine whether each standard gene exists based on the following cutoff: identity > 90%, length coverage > 90%, *e*-value < 1e—5, and bit score > 500. The V.O.S. creates a query binary array by determining the presence or absence of each standard gene. Finally, the array was compared to the representative binary array set, and the serogroup was determined to be that of a representative genome having the closest (closer to 1) value with the query (Figure 1A).

#### Serotypes Within the O1 Serogroup

To derive reference genes for the prediction of serotypes within the O1 serogroup, the *wbeT* (synonym: *rfbT*) gene, which is known to define the characteristics of the O1 serotype (Ogawa and Inaba) (Colwell et al., 1995), was extracted from all experimentally defined O1 type genomes. However, the Hikojima type was excluded from the analysis because no available genome was found. For identification, 99% id, 95% length coverage, and an *e*-value < 1e—10 were used as the cutoff values.

### Prediction of the CTX Prophage and Related Elements (CTXscan)

#### Selection of the Standard Gene Set

The prediction of TLC, CTX, and RS1 is comprised of two steps: extracting a standard gene set and predicting alternative alleles of the genes. The standard gene sets of the TLC, RS1, and CTX prophage elements were reconstructed according to previous studies (Lee et al., 2009; Kim et al., 2014a,b, 2015). The standard gene sets consisting of 15 genes, including *cep*, *orfU*, *ace*, *zot*, *ctxA*, *ctxB*, *rstA*, *rstB*, *rstC*, *rstR* (*rstR*^*cla*^, *rstR*^*El*^ ^*Tor*^, and *rstR*^*CTX*–*O*139^), and TLC (*VC1466* (TLC1), *VC1467* (TLC2), *VC1468* (TLC3), *VC1469* (TLC4), and *VC1470* (TLC5) of N16961 (NC_002505.1) were used as reference genes.

#### Prediction of the Enterotoxin Related Components

CTX prophages have been classified as CTX^*cla*^, CTX-1, CTX-2, CTX-3, CTX-3b, CTX-4, CTX-5, CTX-6, CTX-6b, CTX^*AUS*^, CTX^*US*–*Gulf*^, and CTX^*O*139^. Twelve alternative alleles of *ctxB* genes, three alternative alleles of CT regulatory genes (*zot*, *ace*, *orfU*, and *cep*), nine alternative alleles of *rstB* genes, seven alternative alleles of *rstA* genes, and four alternative alleles of *rstR* genes were used for precise prediction (Supplementary Table 2). Each gene profile was used to identify detailed alternative alleles of the extracted CDSs (Figure 1B). The CTXscan extracts all component genes from the query genome with criteria of length coverage > 90%, sequence identity > 90%, and *e*-value < 1e—5. In the arrangement of TLC, CTX prophage, and RS1, if all CDS components are present, they are denoted as :TLC: (serially containing TLC1, TLC2, TLC3, TLC4, and TLC5), :CTX: (*rstR*, *rstA*, *rstB*, *cep*, *orfU*, *ace*, *zot*, *ctxA*, and *ctxB*), and :RS1: (*rstR*, *rstA*, *rstB*, and *rstC*). To predict the chromosomal location of the genetic elements, the genome sequence of N16961 (GCA_000006745.1) was used as a reference for comparison. Using the USEARCH program, local alignment of the contig to which the elements belong was performed on the large chromosome (chromosome I; NC_002505.1) and small chromosomes (chromosome II; NC_002506.1) of N16961, and the max-length matching result was used as the prediction result.

### Prediction of Antibiotic Resistance

For antibiotic resistance gene detection, the Resistance Gene Identifier (RGI) program in the CARD database was used (Jia et al., 2016). The RGI program finds resistomes using amino acid sequences from the CARD database. This study was conducted using the “Perfect” and “Strict” algorithms of the RGI program, and the main data were only analyzed using the “Perfect” algorithm.

### Prediction of SXT (SXTscan)

The SXTscan extracts the SXT element sequence using the SXT-chromosome right junction (*attR*: ATCATCTCGCACCCTGA) and left junction (*attL*: TCGCGATCATCTCGCACCCTGA) (Hochhut and Waldor, 1999). A single contig with both junctions, with a space of more than 1 kb, was selected as the SXT element sequence. Prodigal v2.6.1 and Swiss-Prot databases (O’ Donovan et al., 2002) were used to predict the ORFs. To reduce false-positive annotations, all alignment pairs for which percent similarity was lower than percent identity were discarded, and 35% similarity and 60% query coverage were used as the minimum threshold following the guideline (Rost, 1999).

### Prediction of VPIs and VSPs (VSPIscan)

To predict GIs, VSPIscan uses the genome sequences of *V. cholerae* N16961, which contains all known VPIs and VSPs. Local alignment against the reference GI genes was performed with identity and length coverage > 90%, as determined by a heuristic approach. The list of ORFs of the four GIs used as a reference is shown in Supplementary Table 3.

## Results

Among the genome dataset, OAGCs were identified in 601 genomes, while CTX-related elements (TLC, CTX, and RS1) were identified in 593 genomes. A single OAGC was allocated to each of 47 serogroups—12 previously assigned non-O1/O139 serogroups (O12, O14, O16, O27, O37, O39, O49, O65, O77, O80, and O144) and 35 non-O1/O139 serogroups that were not phenotypically assigned previously. Although a single OAGC should be allocated to O1 and O139 serogroups, 19 and two variations in OAGC were identified among the genomes of O1 and O139 serogroups, respectively (described below). The results of the O serogrouping and prediction of CTX prophage-related elements are summarized in Supplementary Table 4 and Supplementary Figure 1.

### O-Antigen Serogroups

**FIGURE 2:**
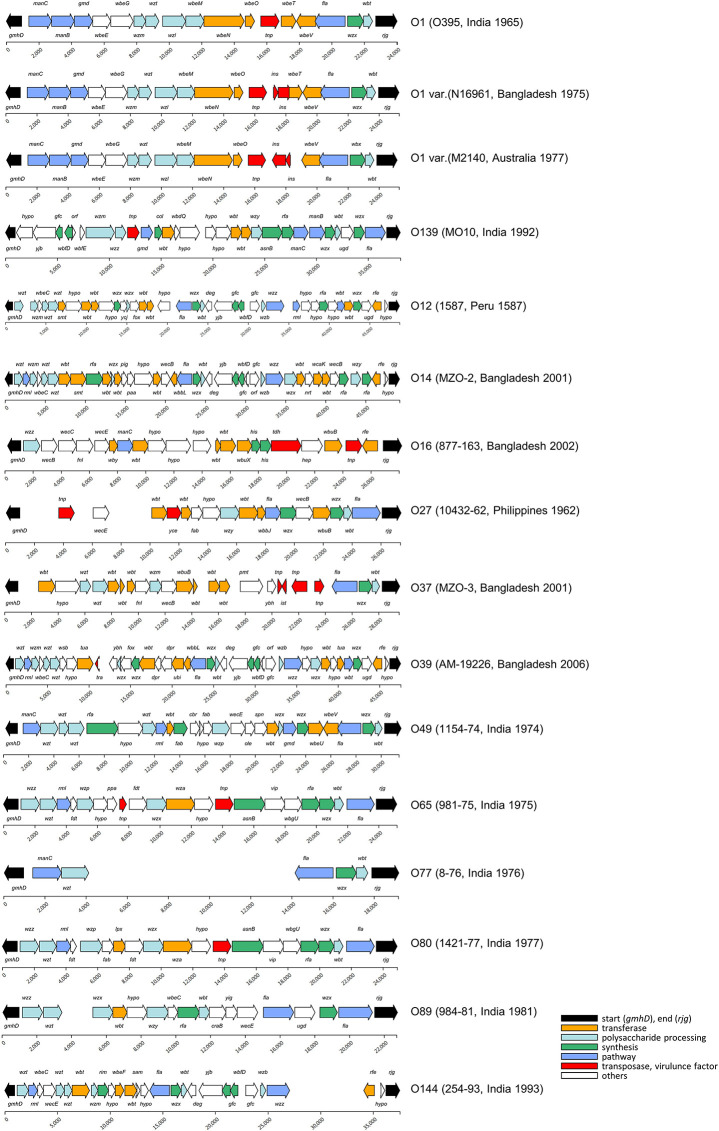
O-antigen gene cluster of the *V. cholerae* strains. A total of 14 known O serogroups and 35 non-O1/O139 were analyzed in this study. Most of the OAGC structures of the O1 serogroup were the same as that of O395, and the structural variants in the O1 serogroups were found at the 13th–15th CDS of the OAGC. The sources of the structural variants were mainly an insertion of mobile elements. The OAGC structures of O139 were mostly the same as that of MO10. The blank space indicates the genomic region where no CDS was assigned from the prodigal program.

To construct standard gene sets (745 genes), 13,561 CDSs were extracted from 604 genomes that had full OAGC. Forty-nine O serogroups with corresponding OAGC were analyzed using the Jaccard index (JI). OAGC of O1 and O139 serogroups, as well as 12 previously assigned non-O1/O139 serogroups were identified from the analyses (Figure 2). In addition, 35 OAGCs that had not been experimentally assigned to known serogroups were inferred from the genome sequences. The 35 newly inferred OAGCs were temporarily named serially Oa1–Od8 (Supplementary Figure 2). Nineteen and two variants were identified in O1 and O139 serogroups, respectively. Overall, 305 previously unassigned genomes were predicted in this study. Among the 296 previously assigned genomes, 293 matched with the existing assigned serogroups while three were confirmed to differ from the existing named serogroups. Three O1-assigned genomes (87395, GCA_000348085.2; EM-1676A, GCA_000348345.2; 2012HC-25, GCA_000788775.1) were identified as non-O1/O139, and we interimly designated the serogroups of 87395, EM-1676A, and 2012HC-25 as Oa2, Oc8, and Od5, respectively.

#### O1 Serogroup

Of the 601 genomes, 504 were named O1 serogroups, and 19 alternative structural variants were found. The group with the most frequent O1 structure was named “O1,” and the group with alternative variants were designated “O1 var.” Therefore, there was one representative O1 and 18 O1 var serogroups. The representative O1 serogroup contained 19 CDSs, including O395 (1965, India) (96%, 484 of 504), and the length of the OAGC was approximately 24.5 kb. Next, the O1 var group to which N16961 belongs had four genomes and consist of 21 CDSs. The structural difference between O1 and O1 var is the transposase IS family, *insO* and *insN* existing between the transposon element locus (*dde*_*yhhI*) and *wbeT* locus. One or two genomes belonged to the remaining O1 var serogroups, and the alternative O1 variant with the lowest number of CDSs (13 CDSs) within the OAGC was the O1 var serogroup containing GP143 (1978, Bahrain). The CDS counts in the O1 serogroup ranged from 13 to 21.

#### Serotypes Within the O1 Serogroup

A total of 35 alleles of the *wbeT* gene were extracted after removing the redundant sequences. The Ogawa serotype includes three classical Ogawa serotypes (M66-2, O395, and M29), and four El Tor Ogawa serotypes (MG116226, A152, 2010EL-1749, and 6/67). Twenty-eight Inaba serotypes were also identified (Supplementary Table 5). Of the 504 O1 genomes, there were 405 Ogawa and 93 Inaba. Six genomes could not be analyzed due to the lack of the *wbeT* gene in the *wbeT* locus, strongly suggesting that the six genomes were Inaba. Some alleles producing the Inaba serotype had single nucleotide changes, while other alleles had an insertion event in front of the *wbeT* gene. There were 23 cases in the group with changes at a single nucleotide level, with 13 in/del and 10 substitutions including four nonsense and six amino acid changes. The second group contained five alleles of the two types. One type in which *wbeT* is partially lost, the A325 type (the other part could be compared with Ogawa types at the nucleotide level), has the transposase located at the 81st position of *wbeT*. The other type, including the N16961 allele and three alleles, in which the transposase is located at 469 bp, has a chimeral *wbeT* gene (Supplementary Table 5). The one allele included A325 (1993, Argentina), whereas the other allele included N16961 IEC224 (1990, Brazil), I-1471 (2011, Russia), I-1300 (1999, Russia), and 2132 (1999, Tanzania).

#### O139 Serogroup

There were two types of O139. One type was O139 serogroup where MO10 belonged to and the other was A330 type which had one fewer CDS than the MO10 type. Overall, 21 genomes belonged to the MO10 type, while one belonged to the A330 type.

*Other serogroups*. We identified 47 non-O1/O139 serogroups, including 12 serogroups (O12, O14, O16, O27, O37, O39, O49, O65, O77, O80, O89, and O144) and 35 different non-O1/O139 serogroups. The serogroups of 35 non-assigned non-O1/O139 strains were serially named Oa1 to Od8.

### CTX Prophage and Related Elements

The CTX and related elements were predicted using 15 standard gene sets (*rstRAB*, *cep*, *orfU*, *ace*, *zot*, *ctxAB*, *rstC*, and TLC1-5) extracted from 796 genomes. The alleles to be predicted within the genome and the arrangement and location of each element constituting the phage-origin genetic elements (TLC, CTX, and RS1) were investigated. Of these, 571 genomes were identified to have at least one of the *ctxB*, *cep*, *rstR*, *rstA*, and *rstB* genes, which are known to be useful for CTX analysis.

**FIGURE 3:**
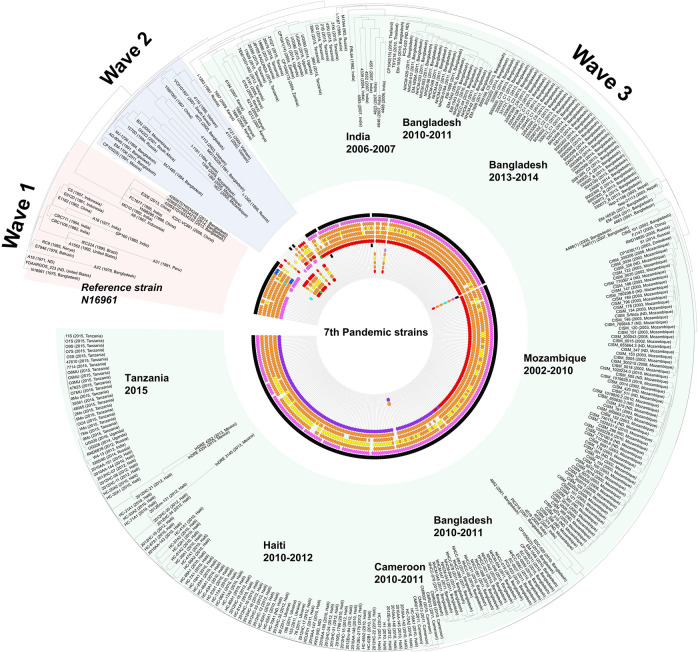
Distribution of *V. cholerae* 7th pandemic strains harboring various TLC, CTXφ, and RS1. The distribution of the CTX prophage and related genetic elements was overlaid on the SNP based on maximum likelihood tree which was constructed using the PhaME tool. The various phage-origin elements were colored red (classical type and *ctxB1*), orange (CTX-1 and *ctxB3*), yellow (CTX-2), blue (*ctxB4*, *ctxB5*, or *rstR*^*CTX*– *O*139^), purple (*ctxB7*), sea green (US Gulf), pink (RS1 elements), and green (else type). The black color indicates the presence of TLC. The displayed marks of the elements are sequentially drawn from chromosome I (TLC > *rstR* > *rstA* > *rstB* > core (*cep*, *ace*, *zot*, *orfU*) > *ctxB* > RS1) to chromosome II. For the wave 3 strains, the order of the elements is TLC > RS1 > *rstR* > *rstA* > *rstB* > core > *ctxB*.

*Seventh pandemic strains*. The CTX-related phage-origin genetic elements of the 7th pandemic strains are shown in Figure 3. However, owing to the complexity of the elements, Figure 3 only shows the *ctxB* alleles, CTX-core (*cep*, *rstB*, *rstA*, *rstR*), and the existence of RS1 and TLC. Detailed information on these elements is presented in Supplementary Table 4. Among the 7th pandemic strains, 46 genomes were predicted as the wave 1 or wave 2 types. Notably, one wave 2 strain, CP1032 (1991, Mexico), was identified on the North American continent. It harbors *ctxB1* in:CTX-2: on chromosome II and:TLC:RS1: on chromosome I. Most of the genome data of the 7th pandemic strains show characteristics of the Wave 3 types.

The predicted *ctxB* alleles of the O139 strains were *ctxB3*, *ctxB4*, and *ctxB5*. Among the 21 O139 strains, 12 genomes were predicted to harbor *ctxB3*: one was *ctxB4*, six were *ctxB5*, and three harbored *ctxB4* and *ctxB5* together. The genomes harboring CTX-O139, referred to as CTX-Calcutta (Davis et al., 1999), were also found. Although prediction of CTX-O139 uses only one gene (*rstR*^*O*139^; synonym *rstR*^Calcutta^), eight genomes harboring *rstR*^*O*139^ were found. Notably, the genomes harboring *ctxB4* and *ctxB5* were predicted to have *rstR*^*O*139^. The *rstR*^*O*139^ was mostly found in the genome harboring *ctxB5* (7/8).

MO10, a representative of the O139 strain, had the:TLC:CTX: array on chromosome I, *ctxB* was of the *ctxB3* type, and the other elements were of the CTX-1 type. Similar to the Wave 1 strain, the O139 strain with RS1 was also identified. E306, isolated in 2013 in China, had an array of:TLC:CTX:RS1: on chromosome I, and the type of *ctxB* allele was *ctxB3*, which is similar to that of MO10. All three strains that contained *ctxB4* and *ctxB5* together were identified to have a RS1 element.

*CTX in non-O1/O139 strains*. Enterotoxin-related elements were also found in non-O1/O139 serogroup strains, including O37, O75, and O141. Two O37 strains [FDAARGOS_102 (1963, India) and V52 (1968, Sudan)], eight O75 strains [CP1110, CP1111, CP1112, CP1113, CP1114, CP1115, CP1116, CP1117 (2011, US)], and one O141 strain [V51 (1987, US)] harbor the *ctxB* gene. The O37 strains harbor *ctxB9* (FDAARGOS_102) or *ctxB8* and *ctxB9* (V52). The O75 strains have *ctxB1*, *rstB*^*CTX*–*USGulf*^, *rstA*^*CTX*–*USGulf*^, and *rstR*^*CTX*–*O*139^/*rstR*^*CTX*–*USGulf*^. The O141 strain, V51, was similar to the O75 strain. V51 harbors *ctxB1* and the CTX-USGulf types of other elements.

*CTX^*AUS*^ types*. There is currently no clear information about the exact alleles in each CTX element of the CTX^*AUS*^ type, besides *rstR* and *ctxB*. The M2140 (1977, Australia) was identified to have an array of:TLC:TLC:CTX:CTX: on chromosome I and harbor *ctxB2*. These are typical CTX^*AUS*^ types with *rstR*^*cla*^. BX330286 (1986, Australia) harbors the:TLC:CTX: array and the genes *rstA*, *rstB*, and *rstC* are found on chromosome I containing *ctxB2* and *rstR*^*cla*^. The CTX^*AUS*^ types were identified to have one or two:TLC:CTX: arrays on chromosome I and harbor *ctxB2*, *rstR*^*cla*^, *rstA*^*CTX*–2^, *rstB*^*CTX*–1^, *cep*^*CTX*–1^, *ace*^*USGulf*^, and *zot*^*CTX*–1^. The genomes of the *ctxB2* type are two Australian strains and R17644 (1997, Russia). In particular, R17644 had both *ctxB1* and *ctxB2*, which might be associated with the CTX^*AUS*^ type. As the genome quality of R17644 is poor (137 contigs, N50 118,411), a remarkable amount of information has been partially lost. Accordingly, we could not identify any intact genetic elements. However, *ctxB1*, *ctxB2*, *zot*, *rstA*, *rstB*^*USGulf*^, and partial TLC were found in different chromosome I contigs. As the CTX type of *rstB* is different from that of Australia, it could not be determined as CTX^*AUS*^ (Supplementary Figure 3).

### Antibiotic Resistance

The rate of antibiotic resistance was analyzed using 734 genomes with isolation year information. The subsequent results were based on the “Perfect” prediction option in the RGI program of CARD. The Perfect algorithm detects antimicrobial resistance proteins with 100% match to a curated reference in the CARD database. Of note, the “Strict” algorithm does not use exact matching with the reference database, allowing for variation from the CARD database within the curated BLAST bit score cutoffs (Jia et al., 2016).

#### Antimicrobial Resistance Ratio of *V. cholerae*

Of all the genomes analyzed, 607 strains (76%) were predicted to be resistant to at least one of the 17 antibiotics (Table 1). More than 50% of the strains were predicted to be resistant to carbapenems, phenicols, sulfonamides, or sulfones. Approximately 71% (563 of 796) of the strains were predicted to be resistant to carbapenem. Of the 563 carbapenem-resistant strains, 561 (99%) were estimated to have subclass B1 *varG* β-lactamase, while the rest were identified as encoding the New Delhi Metallo (NDM) β-lactamase with multidrug resistance. A total of 65% of *V. cholerae* were predicted to be resistant to phenicol antibiotics with chloramphenicol acetyltransferase (CAT). Furthermore, 53% of *V. cholerae* were predicted to be resistant (antibiotic target replacement) to sulfonamide antibiotics as they harbored *sul*. In addition, 5% of the *V. cholerae* were predicted to be resistant to fluoroquinolone antibiotics, 2% to penicillin antibiotics, and 2% to macrolide antibiotics by harboring quinolone resistance-determining regions (QRDRs), metallo-β-lactamase gene (MβL; *varG*), and efflux-pump genes (*varACDEF*), respectively.

**TABLE 1:** Drug resistance predicted from the *V. cholerae* genomes.

	Antibiotics class	Resistance	
		Perfect	Strict
1	Carbapenem	563	145
2	Phenicol	521	49
3	Sulfonamide	425	28
4	Sulfone	425	28
5	Fluoroquinolone	39	756
6	Penam	18	777
7	Cephalosporin	13	27
8	Macrolide	12	783
9	Monobactam	11	19
10	Penem	11	17
11	Streptogramin	5	1
12	Aminoglycoside	3	460
13	Cephamycin	3	2
14	Diaminopyrimidine	3	440
15	Nucleoside	2	0
16	Rifamycin	2	3
17	Tetracycline	1	124
18	Aminocoumarin	0	1
19	Glycopeptide	0	3
20	Glycylcycline	0	3
21	Triclosan	0	2

*The RGI program was used for antibiotic resistance prediction.*

#### Trends in the Antimicrobial Resistance of *V. cholerae*

**FIGURE 4:**
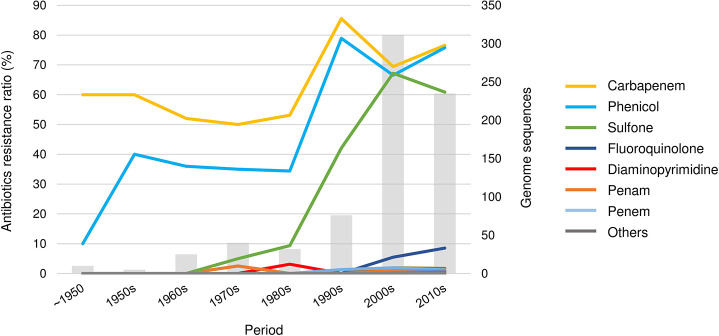
The trend of antibiotic resistance of *V. cholerae*. A total of 21 drug classes were predicted. The drugs in the “others” category include aminocoumarin, cephamycin, glycylcycline, streptogramin, triclosan, cephalosporin, glycopeptide, nucleoside, rifamycin, tetracycline, monobactam, macrolide, and aminoglycoside.

The antibiotic resistance of the cholera bacteria markedly increased (Figure 4). The resistance rate, for example, of carbapenem and phenicol remained steady at approximately 35–50% until the 1980s; however, both showed a steady increase between the 1980s and the 1990s and reached 75% of resistance. Since then, the resistance rate has remained high. However, the trends of antibiotic resistance observed in this study may due to the genome sequencing bias.

*Multidrug resistance*. *V. cholerae* was resistant to an average of 2.58 classes of drugs. Strain 2012EL-2176 isolated in Haiti in 2012 showed the highest multi-drug resistance by having resistance to 11 classes of drugs. Of the 796 genomes analyzed, 44 (6%) showed resistance to more than five drugs. Of these 44 strains, most were isolated after 2000, except for two strains, A10 (1979, Bangladesh) and RC9 (1985, Kenya), and 20 were isolated after 2010. In particular, all the strains isolated from the 2012 Haiti epidemic (2012Env-94, 2012Env-131, 2012HC-21, 2012HC-24, 2012HC-31, 2012HC-32, 2012HC-33, and 2012HC-34) showed resistance to at least five classes of antibiotics.

### SXT Elements and GIs

**FIGURE 5:**
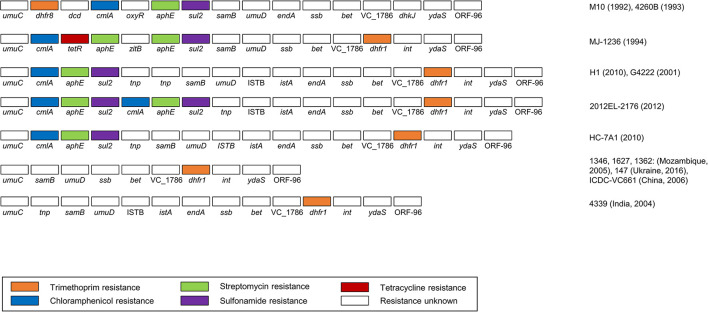
The gene contents and structures of eight types of SXT elements predicted to be resistant to several antibiotics. The drug-resistance genes are highlighted in the following colors: orange (trimethoprim), blue (chloramphenicol), green (streptomycin), purple (sulfonamide), and red (tetracycline). The representative strains are indicated on the right.

A total of 13 types of SXT elements with a size range of 78,251–99,578 bp were predicted in this study. Among these, seven SXTs were predicted to possess five resistance genes, including *dhfr* (trimethoprim), *cmlA* (chloramphenicol), *tetR* (tetracycline), *aphE* (streptomycin), and *sul2* (sulfonamide) (Figure 5). The genomic contents and structures of the remaining six SXT elements with no antibiotic resistance genes are summarized in Supplementary Figure 4.

In this study, the cases of TCP (—) and CT (+) were confirmed using their genome data. Strain E9120 is TCP (—) and contains an array of:TLC:TLC:CTX: on chromosome I, and the *ctxB* gene type was identified as *ctxB3*. Of note, strain 3582-05 is also TCP (—) and was predicted to contain *ctxA*, *orfU*, *zot*, *rstB*, partial TLC (TLC2, TLC4), and *ctxB* (*ctxB1*) on chromosome I; however, the gene arrangements were not determined. An additional TCP (—) and CT (+) strain, EM-1706 (2011, Bangladesh, O1 Ogawa), was also found to contain *rstR*, *rstA*, *rstB*, and partial TLC (TLC4 and TLC5) on chromosome I while CTX (the *ctxB* allele type is *ctxB1*), *rstA*, and *rstB* were found to be on chromosome II.

## Discussion

### O-Antigen Serogroup Prediction

#### O-Antigen Serogrouping

Of the 601 genomes identified as having full OAGCs, 83.4% (504) were predicted to be O1 serogroups. Of the 504 O1 types, 484 (96%) strains had the same structure as the classical biotype strain, O395.

There were 18 O1 variants, of which 16 were Inaba. A representative example of O1 var Inaba is El Tor biotype N16961. Compared to the classical structures, the OAGC structure of N16961 had one more transposon element at the front part of *wbeT*; hence, the overall OAGC structure was slightly different. In addition, because the corresponding transposon element is located at the front part of *wbeT*, the function of the gene was lost, resulting in an Inaba type. For N16961, approximately 2 kb, including two IS3 family members, *ins*, was inserted in the positive direction. Thus, assuming that all the CDSs within the OAGC of the O1 serogroup had callings, the total number of CDSs was determined by the presence or absence of influx of the transpose family gene located at the front of *wbeT*. Furthermore, for M2140 (1977, Australia), two genes, *insO2* and *insN1*, of approximately 2,500 bp were inserted in the negative direction from the end of *dde_yhhI* to the forepart of *wbeT*; thus, *wbeT* was not detected as CDS. It was presumed that a site exists where a transposable element is easily inserted between the transposon upstream of *wbeT* and the front part of *wbeT*, acting as a decisive factor for O1 serotype conversion.

Of the 21 O139 strains that occupy an important position in the seventh cholera pandemic, 20 genomes had the same structures as the OAGC of O139 MO10 (1992, India). The O139 var was A330 (1993, India) and showed that *wbdQ* was not found at the 16,310 bp position downstream of the OAGC start site. As *wbdQ* encodes GDP-mannose mannosyl hydrolase, its deletion can affect the structure of the O-antigen. The reason for this is unknown as the two adjacent genes were assigned as hypothetical proteins.

In addition to the OAGC regions included in this study, several OAGC regions, including O5, O8, and O31, have been reported by PCR and Sanger sequencing (Blokesch and Schoolnik, 2007; Chen et al., 2007; Aydanian et al., 2011). Because these sequences are incomplete in the two flanking genes of the O-antigen gene cluster, *gmhD* and *rjg*, we could not include them in the current analyses, which only included complete sequences. Thus integration of PCR-based sequences into the VicPred was impossible under the current gene prediction threshold.

#### Prediction of the O1 Serotype

Six genomes previously assigned to Ogawa were newly identified as Inaba in this study. In these cases, as the nonsense mutation of *wbeT* is the cause of all six cases, wrong serotyping could occur as an experimental error or a sequencing error. In contrast, 36 genomes previously assigned to Inaba were identified as Ogawa in this study. There would be a discrepancy between the genotype and phenotype as the 36 genomes have the Ogawa type of *wbeT*. In addition, as the WbeT protein is a methyltransferase, methyl may not have been properly delivered to the sugar moiety because of a mutation downstream of the signal pathway involving methyltransferase. Of note, there is a discrepancy between the phenotype and genotype; however, the reasons for this discrepancy are unclear.

### CTX-Infection With TCP-Negative and tolQRAB-Positive Strain

Strain E9120 is likely to be infected by CTX with TCP-negative and *tolQRAB*-positive strains (Karaolis et al., 1999). We attempted to find the *tolQRAB* through a local alignment using the *tolQRAB* gene cluster (AF187269.1) of the O395 strain; however, there was no *tolQRAB* cluster, indicating that it is highly likely to be TCP (—) and *tolQRAB* (+) infection. However, due to the poor sequencing quality (88 contigs with N50 of 158,348), the possibility that sequencing was not performed at the location of the gene cluster cannot be ruled out.

### AR Prediction

The Perfect algorithm of CARD predicts resistome within the query genome based on homology with experimentally proven genes and the SNP model (Jia et al., 2016). Strict and “Loose” predict potential antibiotic resistance to the query. In this study, using the Strict criteria, 95% resistance to fluoroquinolone was predicted, which markedly differed from the 5% predicted by the Perfect algorithm. This gap was also found in other drug resistance studies. The underlying mechanism for resistance to most drugs, predicted using the Strict algorithm, was identified as antibiotic efflux. The efflux pump mechanism was related to multidrug resistance in various ways; however, in the criterion Strict, this has not been proven experimentally.

The resistance to doxycycline, a tetracycline family member, was only one case and 16% using the Perfect and Strict algorithms, respectively. However, there have been reports of increased resistance to tetracycline antibiotics in recent years (Bhattacharya et al., 2011). In the case of tetracycline, as predicted with Strict, the main efflux pump mechanism was tetracycline-specific antibiotic efflux, a major facilitator superfamily (MFS) efflux pump. Accordingly, it can be concluded that the results of tetracycline resistance analysis contain information that may be reliably referenced. Therefore, the web application developed in this study contains both the results of the Perfect and Strict algorithms for predicting antibiotic resistance.

### Genomic Islands

The SXT element, an integrating conjugative element (ICE), was initially isolated from a *V. cholerae* O139 clinical isolate from India (Waldor et al., 1996). Since its discovery in 1992, numerous studies have been conducted to describe the diversity of SXT (Dalsgaard et al., 2001; Hochhut et al., 2001; Beaber et al., 2002; Amita et al., 2003; Iwanaga et al., 2004). Using our prediction algorithm, a novel SXT element was found in *V. cholerae* 5473-62, isolated from the Philippines in 1962 (Supplementary Figure 4). Although this SXT element was not predicted to contain drug-resistance genes, it is worth noting that the unknown GIs can be found using only the computational prediction method. Therefore, it is expected that currently unknown GIs will be discovered using the program developed in this study. However, when the criteria for determining ORFs in this study were employed, relatively conserved genes in the SXT-ICE family, such as the *int* gene (encoding an integrase) and *tra* gene (encoding the ICE conjugation apparatus), were not predicted. Despite this shortcoming, we could extract ICE from *prfC* and predict most of the reported antibiotic resistance genes.

## Genome-Based Diagnostics

The genomes of *V. cholerae* were largely sequenced in the early 2000s with the advancement of the NGS technology at the time, which meant that the quality of the sequences was mostly poor. As a result, only 44 complete genomes (consisting of two contigs as *V. cholerae* contains two chromosomes) were available in this study, and the average number of contigs for all assemblies was 109.26. The quality of the genome data is an important issue in prediction algorithms that refer to the existing genomic contents. Thus, the VicPred tool will continue to be updated with accumulated data to improve the sustainable research environment and prediction accuracy.

The next-generation sequencing technology is currently being used in almost all traditional microorganism analyses, from the identification of single microorganisms to the characterization of microbial communities. However, as sequencing is more expensive and yield less reliable results than *in vitro* molecular diagnostic tests, it has not completely replaced the existing molecular diagnostic tests. Nonetheless, researchers have predicted that many molecular diagnostic tests will soon be replaced with WGS-based analysis as the diagnostic resolution of WGS-based genotype prediction analysis is currently outperforming existing methods. Currently, WGS-based analysis is replacing the existing tests in many areas.

Bioinformatics approaches are indispensable tools for biological research, especially in genomics studies of various organisms. Many parts of biological research, such as data collection, analysis, modeling, and simulation, can be performed in a computer-aided manner. Although some research fields cannot be significantly facilitated by computer-aided methods, *in silico* approaches have played critical roles in improving the efficiency and accuracy of biological research. Collaboration and alliances between experiment-based research and bioinformatics will have synergistic effects in various areas of microbial research.

VicPred was developed as a genotype prediction tool for *V. cholerae* genome sequences. However, the *in silico* analysis results might not coincide with the experimental phenotype, which might be due to insufficient accuracy of the sequencing data and mistaken phenotype data collected. Accumulation of more genome sequence data with corresponding experimental evidence in VicPred will improve the accuracy of the genome analysis, ultimately enabling the advancement of this database as a useful tool for research on *V. cholerae.*

## Data Availability Statement

The datasets presented in this study can be found in online repositories. The names of the repository/repositories and accession number(s) can be found in the article/Supplementary Material.

## Author Contributions

IL, DK, and JC contributed to the conception and design of the study. IL performed data analyses and developed the V.O.S., CTXscan, SXTscan, and VSPIscan. IL and S-MH developed a VicPred web application platform. DK, HY, and JC supervised the study. IL, M-gB, HY, and DK wrote the manuscript. All the authors read and approved the final manuscript. All authors contributed to the article and approved the submitted version.

## Conflict of Interest

The authors declare that the research was conducted in the absence of any commercial or financial relationships that could be construed as a potential conflict of interest.

## Publisher’s Note

All claims expressed in this article are solely those of the authors and do not necessarily represent those of their affiliated organizations, or those of the publisher, the editors and the reviewers. Any product that may be evaluated in this article, or claim that may be made by its manufacturer, is not guaranteed or endorsed by the publisher.

## Footnotes

1
http://vicpred.hanyang.ac.kr
