# Supplementary material for: VicPred: A Vibrio cholerae Genotype Prediction Tool
Source: Front Microbiol. 2021 Sep 9;12:691895. doi: 10.3389/fmicb.2021.691895 (PMC8458814; doi:10.3389/fmicb.2021.691895)
Supplement: Supplementary file 1 [file Data_Sheet_1.docx]

**Supplementary Figure legends**

**Supplementary Figure 1. The CTX prophage-related elements of the *V. cholerae* strains.** The CTX prophage-related elements are described on the Maximum likelihood phylogenetic tree based on the SNP differences in the whole genome. The presence of CTX prophage, TLC, and RS1 element on each chromosome was discerned mostly among the O1 and O139 serogroup strains; however, partial TLC, CTX, and RS1 were also found in some of the O105, O141, and O75 serotype strain.

**Supplementary Figure 2. Variants of the non-O1/O139 strains.** A total of 35 non-O1/O139 were analyzed in this study. The OAGCs of non-O1/O139 that were not experimentally assigned were distinguished and named serially, Oa1 to Od8. The blank space indicates the genomic region where no CDS was assigned from the prodigal program.

**Supplementary Figure 3.** **Predicted genetic structure of CTX harbored in M2140 that has a representative CTX^AUS^ type, CTXφ.** CTX prophage array composed of a tandem repeat of CTX^AUS^ in strain M2140. The genome of M2140 harbors two TLC and two CTX prophages (*rstR*^cla^, *rstA*^CTX-2^, *rstB*^CTX-1^, and *ctxB2*).

**Supplementary Figure 4. The gene contents and structures of the six non-AR SXT elements of *V. cholerae*.** A total six of SXT elements non-harboring antibiotic resistance genes were predicted. The representative strains are described on the right side.
