# Supplementary figures and images for: VicPred: A Vibrio cholerae Genotype Prediction Tool
Source: Front Microbiol. 2021 Sep 9;12:691895. doi: 10.3389/fmicb.2021.691895 (PMC8458814; doi:10.3389/fmicb.2021.691895)

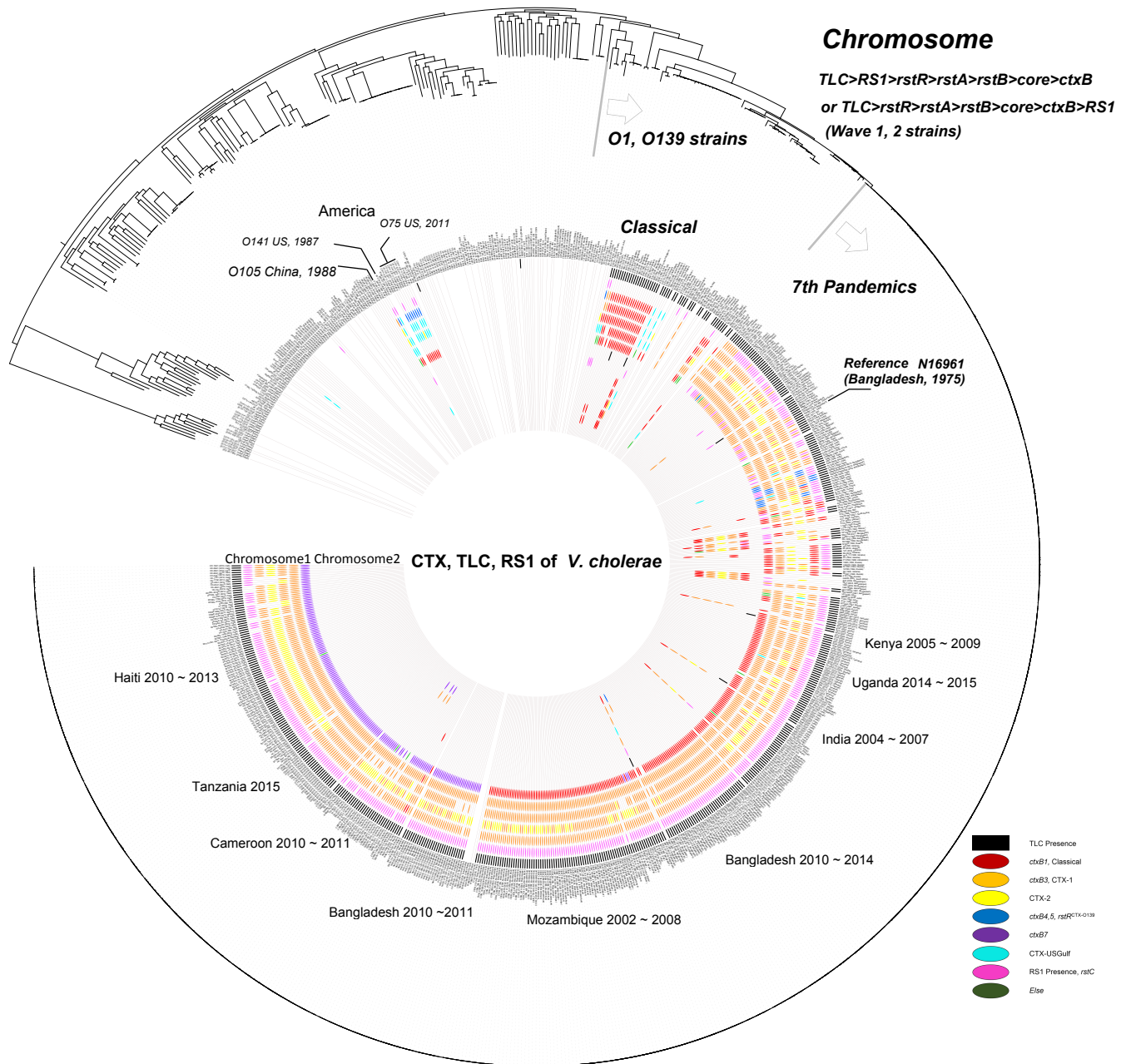

Supplement: Supplementary file 2 [file Image_1.PDF]

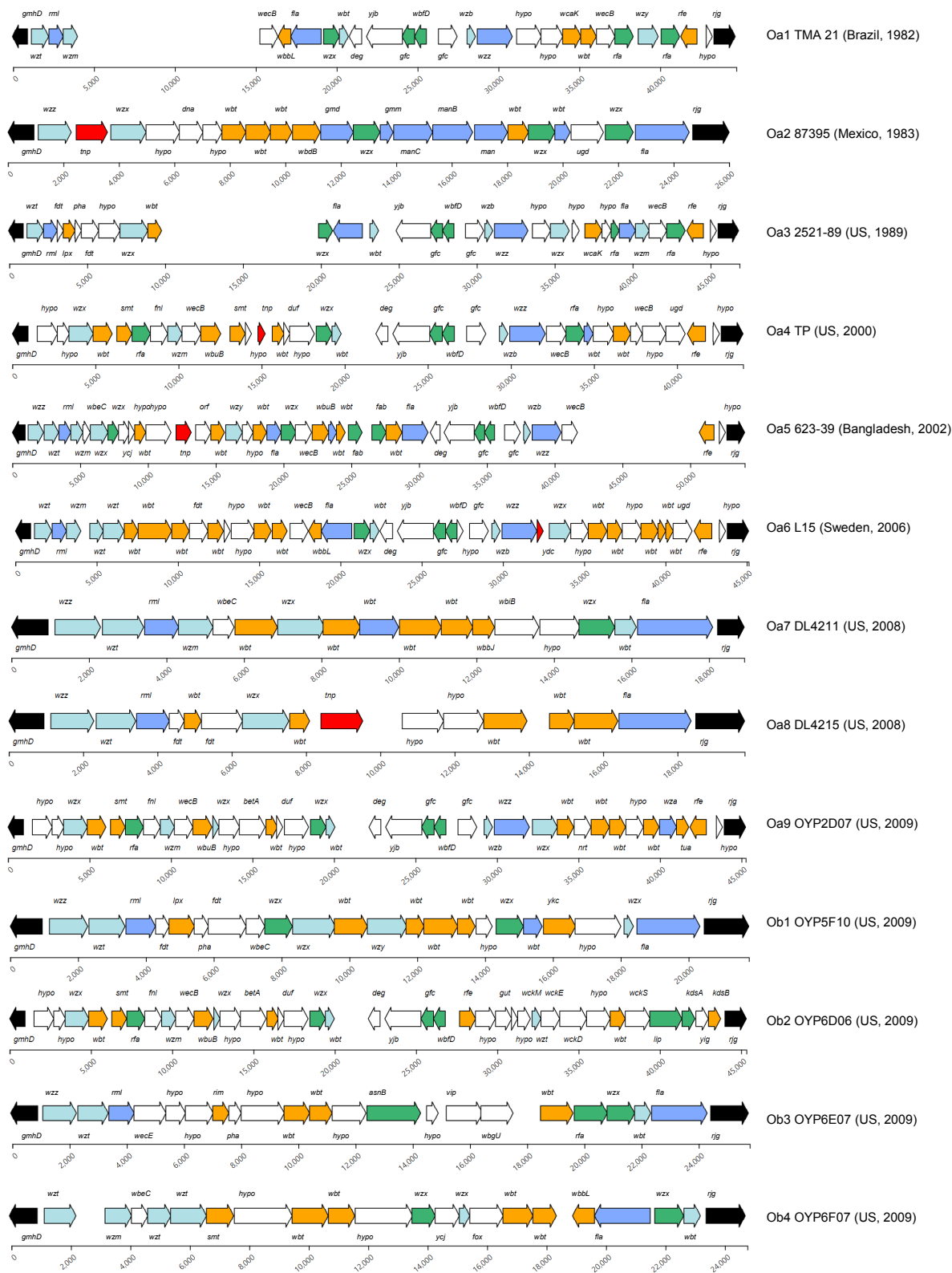

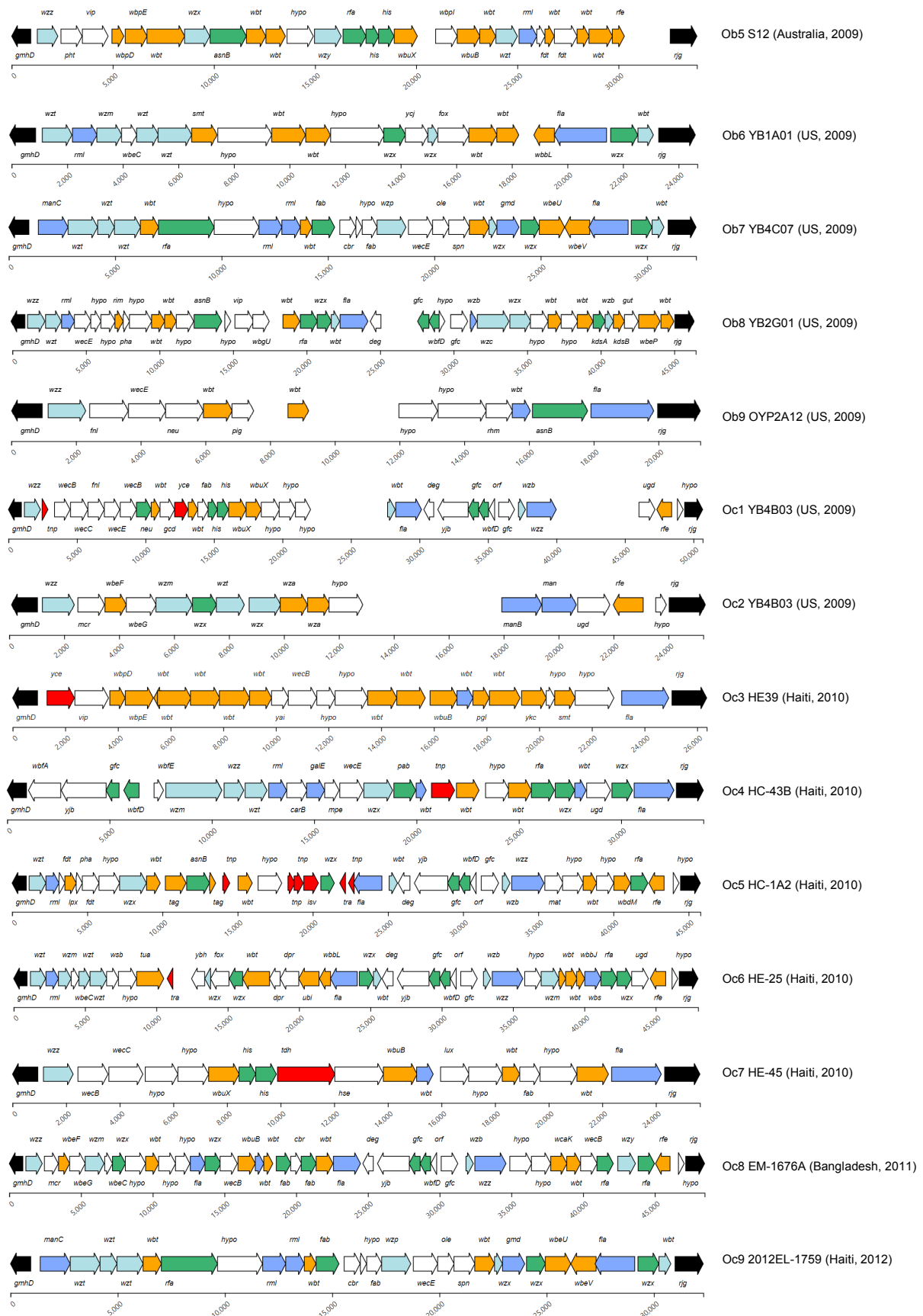

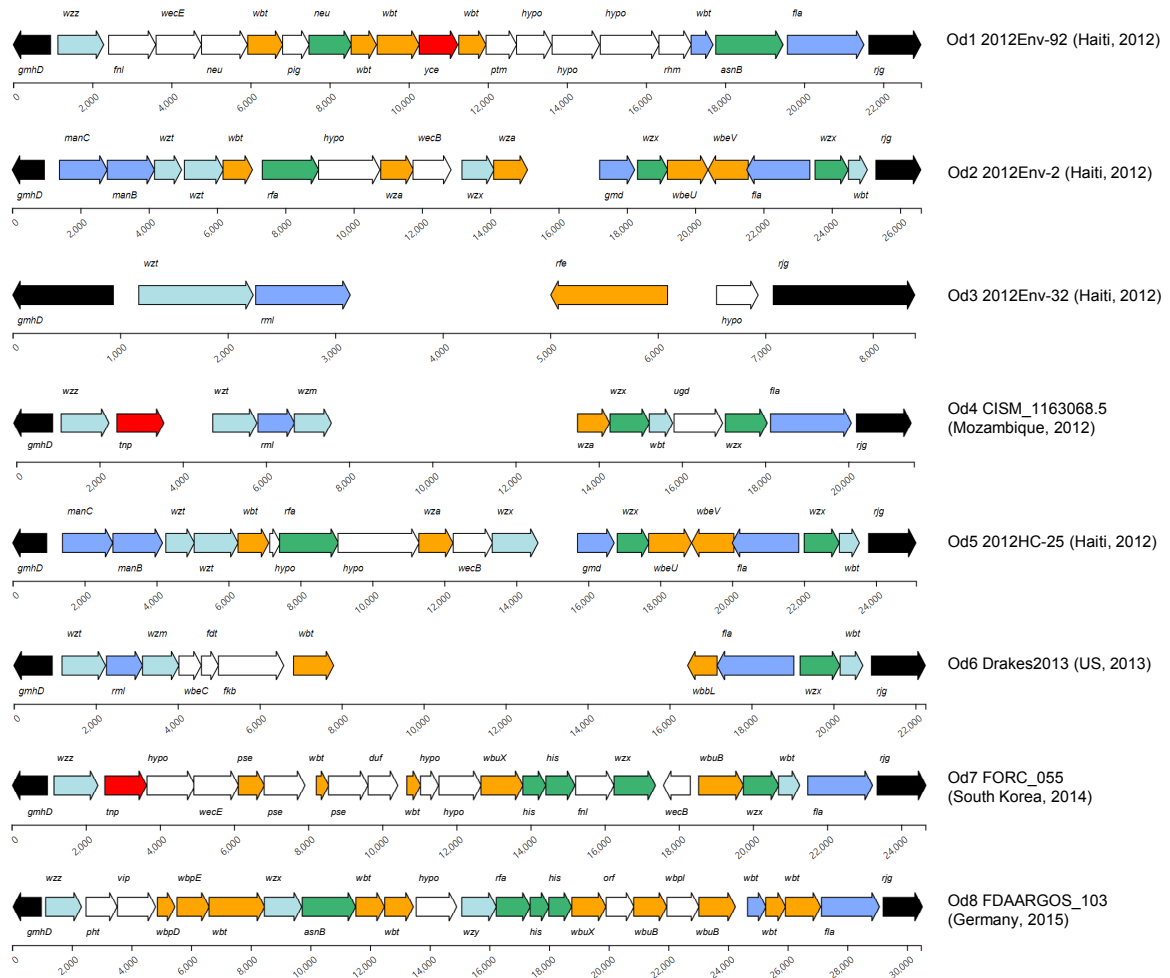

Supplement: Supplementary file 3 [file Image_2.PDF]

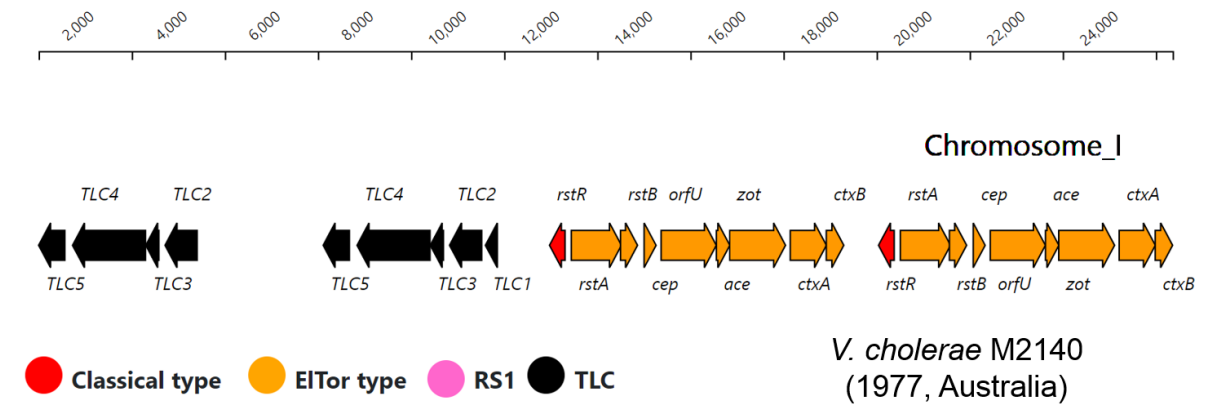

Supplement: Supplementary file 4 [file Image_3.PDF]
