# Supplementary material for: VicPred: A Vibrio cholerae Genotype Prediction Tool
Source: Front Microbiol. 2021 Sep 9;12:691895. doi: 10.3389/fmicb.2021.691895 (PMC8458814; doi:10.3389/fmicb.2021.691895)
Supplement: Supplementary file 5 [file Image_4.PDF]

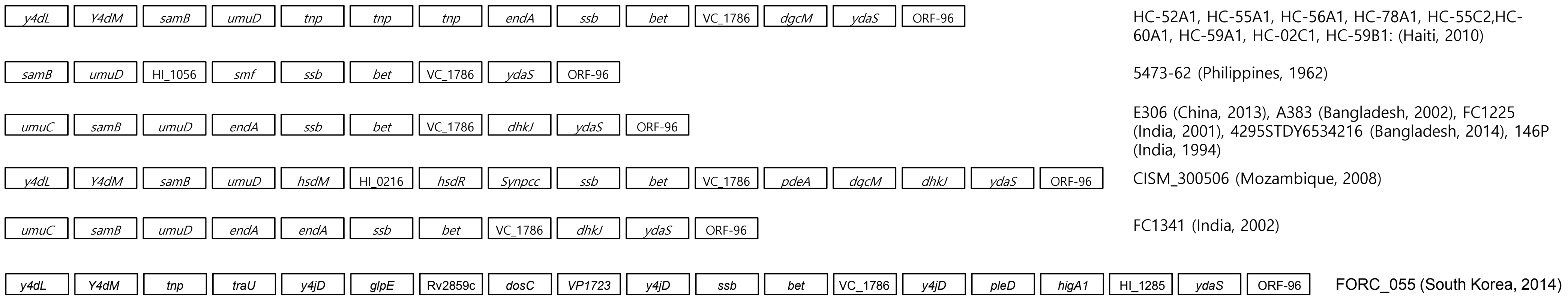

y4dL Y4dM samB umuD tnp tnp tnp endA ssb bet VC_1786 dgcM ydaS ORF-96
HC-52A1, HC-55A1, HC-56A1, HC-78A1, HC-55C2,HC-60A1, HC-59A1, HC-02C1, HC-59B1: (Haiti, 2010)
samB umuD HI_1056 smf ssb bet VC_1786 ydaS ORF-96
5473-62 (Philippines, 1962)
umuC samB umuD endA ssb bet VC_1786 dhkJ ydaS ORF-96
E306 (China, 2013), A383 (Bangladesh, 2002), FC1225 (India, 2001), 4295STDY6534216 (Bangladesh, 2014), 146P (India, 1994)
y4dL Y4dM samB umuD hsdM HI_0216 hsdR Synpcc ssb bet VC_1786 pdeA dgcM dhkJ ydaS ORF-96
CISM_300506 (Mozambique, 2008)
umuC samB umuD endA endA ssb bet VC_1786 dhkJ ydaS ORF-96
FC1341 (India, 2002)
y4dL Y4dM tnp traU y4jD glpE Rv2859c dosC VP1723 y4jD ssb bet VC_1786 y4jD pleD higA1 HI_1285 ydaS ORF-96
FORC_055 (South Korea, 2014)
