# Supplementary material for: VicPred: A Vibrio cholerae Genotype Prediction Tool
Source: Front Microbiol. 2021 Sep 9;12:691895. doi: 10.3389/fmicb.2021.691895 (PMC8458814; doi:10.3389/fmicb.2021.691895)
Supplement: Supplementary file 6 [file Table_1.docx]

**Supplementary Table 1. Information for the genome used in this study.**

| Accession | Strain Name | Year | Country | Genome Size | Num. of Contigs |
| --- | --- | --- | --- | --- | --- |
| GCA_000195065.1 | LMA3984-4 | ND | Brazil | 3738715 | 2 |
| GCA_000021625.1 | O395 | 1965 | ND | 4135300 | 2 |
| GCA_000152425.1 | MO10 | 1992 | India | 4079638 | 27 |
| GCA_000006745.1 | N16961 | 1975 | Bangladesh | 4033464 | 2 |
| GCA_000021605.1 | M66-2 | 1937 | Indonesia | 3938905 | 2 |
| GCA_000022585.1 | MJ-1236 | 1994 | Bangladesh | 4236368 | 2 |
| GCA_000166455.2 | 2010EL-1786 | 2010 | Haiti | 4077740 | 2 |
| GCA_000250855.1 | IEC224 | 1990 | Brazil | 4079586 | 2 |
| GCA_000275645.1 | H1 | 2010 | Haiti | 4089020 | 2 |
| GCA_000338075.1 | G4222 | 2001 | South Africa | 4202811 | 2 |
| GCA_000953755.1 | InDRE 4354 | 2013 | Mexico | 4019937 | 47 |
| GCA_000953775.1 | InDRE 4262 | 2013 | Mexico | 4019893 | 52 |
| GCA_000387585.1 | CP1110 | 2011 | United States | 3925419 | 184 |
| GCA_000387605.1 | CP1115 | 2011 | United States | 3927074 | 183 |
| GCA_000387625.1 | CP1111 | 2011 | United States | 3927492 | 199 |
| GCA_000387645.1 | CP1112 | 2011 | United States | 3927706 | 193 |
| GCA_000387665.1 | CP1113 | 2011 | United States | 3925787 | 186 |
| GCA_000387685.1 | CP1114 | 2011 | United States | 3925539 | 199 |
| GCA_000387705.1 | CP1117 | 2011 | United States | 3925548 | 183 |
| GCA_000387725.1 | CP1116 | 2011 | United States | 3932707 | 207 |
| GCA_000765415.1 | 2012EL-2176 | 2012 | Haiti | 4258023 | 3 |
| GCA_000961975.1 | D-35 | 1958 | Bangladesh | 4010503 | 89 |
| GCA_000818865.1 | I-1471 | 2011 | Russia | 4034973 | 2 |
| GCA_000829215.1 | MS6 | 2008 | Myanmar | 4030944 | 2 |
| GCA_000965285.1 | R17644 | 1997 | Russia | 4020247 | 137 |
| GCA_000966385.1 | M818 | 1970 | Russia | 3982448 | 95 |
| GCA_000966395.1 | P18899 | 2006 | Russia | 4015853 | 132 |
| GCA_000963555.1 | FJ147 | 2005 | China | 4091935 | 2 |
| GCA_000967785.1 | I-1300 | 1999 | Russia | 4033785 | 2 |
| GCA_000969235.1 | 1154-74 | 1974 | India | 3928357 | 1 |
| GCA_000969265.1 | 10432-62 | 1962 | Philippines | 4077462 | 1 |
| GCA_000709105.1 | M29 | 1942 | Russia | 3951052 | 116 |
| GCA_000220725.2 | HC-49A2 | ND | Haiti | 4059369 | 23 |
| GCA_000220785.2 | HE48 | ND | Haiti | 4179415 | 18 |
| GCA_000710155.1 | 2012EL-1759 | 2012 | Haiti | 3982946 | 68 |
| GCA_000710445.1 | RND19188 | 2010 | Russia | 3999500 | 234 |
| GCA_000710455.1 | RND19191 | 2010 | Russia | 4005480 | 179 |
| GCA_000234375.2 | HC-06A1 | 2010 | Haiti | 4028197 | 128 |
| GCA_000234395.2 | HC-23A1 | 2010 | Haiti | 4099188 | 15 |
| GCA_000234415.2 | HC-28A1 | 2010 | Haiti | 4027641 | 138 |
| GCA_000234435.2 | HC-43A1 | 2010 | Haiti | 4053234 | 19 |
| GCA_000304755.1 | CP1033(6) | 2000 | Mexico | 3988132 | 33 |
| GCA_000304775.1 | HC-1A2 | 2010 | Haiti | 3965428 | 19 |
| GCA_000304795.1 | HC-61A2 | 2010 | Haiti | 4000124 | 24 |
| GCA_000305605.1 | HC-46B1 | 2010 | Haiti | 3941918 | 24 |
| GCA_000600255.1 | L-3226 | 2010 | Russia | 3991045 | 123 |
| GCA_000279305.1 | CP1032(5) | 1991 | Mexico | 3971467 | 17 |
| GCA_000279325.1 | CP1038(11) | 2003 | Zimbabwe | 4058268 | 18 |
| GCA_000279245.1 | CP1041(14) | 2004 | Zambia | 4085554 | 23 |
| GCA_000279345.1 | CP1042(15) | 2010 | Thailand | 4045377 | 20 |
| GCA_000281655.1 | CP1046(19) | 1995 | Peru | 4093497 | 25 |
| GCA_000279395.1 | CP1048(21) | 2010 | Bangladesh | 4091990 | 27 |
| GCA_000279415.1 | HC-20A2 | 2010 | Haiti | 4084542 | 27 |
| GCA_000279435.1 | HC-43B1 | 2010 | Haiti | 3931831 | 20 |
| GCA_000279455.1 | HC-46A1 | 2010 | Haiti | 4120976 | 25 |
| GCA_000279265.1 | HE-25 | 2010 | Haiti | 4092530 | 13 |
| GCA_000279285.1 | HE-45 | 2010 | Haiti | 4125202 | 29 |
| GCA_001027485.1 | PhVC-326 | 2011 | Philippines | 3932194 | 71 |
| GCA_001027495.1 | PhVE-5 | 2011 | Philippines | 3929317 | 76 |
| GCA_001027505.1 | PhVC-311 | 2011 | Philippines | 3927966 | 70 |
| GCA_001029975.1 | YN2011004 | 2011 | China | 4057079 | 64 |
| GCA_001030015.1 | YN97083 | 1997 | China | 4074242 | 64 |
| GCA_001030035.1 | YN89004 | 1989 | China | 3950281 | 59 |
| GCA_000221345.1 | HC-40A1 | ND | Haiti | 4031783 | 203 |
| GCA_000221365.1 | HC-48A1 | ND | Haiti | 4032180 | 208 |
| GCA_000221385.1 | HC-70A1 | ND | Haiti | 4057434 | 169 |
| GCA_000221405.1 | HE-09 | ND | Haiti | 3826131 | 223 |
| GCA_000221425.1 | HFU-02 | ND | Haiti | 4032845 | 110 |
| GCA_000221465.1 | BJG-01 | ND | United States | 3910867 | 238 |
| GCA_000221485.1 | HC-38A1 | ND | Haiti | 4036059 | 135 |
| GCA_000234965.1 | HC-19A1 | 2010 | Haiti | 4033401 | 96 |
| GCA_000234945.1 | HC-21A1 | 2010 | Haiti | 4030433 | 96 |
| GCA_000234925.1 | HC-22A1 | 2010 | Haiti | 4034031 | 86 |
| GCA_000234905.1 | HC-32A1 | 2010 | Haiti | 4033209 | 79 |
| GCA_000234885.1 | HC-33A2 | 2010 | Haiti | 4024727 | 64 |
| GCA_000234865.1 | HC-48B2 | 2010 | Haiti | 4024820 | 166 |
| GCA_000304995.1 | HC-50A2 | 2010 | Haiti | 4030802 | 338 |
| GCA_000621645.1 | ATCC 14035 | ND | ND | 4024517 | 62 |
| GCA_000279555.1 | CP1030(3) | 2008 | Mexico | 4010577 | 116 |
| GCA_000302965.1 | CP1037(10) | 2003 | Mexico | 3922570 | 78 |
| GCA_000302985.1 | CP1040(13) | 2004 | Zambia | 4033944 | 105 |
| GCA_000303045.1 | CP1044(17) | 1991 | Peru | 4010199 | 135 |
| GCA_000279785.1 | CP1047(20) | 1995 | Peru | 4039784 | 123 |
| GCA_000303065.1 | CP1050(23) | 2010 | Bangladesh | 4024004 | 114 |
| GCA_000735705.1 | I-1263 | 1997 | Russia | 3957767 | 50 |
| GCA_000302775.1 | HC-39A1 | 2010 | Haiti | 4022430 | 106 |
| GCA_000302755.1 | HC-41A1 | 2010 | Haiti | 4027832 | 116 |
| GCA_000279185.1 | HC-42A1 | 2010 | Haiti | 4024780 | 106 |
| GCA_000279955.1 | HC-47A1 | 2010 | Haiti | 4031718 | 110 |
| GCA_000302835.1 | HC-50A1 | 2010 | Haiti | 3939222 | 126 |
| GCA_000303105.1 | HC-51A1 | 2010 | Haiti | 3929843 | 140 |
| GCA_000302855.1 | HC-52A1 | 2010 | Haiti | 3937542 | 133 |
| GCA_000302875.1 | HC-55A1 | 2010 | Haiti | 3932130 | 129 |
| GCA_000302895.1 | HC-56A1 | 2010 | Haiti | 3934171 | 123 |
| GCA_000279205.1 | HC-56A2 | 2010 | Haiti | 4029365 | 106 |
| GCA_000303005.1 | HC-57A1 | 2010 | Haiti | 3934427 | 125 |
| GCA_000279375.1 | HC-57A2 | 2010 | Haiti | 4025386 | 105 |
| GCA_000303125.1 | HC-81A2 | 2010 | Haiti | 4028931 | 98 |
| GCA_000303085.1 | HE-16 | 2010 | Haiti | 3911386 | 236 |
| GCA_000736765.1 | 133-73 | 1973 | India | 3866097 | 118 |
| GCA_000736775.1 | 984-81 | 1981 | India | 3946950 | 117 |
| GCA_000736785.1 | 1421-77 | 1977 | India | 3969137 | 118 |
| GCA_000736795.1 | 5473-62 | 1962 | Philippines | 3913714 | 94 |
| GCA_000736845.1 | 63-93 (MO45) | 1992 | India | 4018178 | 81 |
| GCA_000736855.1 | 1311-69 | 1969 | India | 3973555 | 130 |
| GCA_000736865.1 | NIH41 | 1941 | India | 3952728 | 111 |
| GCA_000736875.1 | 1157-74 | 1974 | India | 4012216 | 151 |
| GCA_000736925.1 | 981-75 | 1975 | India | 4034493 | 152 |
| GCA_000736935.1 | 8-76 | 1976 | India | 4074796 | 82 |
| GCA_000736945.1 | 571-88 | 1988 | China | 4291048 | 117 |
| GCA_000737005.1 | 234-93 | 1993 | India | 4033463 | 129 |
| GCA_000737015.1 | 490-93 | 1993 | Thailand | 3913946 | 274 |
| GCA_000737025.1 | 254-93 | 1993 | India | 3954364 | 114 |
| GCA_000327105.3 | HC-64A1 | 2010 | Haiti | 4032042 | 93 |
| GCA_000327125.3 | HC-65A1 | 2010 | Haiti | 4028731 | 96 |
| GCA_000327145.3 | HC-67A1 | 2010 | Haiti | 4026751 | 95 |
| GCA_000327165.3 | HC-68A1 | 2010 | Haiti | 4026495 | 90 |
| GCA_000327185.3 | HC-71A1 | 2010 | Haiti | 4028948 | 91 |
| GCA_000327205.3 | HC-72A2 | 2010 | Haiti | 4028422 | 94 |
| GCA_000327225.3 | HC-78A1 | 2010 | Haiti | 3937766 | 133 |
| GCA_000318485.2 | HC-7A1 | 2010 | Haiti | 4074920 | 12 |
| GCA_000327245.3 | HC-80A1 | 2010 | Haiti | 4032382 | 115 |
| GCA_000318505.2 | HC-81A1 | 2010 | Haiti | 4084020 | 16 |
| GCA_000318075.1 | PS15 | ND | United States | 3910387 | 131 |
| GCA_000474965.1 | HC-36A1 | 2010 | ND | 3959428 | 17 |
| GCA_000753725.1 | MAK 676 | 1937 | Indonesia | 3937648 | 117 |
| GCA_000754625.1 | 5/66 | 1966 | Pakistan | 4014060 | 108 |
| GCA_000330905.1 | 4260B | 1993 | Bangladesh | 4039222 | 40 |
| GCA_000705295.1 | M-1293 | 1994 | Russia | 4104797 | 113 |
| GCA_000438805.2 | VCC19 | 1994 | Brazil | 4134889 | 54 |
| GCA_000763075.1 | RND81 | 2014 | Russia | 3956193 | 451 |
| GCA_000166475.2 | 2010EL-1798 | 2010 | Haiti | 4016678 | 93 |
| GCA_000166495.2 | 2010EL-1792 | 2010 | Haiti | 4014995 | 105 |
| GCA_000223095.2 | Amazonia | 1991 | Brazil | 3925563 | 63 |
| GCA_000740515.2 | InDRE 3140 | 2013 | Mexico | 4017985 | 92 |
| GCA_000237405.2 | 2009V-1046 | 2009 | United States | 4014220 | 72 |
| GCA_000237425.2 | 2009V-1085 | 2009 | United States | 4014382 | 73 |
| GCA_000237445.2 | 2009V-1096 | 2009 | United States | 4016479 | 73 |
| GCA_000237465.2 | 2009V-1116 | 2009 | United States | 4013388 | 77 |
| GCA_000237485.2 | 2009V-1131 | 2009 | United States | 4010678 | 94 |
| GCA_000237505.2 | 2010EL-1749 | 2010 | Cameroon | 4009493 | 159 |
| GCA_000237525.2 | 2010EL-1961 | 2010 | Haiti | 4004160 | 96 |
| GCA_000237545.2 | 2010EL-2010H | 2010 | Haiti | 4018270 | 75 |
| GCA_000237565.2 | 2010EL-2010N | 2010 | Haiti | 4019650 | 77 |
| GCA_000237585.2 | 2010V-1014 | 2010 | Haiti | 4015902 | 75 |
| GCA_000237605.2 | 2011EL-1089 | 2010 | Haiti | 4013698 | 87 |
| GCA_000237645.2 | 2011EL-1137 | 2009 | Haiti | 4018022 | 72 |
| GCA_000237665.2 | 2011V-1021 | 2011 | Dominican Republic | 4011850 | 95 |
| GCA_000237685.2 | 3500-05 | 2005 | United States | 4012828 | 79 |
| GCA_000237705.2 | 3546-06 | 2006 | United States | 4012129 | 85 |
| GCA_000237725.2 | 3554-08 | 2008 | United States | 4019938 | 80 |
| GCA_000237765.2 | 3582-05 | 2005 | United States | 3975631 | 67 |
| GCA_000786345.1 | 3265/80 | 2014 | Russia | 4012518 | 96 |
| GCA_000786335.1 | 81 | 2014 | Russia | 4011922 | 90 |
| GCA_000257415.2 | 2011EL-301 | 2011 | Russia | 4002872 | 163 |
| GCA_000939665.1 | MAK 97 | 1937 | Indonesia | 3894875 | 130 |
| GCA_000966375.1 | P18899-D | 2006 | Russia | 3996106 | 119 |
| GCA_000348365.1 | EM-1727 | 2011 | Bangladesh | 4067194 | 109 |
| GCA_000487955.1 | E306 | 2013 | China | 4165066 | 51 |
| GCA_000788415.1 | 2010AA-143 | 2010 | Haiti | 4027992 | 108 |
| GCA_000788425.1 | 2010AA-142 | 2010 | Haiti | 4026654 | 125 |
| GCA_000788435.1 | 2010AA-144 | 2010 | Haiti | 4026834 | 87 |
| GCA_000788495.1 | 2012Env-2 | 2012 | Haiti | 3997495 | 121 |
| GCA_000788535.1 | 2010AA-145 | 2010 | Haiti | 4017309 | 93 |
| GCA_000788555.1 | 2010AA-146 | 2010 | Haiti | 4028110 | 120 |
| GCA_000788575.1 | 2010AA-147 | 2010 | Haiti | 4027597 | 109 |
| GCA_000788595.1 | 2010AA-148 | 2010 | Haiti | 4027400 | 124 |
| GCA_000788615.1 | 2010AA-150 | 2010 | Haiti | 4025245 | 116 |
| GCA_000788635.1 | 2010AA-151 | 2010 | Haiti | 4028131 | 118 |
| GCA_000788655.1 | 2012Env-131 | 2012 | Haiti | 4028252 | 119 |
| GCA_000788675.1 | 2012Env-32 | 2012 | Haiti | 3966360 | 97 |
| GCA_000788695.1 | 2012Env-326 | 2012 | Haiti | 4060530 | 577 |
| GCA_000788735.1 | 2012Env-90 | 2012 | Haiti | 4012614 | 115 |
| GCA_000788755.1 | 2012Env-92 | 2012 | Haiti | 3893268 | 140 |
| GCA_000788775.1 | 2012HC-25 | 2012 | Haiti | 3965905 | 142 |
| GCA_000788795.1 | 2012HC-24 | 2012 | Haiti | 4017899 | 123 |
| GCA_000788815.1 | 2012HC-34 | 2012 | Haiti | 4014093 | 111 |
| GCA_000788835.1 | 2012HC-31 | 2012 | Haiti | 4012329 | 113 |
| GCA_000788855.1 | 2012Env-94 | 2012 | Haiti | 4014775 | 126 |
| GCA_000788875.1 | 2012HC-07 | 2012 | Haiti | 4080364 | 114 |
| GCA_000788895.1 | 2012HC-18 | 2012 | Haiti | 4024111 | 114 |
| GCA_000788915.1 | 2012HC-17 | 2012 | Haiti | 4012003 | 102 |
| GCA_000788935.1 | 2012HC-32 | 2012 | Haiti | 4014797 | 110 |
| GCA_000788955.1 | 2012HC-35 | 2012 | Haiti | 4012010 | 111 |
| GCA_000788975.1 | 2012HC-33 | 2012 | Haiti | 4014752 | 107 |
| GCA_000788995.1 | 2012HC-21 | 2012 | Haiti | 4026556 | 113 |
| GCA_000789015.1 | 2012HC-22 | 2012 | Haiti | 4015520 | 115 |
| GCA_000789035.1 | 2012HC-11 | 2012 | Haiti | 4029722 | 103 |
| GCA_000789055.1 | 2012HC-16 | 2012 | Haiti | 4058831 | 105 |
| GCA_000789075.1 | 2012HC-15 | 2012 | Haiti | 4048930 | 170 |
| GCA_000789095.1 | 2012HC-19 | 2012 | Haiti | 4010541 | 117 |
| GCA_000789115.1 | 2012HC-08 | 2012 | Haiti | 4078954 | 110 |
| GCA_000789135.1 | 2012HC-10 | 2012 | Haiti | 4012032 | 108 |
| GCA_000789155.1 | 2012HC-12 | 2012 | Haiti | 4022401 | 128 |
| GCA_000153865.1 | MAK 757 | 1937 | Indonesia | 3936003 | 12 |
| GCA_000500675.1 | RND19187 | 2010 | Russia | 4001659 | 203 |
| GCA_000500695.1 | RND18899 | 2006 | Russia | 3991231 | 164 |
| GCA_000500715.1 | RND6878 | 2012 | Russia | 4000976 | 178 |
| GCA_000500735.1 | RND18826 | 2005 | Russia | 4036141 | 246 |
| GCA_000174235.1 | VL426 | ND | United Kingdom | 3987383 | 5 |
| GCA_000174295.1 | TMA 21 | 1982 | Brazil | 4023772 | 20 |
| GCA_000174275.1 | RC9 | 1985 | Kenya | 4211011 | 11 |
| GCA_000174315.1 | B33 | 2004 | Mozambique | 4154698 | 17 |
| GCA_000174255.1 | TM 11079-80 | 1980 | Brazil | 4055140 | 35 |
| GCA_000812045.1 | ZWU0020 | ND | United States | 4038248 | 245 |
| GCA_001251435.1 | 7687 | 2009 | Kenya | 4030860 | 74 |
| GCA_001251495.1 | GP16 | 1971 | India | 3968603 | 62 |
| GCA_001251935.1 | 6194 | 2007 | Kenya | 4022708 | 53 |
| GCA_001252055.1 | 4551 | 2007 | India | 4032044 | 52 |
| GCA_001252075.1 | 4121 | 2004 | Vietnam | 3948118 | 47 |
| GCA_001045415.1 | TSY216 | 2010 | Thailand | 5000494 | 3 |
| GCA_000174115.1 | 12129(1) | 1985 | Australia | 3969506 | 12 |
| GCA_000174335.1 | BX 330286 | 1986 | Australia | 4000672 | 8 |
| GCA_000175695.1 | CIRS 101 | 2002 | Bangladesh | 4059686 | 18 |
| GCA_001252675.1 | V5 | 1989 | India | 3989146 | 58 |
| GCA_001248645.1 | 6214 | 2007 | Kenya | 4031671 | 127 |
| GCA_001247885.1 | IDHO1_726 | 2009 | India | 4015875 | 70 |
| GCA_001254535.1 | A59 | 1970 | India | 3990340 | 89 |
| GCA_001254095.1 | A325 | 1993 | Argentina | 3966042 | 29 |
| GCA_001250035.1 | GP145 | 1979 | India | 3946053 | 62 |
| GCA_001250435.1 | 4642 | 2006 | India | 3996103 | 156 |
| GCA_001250455.1 | A32 | 1991 | Peru | 4002262 | 50 |
| GCA_001254055.1 | A4 | 1973 | ND | 3946745 | 51 |
| GCA_001254335.1 | 4784 | 2009 | Tanzania | 4018257 | 41 |
| GCA_000152465.2 | V51 | 1987 | United States | 4208620 | 80 |
| GCA_001257215.1 | A330 | 1993 | India | 4039116 | 56 |
| GCA_001257975.1 | A383 | 2002 | Bangladesh | 3991423 | 57 |
| GCA_001258535.1 | 7686 | 2009 | Kenya | 4027393 | 99 |
| GCA_001259715.1 | 4538 | 2007 | India | 4022271 | 50 |
| GCA_001259995.1 | A215 | 1985 | United States | 3936701 | 37 |
| GCA_001260075.1 | GP143 | 1978 | Bahrain | 3988411 | 127 |
| GCA_001248505.1 | 4519 | 2005 | India | 4012798 | 50 |
| GCA_001256355.1 | PRL5 | 1980 | India | 3983009 | 55 |
| GCA_001258995.1 | 4646 | 2007 | India | 4045251 | 50 |
| GCA_001261135.1 | A245 | 1989 | Vietnam | 3982535 | 72 |
| GCA_001257035.1 | 4110 | 1995 | Vietnam | 3918727 | 97 |
| GCA_001259495.1 | A76 | 1982 | Bangladesh | 4016974 | 80 |
| GCA_001259795.1 | A389 | 1987 | Bangladesh | 4094216 | 116 |
| GCA_001256675.1 | A241 | 1989 | Vietnam | 4021050 | 62 |
| GCA_001252875.1 | A152 | 1991 | Mozambique | 3985453 | 71 |
| GCA_001247245.1 | 4679 | 1999 | Bangladesh | 4012494 | 68 |
| GCA_001259055.1 | 4113 | 2003 | Vietnam | 3925537 | 39 |
| GCA_001259475.1 | 6210 | 2007 | Kenya | 4035607 | 40 |
| GCA_001259875.1 | 4661 | 2001 | Bangladesh | 3922782 | 221 |
| GCA_001248465.1 | V212-1 | 1991 | India | 3945950 | 255 |
| GCA_001260995.1 | A27 | 1991 | Peru | 3996840 | 62 |
| GCA_001256015.1 | 4663 | 2001 | Bangladesh | 4010680 | 75 |
| GCA_001257835.1 | 6193 | 2005 | Kenya | 4032637 | 48 |
| GCA_001260175.1 | 4662 | 2001 | Bangladesh | 4012468 | 48 |
| GCA_001249315.1 | 4322 | 2004 | India | 3981548 | 123 |
| GCA_001261515.1 | A201 | 1992 | Argentina | 3979384 | 84 |
| GCA_001261535.1 | A487(1) | 2007 | Bangladesh | 4018875 | 49 |
| GCA_001261555.1 | 6201 | 2007 | Kenya | 4022982 | 56 |
| GCA_001257075.1 | A488(1) | 2006 | Bangladesh | 4022752 | 50 |
| GCA_001257255.1 | V109 | 1990 | India | 3995420 | 74 |
| GCA_001257895.1 | 4593 | 2007 | India | 4031342 | 57 |
| GCA_001259315.1 | A131 | 1989 | India | 4039161 | 89 |
| GCA_001259635.1 | A68 | 1949 | Egypt | 3977514 | 71 |
| GCA_001260295.1 | 1362 | 2005 | Mozambique | 4064557 | 40 |
| GCA_001260335.1 | 4339 | 2004 | India | 4019959 | 48 |
| GCA_001251975.1 | 6212 | 2007 | Kenya | 4040996 | 149 |
| GCA_001258495.1 | 4656 | 2006 | India | 4035639 | 98 |
| GCA_001258555.1 | 4488 | 2006 | India | 4012653 | 56 |
| GCA_001259135.1 | 4605 | 2007 | India | 4018974 | 92 |
| GCA_001259235.1 | 4552 | 2007 | India | 4033126 | 52 |
| GCA_001253055.1 | 4600 | 2007 | India | 4028076 | 51 |
| GCA_001259555.1 | A46 | 1964 | ND | 4007597 | 75 |
| GCA_001260915.1 | A66 | 1962 | Bangladesh | 4012864 | 97 |
| GCA_001261075.1 | PRL64 | 1992 | India | 4027134 | 54 |
| GCA_001261335.1 | GP60 | 1973 | India | 3871365 | 180 |
| GCA_001184775.1 | YN98296 | 1998 | China | 4004435 | 53 |
| GCA_001186485.1 | EM-1626 | 2011 | Bangladesh | 4058026 | 83 |
| GCA_001186495.1 | NHCC-078 | 2011 | Bangladesh | 4032908 | 128 |
| GCA_001186505.1 | EM-1652A | 2011 | Bangladesh | 4052192 | 68 |
| GCA_001186515.1 | EM-1543 | 2010 | Bangladesh | 4064052 | 98 |
| GCA_001186565.1 | EM-1688 | 2011 | Bangladesh | 4054925 | 131 |
| GCA_001186575.1 | EM-1654 | 2011 | Bangladesh | 4053299 | 85 |
| GCA_001186585.1 | EM-1690A | 2011 | Bangladesh | 3961642 | 95 |
| GCA_001186595.1 | EM-1690 | 2011 | Bangladesh | 4056072 | 78 |
| GCA_001186645.1 | EM-1706 | 2011 | Bangladesh | 3944903 | 88 |
| GCA_001186655.1 | NHCC-011 | 2010 | Bangladesh | 4034922 | 87 |
| GCA_001186665.1 | NHCC-04 | 2010 | Bangladesh | 4039258 | 131 |
| GCA_001186675.1 | NHCC-048 | 2010 | Bangladesh | 4041973 | 113 |
| GCA_001186725.1 | NHCC-021 | 2010 | Bangladesh | 4044257 | 101 |
| GCA_001186735.1 | NHCC-042 | 2010 | Bangladesh | 4040251 | 101 |
| GCA_001186755.1 | NHCC-019 | 2010 | Bangladesh | 4006456 | 249 |
| GCA_001186785.1 | NHCC-05 | 2010 | Bangladesh | 4063046 | 102 |
| GCA_001186805.1 | NHCC-081 | 2011 | Bangladesh | 4036135 | 91 |
| GCA_001186825.1 | NHCM-01 | 2010 | Bangladesh | 4058157 | 129 |
| GCA_001186835.1 | NHCC-083 | 2011 | Bangladesh | 4032941 | 87 |
| GCA_001186855.1 | NHCM-02 | 2011 | Bangladesh | 4058043 | 89 |
| GCA_001186885.1 | NHCM-06 | 2011 | Bangladesh | 4065293 | 152 |
| GCA_001186905.1 | NHCM-04 | 2011 | Bangladesh | 4051907 | 87 |
| GCA_001186915.1 | NHCM-012 | 2011 | Bangladesh | 4058997 | 87 |
| GCA_001186925.1 | NHCM-013 | 2011 | Bangladesh | 4056172 | 142 |
| GCA_001186965.1 | NHCM-016A | 2011 | Bangladesh | 4054882 | 88 |
| GCA_001186985.1 | NHCM-017 | 2011 | Bangladesh | 4270351 | 212 |
| GCA_001186995.1 | NHCM-029 | 2011 | Bangladesh | 4058323 | 86 |
| GCA_001187015.1 | NHCM-044 | 2011 | Bangladesh | 4031872 | 122 |
| GCA_001187025.1 | NHCM-037 | 2011 | Bangladesh | 4053555 | 95 |
| GCA_001187065.1 | NHCM-033 | 2011 | Bangladesh | 4091543 | 84 |
| GCA_001187085.1 | NHCM-043 | 2011 | Bangladesh | 4054814 | 172 |
| GCA_001187095.1 | NHCM-045 | 2011 | Bangladesh | 4036868 | 98 |
| GCA_001187105.1 | NHCM-053 | 2011 | Bangladesh | 4056015 | 78 |
| GCA_001187145.1 | NHCM-047 | 2011 | Bangladesh | 4041405 | 112 |
| GCA_001187165.1 | NHCM-054 | 2011 | Bangladesh | 4037409 | 79 |
| GCA_001187175.1 | NHCM-048 | 2011 | Bangladesh | 3956153 | 108 |
| GCA_001187185.1 | NHCC-068 | 2010 | Bangladesh | 4033814 | 112 |
| GCA_001187225.1 | NHCC-079 | 2011 | Bangladesh | 4037755 | 97 |
| GCA_001187245.1 | NHCC-080 | 2011 | Bangladesh | 4032145 | 105 |
| GCA_001187255.1 | EM-1542 | 2010 | Bangladesh | 4055638 | 128 |
| GCA_001187265.1 | NHCM-03 | 2011 | Bangladesh | 4061995 | 87 |
| GCA_001254895.1 | MG116025 | 1991 | Bangladesh | 4027317 | 61 |
| GCA_001248865.1 | A193 | 1992 | Bolivia | 4006062 | 77 |
| GCA_001248905.1 | A70 | 1969 | Bangladesh | 3981994 | 75 |
| GCA_001248945.1 | A213 | 1984 | Georgia | 3943920 | 79 |
| GCA_001249085.1 | MBRN14 | 2004 | India | 4014074 | 61 |
| GCA_001249515.1 | 6191 | 2005 | Kenya | 4031393 | 93 |
| GCA_001249715.1 | GP152 | 1979 | India | 3964217 | 75 |
| GCA_001249795.1 | 4672 | 2000 | Bangladesh | 3972315 | 199 |
| GCA_001249995.1 | A177 | 1992 | Colombia | 4009459 | 58 |
| GCA_001250195.1 | MJ1485 | 1994 | Bangladesh | 4112661 | 68 |
| GCA_001250615.1 | A488(2) | 2006 | Bangladesh | 4017885 | 67 |
| GCA_001250795.1 | MBN17 | 2004 | India | 4019692 | 64 |
| GCA_001250935.1 | A61 | 1970 | India | 3966297 | 99 |
| GCA_001252495.1 | A18 | 1977 | India | 4012358 | 71 |
| GCA_001252775.1 | 6215 | 2005 | Kenya | 4033005 | 46 |
| GCA_001252855.1 | 4111 | 2002 | Vietnam | 3870008 | 132 |
| GCA_001252895.1 | 4585 | 2007 | India | 4028120 | 53 |
| GCA_001253035.1 | 1346 | 2005 | Mozambique | 4069009 | 40 |
| GCA_001253155.1 | A154 | 1991 | Mozambique | 3982016 | 76 |
| GCA_001253235.1 | A29 | 1991 | Peru | 4000671 | 52 |
| GCA_001253295.1 | A185 | 1992 | Colombia | 4009761 | 85 |
| GCA_001253315.1 | GP140 | 1978 | Malaysia | 3919716 | 254 |
| GCA_001253455.1 | 4122 | 2007 | Vietnam | 3747369 | 766 |
| GCA_001253575.1 | GP8 | 1970 | India | 3951857 | 84 |
| GCA_001253695.1 | A31 | 1991 | Peru | 4002336 | 48 |
| GCA_001253835.1 | A49 | 1962 | ND | 4003129 | 263 |
| GCA_001254355.1 | MG116226 | 1991 | Bangladesh | 4028960 | 46 |
| GCA_001254435.1 | GP160 | 1980 | India | 3986399 | 52 |
| GCA_001254575.1 | A103 | 1990 | ND | 4019492 | 93 |
| GCA_001254635.1 | 4623 | 2007 | India | 4034153 | 48 |
| GCA_001254655.1 | A10 | 1979 | Bangladesh | 4145031 | 49 |
| GCA_001254675.1 | A5 | 1989 | Angola | 4032311 | 51 |
| GCA_001254815.1 | 4675 | 2001 | Bangladesh | 3985607 | 156 |
| GCA_001254955.1 | 6197 | 2007 | Kenya | 4030166 | 203 |
| GCA_001255155.1 | A22 | 1979 | Bangladesh | 3979233 | 52 |
| GCA_001255295.1 | A200 | 1992 | Argentina | 4005389 | 76 |
| GCA_001255575.1 | A6 | 1957 | Indonesia | 3998598 | 61 |
| GCA_001255835.1 | 4536 | 2007 | India | 4024336 | 54 |
| GCA_001255915.1 | 7685 | 2009 | Kenya | 4024204 | 84 |
| GCA_001247525.1 | A346(1) | 1994 | Bangladesh | 4095237 | 46 |
| GCA_001247835.1 | 1627 | 2005 | Mozambique | 4065829 | 36 |
| GCA_001248135.1 | A186 | 1992 | Argentina | 4004225 | 81 |
| GCA_001248195.1 | A60 | 1958 | Thailand | 3997697 | 120 |
| GCA_000153785.3 | AM-19226 | ND | ND | 4053126 | 33 |
| GCA_000153985.3 | MZO-2 | ND | ND | 3977246 | 36 |
| GCA_000154005.2 | 623-39 | 2002 | Bangladesh | 4164181 | 39 |
| GCA_001597715.1 | I-1181 | 1994 | Russia | 2920335 | 28 |
| GCA_001617665.1 | CW-6 | 1966 | India | 3921611 | 91 |
| GCA_001617675.1 | M888D | 1970 | Russia | 3970532 | 124 |
| GCA_000167935.2 | V52 | ND | Sudan | 4045303 | 59 |
| GCA_000305645.2 | HC-55B2 | 2010 | Haiti | 3934012 | 109 |
| GCA_000168895.2 | 1587 | 1587 | Peru | 4216194 | 73 |
| GCA_000168935.3 | MZO-3 | ND | ND | 4137330 | 45 |
| GCA_000220745.3 | HCUF01 | ND | ND | 4067550 | 22 |
| GCA_000220765.3 | HE39 | ND | ND | 3937798 | 16 |
| GCA_000234455.3 | HC-61A1 | ND | ND | 4067936 | 21 |
| GCA_000237745.2 | 3569-08 | 2008 | United States | 3958670 | 81 |
| GCA_000299495.2 | VC35 | 2004 | Malaysia | 3912714 | 103 |
| GCA_000299515.2 | VC1761 | 2009 | Malaysia | 4011457 | 149 |
| GCA_000299535.2 | VC4370 | 2008 | Malaysia | 3986677 | 145 |
| GCA_000569115.2 | PCS-022 | ND | ND | 4055383 | 105 |
| GCA_000304915.2 | CP1035(8) | 2004 | Mexico | 3933356 | 158 |
| GCA_000304935.2 | HC-17A1 | 2010 | Haiti | 4174355 | 405 |
| GCA_000304955.2 | HC-41B1 | 2010 | Haiti | 3883402 | 121 |
| GCA_000305015.2 | HC-55C2 | 2010 | Haiti | 3940186 | 125 |
| GCA_000305055.2 | HC-60A1 | 2010 | Haiti | 3938002 | 132 |
| GCA_000305075.2 | HC-62A1 | 2010 | Haiti | 4032880 | 105 |
| GCA_000305095.2 | HC-77A1 | 2010 | Haiti | 4040127 | 112 |
| GCA_000305115.2 | HE-40 | 2010 | Haiti | 3875898 | 102 |
| GCA_000305135.2 | HE-46 | 2011 | Haiti | 3874516 | 100 |
| GCA_000305195.2 | HC-59A1 | 2010 | Haiti | 3934654 | 114 |
| GCA_000305525.2 | HC-02C1 | 2010 | Haiti | 3935576 | 121 |
| GCA_000305545.2 | HC-59B1 | 2010 | Haiti | 3934504 | 107 |
| GCA_000305565.2 | HC-44C1 | 2010 | Haiti | 3876537 | 90 |
| GCA_000305585.2 | HC-37A1 | 2010 | Haiti | 4029550 | 97 |
| GCA_000305625.2 | HC-62B1 | 2010 | Haiti | 4028528 | 71 |
| GCA_000305675.2 | HC-17A2 | 2010 | Haiti | 4023476 | 94 |
| GCA_000305695.2 | HC-69A1 | 2010 | Haiti | 4024183 | 93 |
| GCA_001402255.1 | YB4G06 | 2009 | United States | 4032120 | 65 |
| GCA_001402185.1 | YB1A01 | 2009 | United States | 3876619 | 56 |
| GCA_001402265.1 | YB4F05 | 2009 | United States | 3886413 | 50 |
| GCA_001402275.1 | YB3G04 | 2009 | United States | 4031104 | 68 |
| GCA_001402335.1 | YB7A06 | 2009 | United States | 3881934 | 58 |
| GCA_001402285.1 | YB4C07 | 2009 | United States | 4015430 | 44 |
| GCA_001402365.1 | YB1G06 | 2009 | United States | 3938799 | 49 |
| GCA_001402375.1 | YB2A06 | 2009 | United States | 4033481 | 63 |
| GCA_001402415.1 | YB2G05 | 2009 | United States | 3888413 | 51 |
| GCA_001402425.1 | YB2G07 | 2009 | United States | 3941216 | 61 |
| GCA_001402435.1 | YB5A06 | 2009 | United States | 3886853 | 57 |
| GCA_001402445.1 | YB6A06 | 2009 | United States | 3884851 | 51 |
| GCA_001402535.1 | YB2A05 | 2009 | United States | 3886789 | 52 |
| GCA_001402545.1 | YB3B05 | 2009 | United States | 4014368 | 47 |
| GCA_001402575.1 | YB4G05 | 2009 | United States | 3927241 | 54 |
| GCA_001402585.1 | YB4H02 | 2009 | United States | 4039090 | 67 |
| GCA_001402595.1 | YB7A09 | 2009 | United States | 3885495 | 49 |
| GCA_001402605.1 | YB4B03 | 2009 | United States | 3916598 | 62 |
| GCA_001402655.1 | YB8E08 | 2009 | United States | 4013838 | 44 |
| GCA_001402745.1 | 877-163 | 2002 | Bangladesh | 3864837 | 278 |
| GCA_001411585.1 | YB2G01 | 2009 | United States | 4032885 | 64 |
| GCA_000338215.2 | P-18785 | 2005 | Russia | 3978895 | 158 |
| GCA_000348045.2 | 116059 | 1992 | Brazil | 4017055 | 92 |
| GCA_000348065.2 | 116063 | 1978 | Brazil | 3970379 | 144 |
| GCA_000348085.2 | 87395 | 1983 | Mexico | 3858111 | 180 |
| GCA_000348105.2 | 95412 | 1987 | Mexico | 4030154 | 103 |
| GCA_000348125.2 | AG-7404 | 1991 | Bangladesh | 3915123 | 95 |
| GCA_000348145.2 | AG-8040 | 1991 | Bangladesh | 3977194 | 103 |
| GCA_000348165.2 | EC-0009 | 2010 | Bangladesh | 4028271 | 87 |
| GCA_000348185.2 | EC-0012 | 2010 | Bangladesh | 4031923 | 96 |
| GCA_000348205.2 | EC-0027 | 2011 | Bangladesh | 4027674 | 94 |
| GCA_000348225.2 | EC-0051 | 2011 | Bangladesh | 4069374 | 99 |
| GCA_000348245.2 | EDC-020 | 2010 | Bangladesh | 4028191 | 85 |
| GCA_000348265.2 | EDC-022 | 2010 | Bangladesh | 4050458 | 106 |
| GCA_000348305.2 | EM-1546 | 2010 | Bangladesh | 4061605 | 90 |
| GCA_000348285.2 | EM-1536 | 2010 | Bangladesh | 4053577 | 100 |
| GCA_000348345.2 | EM-1676A | 2011 | Bangladesh | 4007225 | 85 |
| GCA_000348385.2 | NHCC-004A | 2010 | Bangladesh | 4031643 | 86 |
| GCA_000348405.2 | NHCC-006C | 2010 | Bangladesh | 4028239 | 86 |
| GCA_000348425.2 | NHCC-008D | 2010 | Bangladesh | 3971576 | 81 |
| GCA_000348445.2 | NHCC-010F | 2010 | Bangladesh | 4028811 | 92 |
| GCA_000348465.2 | Nep-21106 | 2003 | Nepal | 4031227 | 80 |
| GCA_000348485.2 | Nep-21113 | 2003 | Nepal | 4042420 | 100 |
| GCA_000348505.2 | PCS-023 | 2010 | Bangladesh | 4031594 | 86 |
| GCA_001281585.1 | 31 | 2011 | Ukraine | 4041947 | 95 |
| GCA_001281595.1 | 39 | 2011 | Ukraine | 4024490 | 96 |
| GCA_001281615.1 | 43 | 1994 | Ukraine | 4181484 | 204 |
| GCA_001281665.1 | 56 | 1994 | Ukraine | 4105143 | 95 |
| GCA_001282605.1 | EC-051 | 2010 | Bangladesh | 4068854 | 112 |
| GCA_001292745.1 | 116-17b | ND | ND | 4125773 | 186 |
| GCA_001292785.1 | BRV8 | ND | United Kingdom | 4109032 | 152 |
| GCA_001515085.1 | M1399 | 1982 | Russia | 4013390 | 127 |
| GCA_001515105.1 | M1395 | 1981 | Russia | 4028990 | 182 |
| GCA_001515115.1 | M988 | 1972 | Turkmenistan | 3995831 | 123 |
| GCA_001517845.1 | M1275 | 1993 | Russia | 4124381 | 106 |
| GCA_001318185.1 | KW3 | ND | ND | 4089020 | 2 |
| GCA_001521835.1 | M888 | 1970 | Russia | 4041101 | 254 |
| GCA_001543465.1 | PIC018 | 2007 | Bangladesh | 4029307 | 154 |
| GCA_001543505.1 | Drakes2013 | 2013 | United States | 4042766 | 156 |
| GCA_001515165.1 | M1522 | 2014 | Russia | 3955145 | 91 |
| GCA_001661905.1 | I-1187 | 1994 | Russia | 3957421 | 51 |
| GCA_001683415.1 | 2740-80 | 1980 | United States | 4088961 | 2 |
| GCA_001637545.1 | M139 | 1965 | Turkmenistan | 3972882 | 143 |
| GCA_001637555.1 | M299 | 1965 | Turkmenistan | 3957801 | 153 |
| GCA_001637575.1 | M1501 | 2011 | Russia | 4011026 | 156 |
| GCA_001639085.1 | P13762 | 1988 | Uzbekistan | 4096073 | 116 |
| GCA_001641685.1 | M1518 | 2012 | Russia | 4014173 | 133 |
| GCA_001641705.1 | M1524 | 2013 | Russia | 3961165 | 128 |
| GCA_001641745.1 | 6/67 | 1967 | India | 4006954 | 182 |
| GCA_001641765.1 | M1327 | 1998 | Russia | 4119805 | 89 |
| GCA_001887515.1 | NCTC 5395 | 1938 | Iraq | 4170245 | 2 |
| GCA_001887615.1 | NCTC 9420 | 1954 | Egypt | 4076583 | 2 |
| GCA_000788715.2 | 2012Env-9 | 2012 | Haiti | 4061813 | 2 |
| GCA_001854425.1 | Env-390 | 2012 | Haiti | 4050927 | 2 |
| GCA_001887395.1 | C5 | 1957 | Indonesia | 4102038 | 2 |
| GCA_001887415.1 | E1320 | 1974 | China | 4110440 | 2 |
| GCA_001887435.1 | CRC711 | 1964 | India | 4057520 | 2 |
| GCA_001887455.1 | CRC1106 | 1962 | India | 4099119 | 2 |
| GCA_001887495.1 | E1162 | 1962 | China | 4110872 | 2 |
| GCA_001887635.1 | M2140 | 1977 | Australia | 4014863 | 2 |
| GCA_001887655.1 | E9120 | 1961 | Indonesia | 4066727 | 2 |
| GCA_001887475.1 | E506 | 1974 | United States | 4062508 | 2 |
| GCA_001718095.1 | L15 | 2006 | Sweden | 4083945 | 49 |
| GCA_001729125.1 | 857 | 1996 | Bangladesh | 3993682 | 106 |
| GCA_001729195.1 | VC22 | 1981 | United States | 4022142 | 113 |
| GCA_001743085.1 | 3223-74 | 1974 | Guam | 4017920 | 160 |
| GCA_001857145.1 | 2559-78 | 1978 | United States | 3982589 | 148 |
| GCA_001857155.1 | VC53 | 2009 | United States | 4217958 | 247 |
| GCA_001857165.1 | VC48 | 1981 | United States | 3931795 | 152 |
| GCA_001857175.1 | VC56 | 2009 | United States | 4193045 | 216 |
| GCA_001857225.1 | 2631-78 | 1978 | United States | 3943436 | 151 |
| GCA_001857245.1 | 2512-86 | 1986 | United States | 3999880 | 175 |
| GCA_001857265.1 | 3272-78 | 1977 | United States | 3946798 | 130 |
| GCA_001857285.1 | 692-79 | 1979 | United States | 3938077 | 104 |
| GCA_001857305.1 | 2479-86 | 1986 | United States | 4011444 | 152 |
| GCA_001857325.1 | 1496-86 | 1986 | United States | 3972280 | 148 |
| GCA_001857345.1 | 2523-87 | 1974 | United States | 3947558 | 162 |
| GCA_001857365.1 | 3225-74 | 1974 | Guam | 4002038 | 165 |
| GCA_001857425.1 | 2633-78 | 1978 | Brazil | 3902363 | 121 |
| GCA_001857405.1 | 1074-78 | 1978 | Brazil | 3967821 | 146 |
| GCA_001857435.1 | C6706 | 1991 | Peru | 4019194 | 132 |
| GCA_001857455.1 | SIO | 2000 | United States | 3998588 | 150 |
| GCA_001857485.1 | TP | 2000 | United States | 4060799 | 291 |
| GCA_001857505.1 | 3568-07 | 2007 | Mexico | 4056633 | 142 |
| GCA_001857515.1 | HE46 | 2011 | Haiti | 3978873 | 143 |
| GCA_001718105.1 | L11 | 2006 | Sweden | 4000957 | 29 |
| GCA_001735565.1 | S12 | 2009 | Australia | 4061577 | 83 |
| GCA_001858445.1 | CMR020 | ND | Cameroon | 4034563 | 599 |
| GCA_001858455.1 | CMR021 | 2011 | Cameroon | 4037271 | 189 |
| GCA_001858465.1 | CMR022 | 2011 | Cameroon | 4031968 | 173 |
| GCA_001858475.1 | CMR019 | ND | Cameroon | 4028526 | 173 |
| GCA_001860225.1 | CMR001 | 2010 | Cameroon | 4022419 | 300 |
| GCA_001858585.1 | CMR004 | 2010 | Cameroon | 4021213 | 579 |
| GCA_001860265.1 | CMR007 | 2010 | Cameroon | 4026765 | 261 |
| GCA_001860285.1 | CMR008 | 2010 | Cameroon | 4032975 | 195 |
| GCA_001860295.1 | CMR009 | 2010 | Cameroon | 4029303 | 204 |
| GCA_001860315.1 | CMR010 | 2010 | Cameroon | 4035994 | 377 |
| GCA_001860345.1 | CMR011 | 2011 | Cameroon | 4024152 | 688 |
| GCA_001860365.1 | CMR012 | 2011 | Cameroon | 4027803 | 199 |
| GCA_001860385.1 | CMR013 | 2011 | Cameroon | 4034667 | 161 |
| GCA_001860395.1 | CMR014 | 2011 | Cameroon | 4042618 | 467 |
| GCA_001860425.1 | CMR015 | 2011 | Cameroon | 4027621 | 270 |
| GCA_001860445.1 | CMR016 | 2011 | Cameroon | 4026175 | 300 |
| GCA_001860465.1 | CMR017 | 2011 | Cameroon | 4034135 | 149 |
| GCA_001860485.1 | CMR018 | ND | Cameroon | 4036389 | 583 |
| GCA_001899465.1 | 76 | 2011 | Ukraine | 4000689 | 265 |
| GCA_001953365.1 | DL4211 | 2008 | United States | 3985387 | 416 |
| GCA_001953375.1 | DL4215 | 2008 | United States | 3981208 | 78 |
| GCA_002078825.1 | 17609 | 2015 | Tanzania | 4045510 | 99 |
| GCA_002078815.1 | 20390 | 2015 | Tanzania | 4045740 | 95 |
| GCA_002078635.1 | 39361 | 2015 | Tanzania | 4024330 | 93 |
| GCA_002078755.1 | TEM/29/01-003 | 2012 | Tanzania | 4041320 | 99 |
| GCA_002078595.1 | 43Ki | 2015 | Tanzania | 4048514 | 98 |
| GCA_002078795.1 | J8YRS KAGUNGA | 2015 | Tanzania | 4044226 | 96 |
| GCA_002078715.1 | 19886 | 2015 | Tanzania | 4039107 | 96 |
| GCA_002078705.1 | 21027 | 2015 | Tanzania | 4043733 | 92 |
| GCA_002078695.1 | TEM/15/01-005 | 2012 | Tanzania | 4043231 | 106 |
| GCA_002078055.1 | 36KI | 2015 | Tanzania | 4020592 | 57 |
| GCA_002076155.1 | O1S | 2015 | Tanzania | 4024114 | 101 |
| GCA_002076165.1 | O7S | 2015 | Tanzania | 4023827 | 96 |
| GCA_002076235.1 | TEM/25/01-004 | 2012 | Tanzania | 4042340 | 102 |
| GCA_002076245.1 | O9S | 2015 | Tanzania | 4022009 | 94 |
| GCA_002076255.1 | O2 | 2015 | Tanzania | 4044276 | 108 |
| GCA_002076455.1 | OO4 | 2015 | Tanzania | 4025417 | 99 |
| GCA_002076485.1 | 47623 | 2015 | Tanzania | 4022899 | 103 |
| GCA_002076645.1 | 47610 | 2015 | Tanzania | 4022193 | 99 |
| GCA_002076695.1 | 8Mo | 2015 | Tanzania | 4023189 | 108 |
| GCA_002097735.1 | CISM_300055 | 2008 | Mozambique | 4148676 | 194 |
| GCA_002097745.1 | CISM_300205 | 2008 | Mozambique | 3937137 | 249 |
| GCA_002097755.1 | CISM_300506 | 2008 | Mozambique | 4026639 | 112 |
| GCA_002097765.1 | CISM_S/Nida | ND | Mozambique | 4195572 | 113 |
| GCA_002097815.1 | CISM_1163068.5 | 2012 | Mozambique | 4014933 | 151 |
| GCA_002097825.1 | CISM_780298.0 | ND | Mozambique | 4194523 | 112 |
| GCA_002097835.1 | CISM_770180.8 | ND | Mozambique | 4190491 | 96 |
| GCA_002097845.1 | CISM_770067.4 | ND | Mozambique | 4193261 | 111 |
| GCA_002097895.1 | CISM_710180.8 | ND | Mozambique | 4193899 | 93 |
| GCA_002097905.1 | CISM_740115.4 | ND | Mozambique | 4034260 | 89 |
| GCA_002097925.1 | CISM_655665.0 | ND | Mozambique | 4037096 | 95 |
| GCA_002097915.1 | CISM_769845.7 | ND | Mozambique | 4195639 | 101 |
| GCA_002097975.1 | CISM_655630.3 | ND | Mozambique | 4061319 | 151 |
| GCA_002097985.1 | CISM_511 | ND | Mozambique | 4034679 | 110 |
| GCA_002097995.1 | CISM_655664.3 | ND | Mozambique | 4167720 | 125 |
| GCA_002098005.1 | CISM_510 | ND | Mozambique | 4038804 | 98 |
| GCA_002098055.1 | CISM_420 | ND | Mozambique | 4029921 | 118 |
| GCA_002098065.1 | CISM_505 | ND | Mozambique | 4038667 | 93 |
| GCA_002098075.1 | CISM_399 | ND | Mozambique | 4036149 | 83 |
| GCA_002098085.1 | CISM_398 | ND | Mozambique | 4079517 | 319 |
| GCA_002098135.1 | CISM_382 | ND | Mozambique | 4038086 | 109 |
| GCA_002098145.1 | CISM_375 | ND | Mozambique | 4031104 | 126 |
| GCA_002098155.1 | CISM_374 | ND | Mozambique | 4040267 | 120 |
| GCA_002098195.1 | CISM_347 | ND | Mozambique | 4049584 | 129 |
| GCA_002098215.1 | CISM_326 | ND | Mozambique | 4163534 | 108 |
| GCA_002098225.1 | CISM_302015 | 2009 | Mozambique | 4207521 | 106 |
| GCA_002098235.1 | CISM_302029 | 2009 | Mozambique | 4221472 | 154 |
| GCA_002098295.1 | CISM_300043 | 2008 | Mozambique | 4038332 | 108 |
| GCA_002098255.1 | CISM_300215 | 2008 | Mozambique | 4065320 | 145 |
| GCA_002098305.1 | CISM_296 | ND | Mozambique | 4030126 | 130 |
| GCA_002098335.1 | CISM_300209 | 2008 | Mozambique | 4036397 | 90 |
| GCA_002098345.1 | CISM_300208 | 2008 | Mozambique | 4039049 | 100 |
| GCA_002098355.1 | CISM_196 | 2003 | Mozambique | 4195851 | 105 |
| GCA_002098365.1 | CISM_191 | 2003 | Mozambique | 4193176 | 99 |
| GCA_002098415.1 | CISM_188 | 2003 | Mozambique | 4196807 | 112 |
| GCA_002098425.1 | CISM_189 | 2003 | Mozambique | 4185376 | 154 |
| GCA_002098435.1 | CISM_179 | 2003 | Mozambique | 4194264 | 113 |
| GCA_002098445.1 | CISM_178 | 2003 | Mozambique | 4193944 | 114 |
| GCA_002098495.1 | CISM_152 | 2003 | Mozambique | 4035506 | 96 |
| GCA_002098515.1 | CISM_154 | 2003 | Mozambique | 4032160 | 101 |
| GCA_002098525.1 | CISM_151 | 2003 | Mozambique | 4037351 | 109 |
| GCA_002098535.1 | CISM_153 | 2003 | Mozambique | 4162416 | 105 |
| GCA_002098555.1 | CISM_147 | 2003 | Mozambique | 4196567 | 118 |
| GCA_002098595.1 | CISM_146 | 2003 | Mozambique | 4194205 | 95 |
| GCA_002098625.1 | CISM_134 | 2003 | Mozambique | 4194365 | 92 |
| GCA_002098605.1 | CISM_122 | 2003 | Mozambique | 4032037 | 108 |
| GCA_002098655.1 | CISM_121 | 2003 | Mozambique | 4034073 | 101 |
| GCA_002098675.1 | CISM_120 | 2003 | Mozambique | 4036655 | 100 |
| GCA_002098695.1 | CISM_105 | 2003 | Mozambique | 4195830 | 124 |
| GCA_002098705.1 | CISM_1020234.0 | 2010 | Mozambique | 4037201 | 98 |
| GCA_002098715.1 | CISM_1020231.9 | 2010 | Mozambique | 4026090 | 126 |
| GCA_002098755.1 | CISM_1020229.6 | 2010 | Mozambique | 4034980 | 90 |
| GCA_002098765.1 | CISM_101 | 2003 | Mozambique | 4034875 | 108 |
| GCA_002098795.1 | CISM_1019829.2 | 2010 | Mozambique | 4031863 | 121 |
| GCA_002098805.1 | CISM_1019828.5 | 2010 | Mozambique | 4036202 | 93 |
| GCA_002098835.1 | CISM_100 | 2003 | Mozambique | 4040221 | 105 |
| GCA_002098885.1 | CISM_0091 | 2003 | Mozambique | 4036548 | 95 |
| GCA_002098875.1 | CISM_0079 | 2003 | Mozambique | 4040112 | 110 |
| GCA_002098845.1 | CISM_091 | ND | Mozambique | 4162346 | 112 |
| GCA_002098915.1 | CISM_0074 | 2003 | Mozambique | 4035667 | 95 |
| GCA_002098935.1 | CISM_0035 | 2002 | Mozambique | 4034640 | 85 |
| GCA_002098955.1 | CISM_0034 | 2002 | Mozambique | 4036351 | 124 |
| GCA_002098965.1 | CISM_0019 | 2002 | Mozambique | 4033489 | 109 |
| GCA_002098995.1 | CISM_0018 | 2002 | Mozambique | 4036015 | 111 |
| GCA_002099015.1 | CISM_0017 | 2002 | Mozambique | 4036556 | 107 |
| GCA_002099035.1 | CISM_0016 | 2002 | Mozambique | 4032775 | 101 |
| GCA_002099055.1 | CISM_0015 | 2002 | Mozambique | 4038651 | 103 |
| GCA_002099065.1 | CISM_0014 | 2002 | Mozambique | 4032716 | 118 |
| GCA_002099095.1 | CISM_0010 | 2002 | Mozambique | 4157755 | 120 |
| GCA_002099115.1 | CISM_0008 | 2005 | Mozambique | 4035087 | 94 |
| GCA_002099125.1 | CISM_0005 | 2002 | Mozambique | 4034250 | 101 |
| GCA_002102575.1 | G_33 | 1986 | Guinea | 3933642 | 117 |
| GCA_002114205.1 | 617 | ND | Ukraine | 3960877 | 117 |
| GCA_002076185.1 | 11S | 2015 | Tanzania | 4025935 | 99 |
| GCA_002076175.1 | O6MU | 2015 | Tanzania | 4025207 | 104 |
| GCA_002076265.1 | TEM/10/01-002 | 2012 | Tanzania | 4044593 | 93 |
| GCA_002076315.1 | 48055 | 2015 | Tanzania | 4024390 | 90 |
| GCA_002076415.1 | 7714 | 2015 | Tanzania | 4022767 | 108 |
| GCA_002076425.1 | 1Mo | 2015 | Tanzania | 4024350 | 98 |
| GCA_002076465.1 | O3MU | 2015 | Tanzania | 4025781 | 107 |
| GCA_002076475.1 | 39Ki | 2015 | Tanzania | 4048610 | 119 |
| GCA_002076535.1 | 31Ki | 2015 | Tanzania | 4044237 | 113 |
| GCA_002076545.1 | O7MU | 2015 | Tanzania | 4025010 | 96 |
| GCA_002076575.1 | O3S | 2015 | Tanzania | 4022861 | 102 |
| GCA_002076585.1 | O5MU | 2015 | Tanzania | 4023442 | 102 |
| GCA_002076615.1 | 7Mo | 2015 | Tanzania | 4022979 | 95 |
| GCA_002076635.1 | 9Mo | 2015 | Tanzania | 4024326 | 102 |
| GCA_002076665.1 | 5Mo | 2015 | Tanzania | 4023405 | 99 |
| GCA_002076705.1 | 2Mo | 2015 | Tanzania | 4024773 | 104 |
| GCA_002076735.1 | TEM/12/12-001 | 2011 | Tanzania | 4041711 | 102 |
| GCA_002076745.1 | TEM/04/01-001 | 2012 | Tanzania | 4042748 | 110 |
| GCA_002076775.1 | 21B | 2015 | Tanzania | 4045625 | 98 |
| GCA_002076785.1 | 20478 | 2015 | Tanzania | 4046809 | 95 |
| GCA_002216685.1 | 2521-89 | 1989 | United States | 4123863 | 2 |
| GCA_002194155.1 | FC1877 | 1995 | India | 3984202 | 85 |
| GCA_002194165.1 | FC3611a | 1999 | India | 4013511 | 103 |
| GCA_002194185.1 | FC3611b | 1997 | India | 4013713 | 93 |
| GCA_002194215.1 | FC2273 | 1998 | India | 4022199 | 98 |
| GCA_002194245.1 | FC1384 | 2000 | India | 4014742 | 93 |
| GCA_002194235.1 | FC2271 | 1997 | India | 4016919 | 85 |
| GCA_002194265.1 | FC1341 | 2002 | India | 3999105 | 81 |
| GCA_002194295.1 | FC1105 | 2003 | India | 3960610 | 110 |
| GCA_002194305.1 | FC1817 | 1994 | India | 4043413 | 78 |
| GCA_002194335.1 | FC1225 | 2001 | India | 4003412 | 97 |
| GCA_002196055.1 | P-18748 | 2004 | Russia | 3961379 | 159 |
| GCA_002196065.1 | P-18778 | 2005 | Russia | 4012469 | 121 |
| GCA_002196095.1 | 102 | 2016 | Ukraine | 3995638 | 418 |
| GCA_002196105.1 | 147 | 2016 | Ukraine | 4107181 | 259 |
| GCA_002196135.1 | 89 | 2016 | Ukraine | 4115283 | 165 |
| GCA_002196155.1 | 155 | ND | Ukraine | 4117933 | 104 |
| GCA_002196175.1 | 28 | 2016 | Ukraine | 4076049 | 98 |
| GCA_002196165.1 | 5 | 2016 | Ukraine | 3960426 | 231 |
| GCA_002196225.1 | 114 | 2016 | Ukraine | 3971369 | 72 |
| GCA_002196255.1 | 85 | ND | Ukraine | 3979842 | 400 |
| GCA_002196305.1 | 433 | ND | Russia | 3984043 | 126 |
| GCA_002196275.1 | M1344 | ND | Russia | 4029820 | 117 |
| GCA_002196335.1 | M1030 | ND | Turkmenistan | 3951787 | 139 |
| GCA_002196375.1 | M1337 | ND | Russia | 4005292 | 271 |
| GCA_002196395.1 | 34Kayum | ND | Afghanistan | 3985205 | 142 |
| GCA_002204075.1 | 39 | 2011 | Ukraine | 3866753 | 972 |
| GCA_002204085.1 | 866 | 1996 | Ukraine | 3892514 | 373 |
| GCA_002204095.1 | 153 | 2011 | Ukraine | 4017602 | 181 |
| GCA_002204105.1 | 56 | 1995 | Ukraine | 3950861 | 516 |
| GCA_002217575.1 | W4-13 | 2013 | India | 3997832 | 140 |
| GCA_002251495.1 | NMH2016 | 2016 | United States | 3754606 | 125 |
| GCA_002284495.1 | OYP1G01 | 2009 | United States | 3969671 | 121 |
| GCA_002284425.1 | OYP2D07 | 2009 | United States | 4082264 | 135 |
| GCA_002284365.1 | OYP4B01 | 2009 | United States | 3929714 | 90 |
| GCA_002284475.1 | OYP2C05 | 2009 | United States | 3989036 | 50 |
| GCA_002284315.1 | OYP4H06 | 2009 | United States | 3907548 | 91 |
| GCA_002284255.1 | OYP4H08 | 2009 | United States | 3869161 | 448 |
| GCA_002284245.1 | OYP4H11 | 2009 | United States | 3934959 | 57 |
| GCA_002284175.1 | OYP6F08 | 2009 | United States | 3912172 | 66 |
| GCA_002284205.1 | OYP6D06 | 2009 | United States | 4036442 | 161 |
| GCA_002284075.1 | OYP7C09 | 2009 | United States | 3869397 | 75 |
| GCA_002284155.1 | OYP6G08 | 2009 | United States | 3950949 | 75 |
| GCA_002284455.1 | OYP1E07 | 2009 | United States | 3942520 | 199 |
| GCA_002284415.1 | OYP2E01 | 2009 | United States | 3966741 | 55 |
| GCA_002284395.1 | OYP2A12 | 2009 | United States | 4068380 | 68 |
| GCA_002284325.1 | OYP4G08 | 2009 | United States | 3927912 | 76 |
| GCA_002284265.1 | OYP6F10 | 2009 | United States | 3860908 | 87 |
| GCA_002284185.1 | OYP6E07 | 2009 | United States | 3957612 | 91 |
| GCA_002284125.1 | OYP8A01 | 2009 | United States | 3926089 | 185 |
| GCA_002284115.1 | OYP8F12 | 2009 | United States | 4038901 | 66 |
| GCA_002284355.1 | OYP3F10 | 2009 | United States | 3937113 | 117 |
| GCA_002284235.1 | OYP5F10 | 2009 | United States | 4088901 | 172 |
| GCA_002284095.1 | OYP8C06 | 2009 | United States | 4033034 | 72 |
| GCA_002313005.1 | ICDC-VC661 | 2006 | China | 4264715 | 3 |
| GCA_002313025.1 | FORC_055 | 2014 | South Korea | 4095733 | 2 |
| GCA_002407455.1 | VC0101557 | 2001 | South Korea | 3863050 | 54 |
| GCA_001250235.2 | A19 | 1971 | ND | 4033501 | 2 |
| GCA_001471585.2 | FDAARGOS_103 | ND | Germany | 4036048 | 2 |
| GCA_001471455.2 | ATCC 11629 | ND | ND | 4216088 | 7 |
| GCA_001525525.2 | FDAARGOS_102 | 1963 | India | 4071484 | 3 |
| GCA_002073335.2 | FDAARGOS_223 | ND | United States | 4042269 | 2 |
| GCA_002196295.1 | 2403 | ND | Ukraine | 4745993 | 2147 |
| GCA_002807705.1 | 330590 | 2014 | Bangladesh | 4041648 | 82 |
| GCA_002807725.1 | S002502 | 2013 | Bangladesh | 3946275 | 77 |
| GCA_002807805.1 | S003806 | 2014 | Bangladesh | 4046403 | 84 |
| GCA_002807985.1 | S002300_E | 2013 | Bangladesh | 4042300 | 77 |
| GCA_002808075.1 | S000600_C10 | 2013 | Bangladesh | 4041449 | 82 |
| GCA_002808215.1 | 22043300_C6 | 2013 | Bangladesh | 4042333 | 80 |
| GCA_002808265.1 | 330033_C1 | 2013 | Bangladesh | 4043006 | 81 |
| GCA_002808365.1 | S000100_C5 | 2013 | Bangladesh | 4075565 | 84 |
| GCA_002808305.1 | S042408 | 2014 | Bangladesh | 3999339 | 96 |
| GCA_002808405.1 | S081300_C2 | 2013 | Bangladesh | 4042531 | 83 |
| GCA_002808415.1 | 331721_C1 | 2013 | Bangladesh | 4042872 | 85 |
| GCA_002808435.1 | 22043204_C1 | 2013 | Bangladesh | 4043072 | 77 |
| GCA_002808485.1 | 330013_C1 | 2013 | Bangladesh | 4041908 | 80 |
| GCA_002807735.1 | S002506 | 2013 | Bangladesh | 4042063 | 81 |
| GCA_002807765.1 | 330073_A | 2013 | Bangladesh | 4042676 | 77 |
| GCA_002807785.1 | 330113 | 2014 | Bangladesh | 4042653 | 83 |
| GCA_002807825.1 | 220076-6 | 2014 | Bangladesh | 4043584 | 79 |
| GCA_002807835.1 | S002300_B | 2013 | Bangladesh | 3949769 | 75 |
| GCA_002807865.1 | 220075-6 | 2014 | Bangladesh | 3954018 | 80 |
| GCA_002807875.1 | 330920_B | 2013 | Bangladesh | 4044638 | 82 |
| GCA_002807895.1 | 330110 | 2014 | Bangladesh | 4043457 | 79 |
| GCA_002807925.1 | S023208 | 2014 | Bangladesh | 4027340 | 82 |
| GCA_002807945.1 | 330920_A | 2013 | Bangladesh | 4042529 | 83 |
| GCA_002807975.1 | S003202 | 2014 | Bangladesh | 4039674 | 86 |
| GCA_002807965.1 | 330073_B | 2013 | Bangladesh | 4045868 | 81 |
| GCA_002808065.1 | 330440_C1 | 2013 | Bangladesh | 4043032 | 78 |
| GCA_002808105.1 | 330081 | 2014 | Bangladesh | 4044553 | 81 |
| GCA_002808125.1 | S023202 | 2014 | Bangladesh | 4042056 | 79 |
| GCA_002808145.1 | 22087102_C2 | 2013 | Bangladesh | 4042349 | 79 |
| GCA_002808155.1 | S002604 | 2014 | Bangladesh | 4045052 | 78 |
| GCA_002808165.1 | 22043202_C1 | 2013 | Bangladesh | 4044047 | 79 |
| GCA_002808205.1 | S003008 | 2014 | Bangladesh | 4044346 | 82 |
| GCA_002808225.1 | 330898_C2 | 2013 | Bangladesh | 4041730 | 81 |
| GCA_002808275.1 | 22044108_C3 | 2013 | Bangladesh | 4042366 | 76 |
| GCA_002808325.1 | S040602_C1 | 2013 | Bangladesh | 4043126 | 75 |
| GCA_002808345.1 | 22087500_C9 | 2013 | Bangladesh | 4040182 | 77 |
| GCA_002808355.1 | S042100 | 2013 | Bangladesh | 4042390 | 84 |
| GCA_002808465.1 | 22043200_C1 | 2013 | Bangladesh | 4042896 | 82 |
| GCA_002843255.1 | VcN1 | 2017 | Bangladesh | 4145933 | 177 |
| GCA_900185995.1 | BC1071 | ND | ND | 4194576 | 2 |
| GCA_002749635.1 | E7946 | 1978 | Bahrain | 4063073 | 2 |
| GCA_002890525.1 | 11116 | 2006 | Sweden | 4059991 | 39 |
| GCA_002892855.1 | A1552 | 1992 | United States | 4085463 | 2 |
| GCA_002911455.1 | 5879 | 1972 | Russia | 3982623 | 114 |
| GCA_002946655.1 | HC1037 | 2014 | Haiti | 4061419 | 2 |
| GCA_002997215.1 | A1552 | 1992 | Peru | 4085460 | 2 |
| GCA_003015005.1 | 186 | 2011 | Ukraine | 4019066 | 97 |
| GCA_003013485.1 | 2044 | 1966 | Iraq | 4045779 | 124 |
| GCA_003056705.1 | 20-a_11 | 1995 | Ukraine | 4111774 | 203 |
| GCA_003056955.1 | M1425 | 2003 | Russia | 4025851 | 114 |
| GCA_003056975.1 | 2688 | 2015 | Russia | 3970206 | 123 |
| GCA_003056995.1 | M1332 | 2000 | Russia | 3975667 | 83 |
| GCA_003057055.1 | 2613 | 2015 | Russia | 3967932 | 125 |
| GCA_003057015.1 | 8 | 2014 | Russia | 3903289 | 121 |
| GCA_003057035.1 | 3178 | 2017 | Russia | 3998812 | 101 |
| GCA_003057075.1 | 2687 | 2015 | Russia | 3966695 | 125 |
| GCA_003057085.1 | 124 | 2015 | Russia | 3969210 | 120 |
| GCA_003057115.1 | 2843 | 2016 | Russia | 4001056 | 113 |
| GCA_003057775.1 | A3_296 | 2017 | Brazil | 3774522 | 88 |
| GCA_003063885.1 | Sa5Y | 2004 | United States | 4050878 | 2 |
| GCA_003097695.1 | A1552 | 1992 | United States | 4085468 | 2 |
| GCA_900324425.1 | 4295STDY6534232 | ND | ND | 4092645 | 2 |
| GCA_900324445.1 | 4295STDY6534216 | ND | ND | 4092641 | 2 |
| GCA_900324455.1 | 4295STDY6534248 | ND | ND | 4092644 | 2 |
| GCA_003130475.1 | 41D | 1998 | Russia | 4038688 | 140 |
| GCA_003130465.1 | 16241D | 1994 | Russia | 4081404 | 141 |
| GCA_003130485.1 | 169D | 1993 | Russia | 4102946 | 119 |
| GCA_003130495.1 | 1270D | 1994 | Russia | 4016316 | 133 |
| GCA_003096115.1 | Ogawa 18963 | 2007 | Russia | 3966209 | 66 |
| GCA_003096135.1 | 9507 | 1974 | Russia | 3997109 | 105 |
| GCA_002899735.1 | IDH-06787 | 2014 | India | 4404109 | 123 |
| GCA_002918335.1 | 146P | 1994 | India | 4012364 | 58 |
| GCA_002918345.1 | 146N | 1994 | India | 4014421 | 59 |
| GCA_003205555.1 | UG054 | 2015 | Uganda | 4041743 | 119 |
| GCA_003205655.1 | UG042 | 2015 | Uganda | 4044573 | 122 |
| GCA_003205635.1 | UG020 | 2016 | Uganda | 4023664 | 110 |
| GCA_003205565.1 | UG046 | 2015 | Uganda | 4042522 | 114 |
| GCA_003205685.1 | UG026 | 2014 | Uganda | 4024131 | 104 |
| GCA_003205705.1 | UG071 | 2014 | Uganda | 4034068 | 94 |
| GCA_003205675.1 | UG086 | 2015 | Uganda | 4035264 | 105 |
| GCA_003205755.1 | UG060 | 2014 | Uganda | 4036290 | 106 |
| GCA_003205735.1 | UG040 | 2015 | Uganda | 4036071 | 112 |
| GCA_003205765.1 | UG010 | 2016 | Uganda | 4007874 | 103 |
| GCA_003260135.1 | 60555434 | 2017 | Australia | 3918371 | 94 |
| GCA_003311755.1 | 2523-88 | ND | ND | 4180641 | 138 |
| GCA_003311805.1 | 2017V-1038 | 2017 | United States | 4028322 | 109 |
| GCA_003311815.1 | 2015V-1076 | 2015 | United States | 4050103 | 89 |
| GCA_003311825.1 | 2016V-1062 | 2016 | United States | 4021660 | 83 |
| GCA_003311885.1 | 2017V-1124 | 2017 | United States | 4137761 | 80 |
| GCA_003311865.1 | 2017V-1070 | 2017 | United States | 4082510 | 112 |
| GCA_003311895.1 | 2017V-1085 | 2017 | United States | 4038943 | 109 |
| GCA_003311905.1 | 07-2425 | ND | ND | 3832137 | 105 |
| GCA_003311945.1 | 2014V-1107 | 2014 | United States | 3987160 | 116 |
| GCA_003311965.1 | 2016V-1111 | 2016 | United States | 3896089 | 71 |
| GCA_003311975.1 | 2017V-1105 | 2017 | United States | 4080506 | 90 |
| GCA_003312005.1 | 2017V-1110 | 2017 | United States | 3993568 | 168 |
| GCA_003312035.1 | 2016V-1018 | 2016 | United States | 4189996 | 67 |
| GCA_003312015.1 | 2017V-1144 | 2017 | United States | 3868307 | 131 |
| GCA_003312065.1 | 2016V-1091 | 2016 | United States | 3814012 | 93 |
| GCA_003312085.1 | 2016V-1114 | 2016 | United States | 3872198 | 92 |
| GCA_003312095.1 | 2017V-1176 | 2017 | United States | 3896121 | 110 |
| GCA_003312945.1 | 09_113 | 2018 | Brazil | 4001106 | 128 |
| GCA_003591575.1 | FORC_073 | 2017 | South Korea | 4026094 | 2 |
