# Supplementary material for: VicPred: A Vibrio cholerae Genotype Prediction Tool
Source: Front Microbiol. 2021 Sep 9;12:691895. doi: 10.3389/fmicb.2021.691895 (PMC8458814; doi:10.3389/fmicb.2021.691895)
Supplement: Supplementary file 7 [file Table_2.docx]

**Supplementary Table 2. Representative gene list of CTX prophage.**

| **Gene** | **Biotype** | **Type** | **Reference (PMID)** |
| --- | --- | --- | --- |
| *ctxB* | Classical | *ctxB1* | 19116078 |
|  | El Tor | *ctxB2* | 25913612 |
|  | El Tor | *ctxB3* | 25913612 |
|  | El Tor | *ctxB4* | 25913612 |
|  | El Tor | *ctxB5* | 25913612 |
|  | El Tor | *ctxB6* | 25913612 |
|  | El Tor | *ctxB7* | 24722374 |
|  | El Tor | *ctxB8* | 25913612 |
|  | El Tor | *ctxB9* | 25913612 |
|  | El Tor | *ctxB10* | 25913612 |
|  | El Tor | *ctxB11* | 25913612 |
|  | El Tor | *ctxB12* | 25913612 |
| *ctxA* | El Tor | *ctxA* | 25913612 |
| *zot/ace/orfU/cep* | Classical | CTX-cla | 19116078 |
|  | El Tor | CTX-1 | 25913612 |
|  | El Tor | CTX-USGulf | 25913612 |
| *rstB* | Classical | CTX-cla | 25913612 |
|  | Classical | RS1-cla | 25913612 |
|  | El Tor | CTX-1 | 25913612 |
|  | El Tor | CTX-2 | 25913612 |
|  | El Tor | CTX-USGulf | 25913612 |
|  | El Tor | CTX-4 | 25913612 |
|  | El Tor | CTX-5 | 25913612 |
|  | El Tor | CTX-6 | 25913612 |
|  | El Tor | RS1-ET | 25913612 |
| *rstA* | Classical | CTX-cla | 25913612 |
|  | El Tor | CTX-1 | 25913612 |
|  | El Tor | CTX-2 | 25913612 |
|  | El Tor | CTX-USGulf | 25913612 |
|  | El Tor | RS1-ET | 2007761 |
|  | El Tor | RS1-env | 2007761 |
|  | El Tor | RS1-cla | 2007761 |
| *rstR* | Classical | CTX-cla | 25913612 |
|  | El Tor | CTX-1 | 25913612 |
|  | El Tor | US-Gulf | 24722374 |
|  | El Tor | CTX-O139 | 10542181 |
