# Supplementary material for: VicPred: A Vibrio cholerae Genotype Prediction Tool
Source: Front Microbiol. 2021 Sep 9;12:691895. doi: 10.3389/fmicb.2021.691895 (PMC8458814; doi:10.3389/fmicb.2021.691895)
Supplement: Supplementary file 8 [file Table_3.docx]

**Supplementary Table 3. ORFs used for finding GIs.** The genes in the four known pathogenicity islands (VPI-1, VPI-2, VSP-1, and VSP-2) of *V. cholerae* N16961 (NC_002505.1) were used for GI prediction using USERACH tool.

| GI | ORF | Description | Length(bp) |
| --- | --- | --- | --- |
| VPI-1 | VC0817 | Putative transposase | 978 |
|  | VC0818 | Uncharacterized protein | 681 |
|  | VC0819 | Aldehyde dehydrogenase | 1521 |
|  | VC0820 | ToxR-activated gene A lipoprotein | 3042 |
|  | VC0821 | Uncharacterized protein | 1697 |
|  | VC0823 | Uncharacterized protein | 939 |
|  | VC0824 | Thiol peroxidase | 495 |
|  | VC0825 | Toxin coregulated pilus biosynthesis protein I | 1863 |
|  | VC0826 | Toxin coregulated pilus biosynthesis protein P | 666 |
|  | VC0827 | Toxin coregulated pilus biosynthesis protein H | 411 |
|  | VC0828 | Toxin coregulated pilin | 675 |
|  | VC0829 | Toxin coregulated pilus biosynthesis protein B | 1293 |
|  | VC0830 | Toxin coregulated pilus biosynthesis protein Q | 453 |
|  | VC0831 | Toxin coregulated pilus biosynthesis outer membrane protein C | 1470 |
|  | VC0832 | Toxin coregulated pilus biosynthesis protein R | 456 |
|  | VC0833 | Toxin coregulated pilus biosynthesis protein D | 837 |
|  | VC0834 | Toxin coregulated pilus biosynthesis protein S | 459 |
|  | VC0835 | Toxin coregulated pilus biosynthesis protein T | 1512 |
|  | VC0836 | Toxin coregulated pilus biosynthesis protein E | 1023 |
|  | VC0837 | Toxin coregulated pilus biosynthesis protein F | 1017 |
|  | VC0838 | TCP pilus virulence regulatory protein ToxT | 831 |
|  | VC0839 | Prepilin leader peptidase/N-methyltransferase | 762 |
|  | VC0840 | Accessory colonization factor AcfB | 1881 |
|  | VC0841 | Accessory colonization factor AcfC | 771 |
|  | VC0842 | RDD domain-containing | 474 |
|  | VC0843 | TagE protein | 909 |
|  | VC0844 | Accessory colonization factor AcfA | 648 |
|  | VC0845 | Accessory colonization factor AcfD | 4563 |
|  | VC0847 | Integrase | 1269 |
| VPI-2 | VC1758 | Prophage integrase IntA | 1236 |
|  | VC1759 | Uncharacterized protein | 456 |
|  | VC1760 | Putative helicase | 2799 |
|  | VC1761 | Uncharacterized protein | 609 |
|  | VC1762 | EH Signature domain-containing protein | 1452 |
|  | VC1763 | Chemotaxis protein MotB-related protein | 735 |
|  | VC1764 | Uncharacterized protein | 2121 |
|  | VC1765 | Type I site-specific deoxyribonuclease | 3060 |
|  | VC1766 | Uncharacterized protein | 1992 |
|  | VC1767 | DUF262 domain-containing protein | 1413 |
|  | VC1768 | Methylase S domain-containing protein | 1329 |
|  | VC1769 | Site-specific DNA-methyltransferase | 2382 |
|  | VC1770 | Uncharacterized protein | 2064 |
|  | VC1771 | Uncharacterized protein | 3618 |
|  | VC1772 | WYL domain-containing protein | 861 |
|  | VC1773 | Kelch domain-containing protein | 1071 |
|  | VC1774 | N-acetylneuraminate epimerase NanM | 1155 |
|  | VC1775 | Uncharacterized protein | 837 |
|  | VC1776 | Putative N-acetylneuraminate lyase | 897 |
|  | VC1777 | Sialic acid TRAP transporter large permease protein SiaM | 1284 |
|  | VC1778 | Sialic acid TRAP transporter small permease protein SiaQ | 510 |
|  | VC1779 | Sialic acid-binding periplasmic protein SiaP | 966 |
|  | VC1781 | Putative N-acetylmannosamine-6-phosphate 2-epimerase NanE | 711 |
|  | VC1782 | N-acetylmannosamine kinase NanK | 864 |
|  | VC1783 | N-acetylglucosamine-6-phosphate deacetylase | 1137 |
|  | VC1784 | Sialidase NanH | 2346 |
|  | VC1785 | Transcriptional regulator | 177 |
|  | VC1786 | UPF0758 protein | 477 |
|  | VC1787 | Uncharacterized protein | 138 |
|  | VC1788 | Uncharacterized protein | 696 |
|  | VC1789 | Transposase OrfAB, subunit B | 1179 |
|  | VC1791 | Uncharacterized protein | 960 |
|  | VC1792 | Uncharacterized protein | 360 |
|  | VC1793 | Uncharacterized protein | 372 |
|  | VC1794 | Uncharacterized protein | 579 |
|  | VC1795 | putative transcriptional regulator | 321 |
|  | VC1796 | Middle operon regulator-related protein | 375 |
|  | VC1797 | Uncharacterized protein | 462 |
|  | VC1798 | Eha protein | 1110 |
|  | VC1799 | Integrase catalytic domain-containing protein | 1758 |
|  | VC1800 | Uncharacterized protein | 972 |
|  | VC1801 | Uncharacterized protein | 336 |
|  | VC1802 | Uncharacterized protein | 237 |
|  | VC1804 | Uncharacterized protein | 264 |
|  | VC1805 | Uncharacterized protein | 447 |
|  | VC1806 | G domain-containing protein | 987 |
|  | VC1807 | Uncharacterized protein | 641 |
|  | VC1808 | Uncharacterized protein | 846 |
|  | VC1809 | Putative transcriptional regulator | 201 |
| VSP-1 | VC0174 | SPOR domain-containing protein | 948 |
|  | VC0175 | Deoxycytidylate deaminase-related protein | 1590 |
|  | VC_RS00860 | Uncharacterized protein | 234 |
|  | VC0176 | Putative transcriptional regulator VspR | 309 |
|  | VC0177 | Transcriptional regulator VspR | 564 |
|  | VC_RS00875 | Uncharacterized protein | 249 |
|  | VC0178 | Patatin-related protein CapV | 1068 |
|  | VC0179 | Cyclic GMP-AMP synthase DncV | 1305 |
|  | VC0180 | ThiF domain-containing protein | 1755 |
|  | VC0181 | Prok-JAB domain-containing protein | 468 |
|  | VC0182 | Uncharacterized protein | 432 |
|  | VC0183 | Phage integrase Int | 2112 |
|  | VC0184 | Uncharacterized protein | 1689 |
|  | VC0185 | Putative transposase | 1203 |
|  | VC0186 | Glutathione reductase GorA | 1353 |
| VSP-2 | VC0489 | Putative hemolysin | 1761 |
|  | VC0490 | Uncharacterized protein | 1962 |
|  | VC0491 | Uncharacterized protein | 537 |
|  | VC0492 | Uncharacterized protein | 1167 |
|  | VC0493 | Uncharacterized protein | 876 |
|  | VC0494 | Uncharacterized protein | 645 |
|  | VC0495 | Uncharacterized protein | 675 |
|  | VC0496 | Uncharacterized protein | 600 |
|  | VC0497 | Transcriptional regulator | 201 |
|  | VC0498 | Putative ribonuclease HI | 441 |
|  | VC0499a | Transposase OrfAB, subunit B | 207 |
|  | VC0499b | Transposase OrfAB, subunit B | 600 |
|  | VC0500a | Transposase OrfAB, subunit a | 147 |
|  | VC0500b | Transposase | 153 |
|  | VC0501a | Transposase | 489 |
|  | VC0501b | Transposase | 450 |
|  | VC0502 | Type IV pilin, putative | 525 |
|  | VC0503 | Cell wall endopeptidase | 1281 |
|  | VC0504 | Uncharacterized protein | 228 |
|  | VC0505 | Uncharacterized protein | 369 |
|  | VC0506 | WYL domain-containing protein | 735 |
|  | VC0507 | Uncharacterized protein | 177 |
|  | VC0508 | Uncharacterized protein | 444 |
|  | VC0509 | Uncharacterized protein | 444 |
|  | VC0510 | UPF0758 protein | 474 |
|  | VC0511 | Uncharacterized protein | 120 |
|  | VC0512 | Methyl-accepting chemotaxis protein | 1590 |
|  | VC0513 | AraC family transcriptional regulator | 816 |
|  | VC0514 | Methyl-accepting chemotaxis protein | 1881 |
|  | VC0515 | Putative signal transduction protein | 1233 |
|  | VC0516 | Phage integrase | 1242 |
|  | VC0517 | RNA polymerase sigma factor RpoD | 1878 |
