# Supplementary material for: VicPred: A Vibrio cholerae Genotype Prediction Tool
Source: Front Microbiol. 2021 Sep 9;12:691895. doi: 10.3389/fmicb.2021.691895 (PMC8458814; doi:10.3389/fmicb.2021.691895)
Supplement: Supplementary file 9 [file Table_4.docx]

**Supplementary Table 4. Predicted results of O antigen serogrouping and phage (TLC, CTX prophage, and RS1) typing.** A total of 693 genomes were predicted. A total 602 genomes were having OAGC and 593 genomes were predicted to having at least one of enterotoxin related phage elements (TLC, CTX prophage, and RS1). The arrays of genes were discerned with colon (:) ; :TLC: (array of TLC1-TLC2-TLC3-TLC4-TLC5), :RS1: (*rstR*-*rstA*-*rstB*-*rstC*), :CTX: (*rstR-rstA-rstB-cep-orfU-ace-zot-ctxA-ctxB*), and :ctx: (*cep-orfU-ace-zot-ctxA-ctxB*). Because of the low quality of several genome data, the precise location of CTX prophage elements could not be determined, so we describe :CTX: and :ctx: separately. Under-bar (_) means linkage between the elements on the same contig.

| **Year** | **Strain** | **Country** | **Serogroup** | **TCP** | **Arrays of CTX and related elements (TLC, CTX prophage, and RS1)** | | **Type Genes of CTX prophage** | | | | |
| --- | --- | --- | --- | --- | --- | --- | --- | --- | --- | --- | --- |
|  |  |  |  |  | **Chromosome I** | **Chromosome II** | ***ctxB*** | ***cep*** | ***rstB*** | ***rstA*** | ***rstR*** |
| 1587 | 1587 | Peru | O12 | - | - | - | *-* | - | - | - | - |
| 1854 | ATCC 14035 | United Kingdom | O1 Ogawa | + | :TLC: :CTX: | ctxA_ctxB | *ctxB1* | CTX-cla | CTX-cla | CTX-cla | CTX-cla |
| 1937 | M66-2 | Indonesia | O1 Ogawa | + | :TLC:_:TLC: | - | *-* | - | - | - | - |
| 1937 | MAK 676 | Indonesia | O1 Ogawa | + | :TLC: :CTX: | - | *ctxB1* | CTX-1 | CTX-cla | CTX-cla | CTX-cla |
| 1937 | MAK 97 | Indonesia | O1 Inaba | + | - | - | *-* | - | - | - | - |
| 1937 | MAK 757 | Indonesia | O1 Ogawa | + | :TLC:_:TLC:_:CTX: | - | *ctxB1* | CTX-1 | CTX-cla | CTX-cla | CTX-cla |
| 1938 | NCTC 5395 | Iraq | O1 Ogawa | + | :TLC:_:TLC: | - | *-* | - | - | - | - |
| 1941 | NIH41 | India | NA | + | :TLC: :ctx: rstC rstA_rstR | - | *ctxB3* | CTX-1 | - | RS1-ET | CTX-1 |
| 1942 | M29 | Russia | O1 Ogawa | + | :TLC: :ctx: rstB_rstA_rstR | - | *ctxB1* | CTX-cla | CTX-cla | CTX-cla | CTX-cla |
| 1949 | A68 | Egypt | O1 Inaba | + | :TLC: cep_orfU_ace_zot ctxB_ctxA rstB_rstA_rstR | - | *ctxB1* | CTX-cla | CTX-cla | CTX-cla | CTX-cla |
| 1954 | NCTC 9420 | Egypt | O1 Ogawa | + | :TLC: | - | *-* | - | - | - | - |
| 1957 | A6 | Indonesia | O1 Ogawa | + | :TLC: :CTX: | - | *ctxB3* | CTX-1 | CTX-1 | CTX-1 | CTX-1 |
| 1957 | C5 | Indonesia | O1 Ogawa | + | :TLC:_:TLC:_:CTX: | - | *ctxB3* | CTX-1 | CTX-1 | CTX-1 | CTX-1 |
| 1958 | D-35 | Bangladesh | O1 Ogawa | + | :TLC: :ctx: rstR_rstA_rstB | - | *ctxB1* | CTX-cla | CTX-cla | CTX-cla | CTX-cla |
| 1958 | A60 | Thailand | O1 Inaba | + | :TLC: :CTX: | - | *ctxB1* | CTX-cla | CTX-cla | CTX-cla | CTX-cla |
| 1961 | E9120 | Indonesia | O1 Ogawa | - | :TLC:_:TLC:_:CTX: | - | *ctxB3* | CTX-1 | CTX-1 | CTX-1 | CTX-1 |
| 1962 | 10432-62 | Philippines | O27 | - | - | - | *-* | - | - | - | - |
| 1962 | A66 | Bangladesh | O1 Inaba | + | :TLC: :CTX: | TLC1 | *ctxB1* | CTX-cla | CTX-cla | CTX-cla | CTX-cla |
| 1962 | A49 | ND | NA | + | :TLC: :CTX: | - | *ctxB1* | CTX-cla | CTX-cla | RS1-cla\|CTX-cla | CTX-cla |
| 1962 | CRC1106 | India | O1 Ogawa | + | :TLC:_:TLC:_:RS1:_:CTX: | - | *ctxB3* | CTX-1 | CTX-1\|RS1-ET | CTX-1\|RS1-ET | CTX-1 |
| 1962 | E1162 | China | O1 Ogawa | + | :TLC:_:TLC:_:CTX:_:RS1:_:CTX: | - | *ctxB3* | CTX-1 | CTX-1\|RS1-ET\|CTX-1 | CTX-1\|RS1-ET\|CTX-1 | CTX-1 |
| 1963 | FDAARGOS_102 | India | O37 | + | :TLC:_:CTX:_:TLC:_:CTX:_:TLC:_:CTX: | rstC | *ctxB9* | CTX-USGulf | CTX-1 | CTX-cla | CTX-1 |
| 1964 | A46 | ND | O1 Ogawa | + | :TLC: rstR_rstA_rstB | :ctx: | *ctxB1* | CTX-cla | CTX-cla | CTX-cla | CTX-cla |
| 1964 | CRC711 | India | O1 Ogawa | + | :TLC:_:TLC:_:RS1:_:CTX: | - | *ctxB3* | CTX-1 | CTX-1\|RS1-ET | CTX-1\|RS1-ET | CTX-1 |
| 1965 | O395 | India | O1 Ogawa | + | :TLC:_:TLC:_:TLC:_rstR_rstA_rstB_:CTX: | :CTX: | *ctxB1* | CTX-cla | CTX-cla | CTX-cla | CTX-cla |
| 1965 | M299 | Turkmenistan | O1 Inaba | + | :TLC: | - | *-* | - | - | - | - |
| 1966 | 5/66 | Pakistan | O1 Inaba | + | :TLC: :ctx: rstB_rstA_rstR | - | *ctxB1* | CTX-cla | CTX-cla | CTX-cla | CTX-cla |
| 1966 | CW-6 | India | O1 Ogawa | + | :TLC: zot_ace_orfU_cep_rstB_rstA_rstR | - | *-* | CTX-1 | CTX-2 | CTX-1 | CTX-cla |
| 1966 | 2044 | Iraq | O1 Ogawa | + | :TLC: :ctx: rstC rstB_rstA_rstR | - | *ctxB3* | CTX-1 | RS1-ET | RS1-ET | CTX-1 |
| 1967 | 6/67 | India | O1 ND | + | :TLC: :ctx: rstR_rstA rstC | - | *ctxB4* | CTX-1 | - | RS1-ET | CTX-1 |
| 1969 | A70 | Bangladesh | O1 Inaba | + | :TLC: :CTX: | - | *ctxB1* | CTX-cla | CTX-cla | CTX-cla | CTX-cla |
| 1970 | M818 | Russia | O1 Inaba | + | :TLC: :ctx:_rstB rstR rstC_rstB | - | *ctxB3* | CTX-1 | CTX-1\|RS1-ET | CTX-2\|RS1-ET\|RS1-env | CTX-1 |
| 1970 | A59 | India | O1 Inaba | + | :TLC: rstA_rstB orfU_ace_zot_ctxA_ctxB rstR | - | *ctxB1* | - | CTX-cla | CTX-cla | CTX-cla |
| 1970 | A61 | India | O1 Inaba | + | :TLC: rstR_rstA_rstB ctxB_ctxA zot_ace_orfU_cep | - | *ctxB1* | CTX-cla | CTX-cla | CTX-cla | CTX-cla |
| 1970 | GP8 | India | O1 Inaba | + | :TLC: :CTX: | - | *ctxB1* | CTX-cla | CTX-cla | CTX-cla | CTX-cla |
| 1970 | M888D | Russia | NA | + | :TLC: :RS1: | - | *-* | - | RS1-ET | RS1-ET | CTX-1 |
| 1970 | M888 | Russia | O1 Inaba | + | :TLC: rstA_rstB_:ctx: rstA_rstR rstA_rstB_rstC | - | *ctxB3* | CTX-1 | CTX-1\|RS1-ET | CTX-2\|RS1-ET\|RS1-env | CTX-1 |
| 1971 | GP16 | India | O1 Inaba | + | :TLC: :CTX: | - | *ctxB1* | CTX-cla | CTX-cla | CTX-cla | CTX-cla |
| 1971 | A19 | ND | O1 var Inaba | + | :TLC:_:TLC:_:CTX:_:RS1: | - | *ctxB3* | CTX-1 | RS1-ET\|CTX-1 | RS1-ET\|CTX-1 | CTX-1 |
| 1972 | M988 | Turkmenistan | O1 Inaba | - | - | - | *-* | - | - | - | - |
| 1972 | 5879 | Russia | O1 Inaba | + | :TLC:_rstR_rstA_rstB rstR_rstB_:CTX: rstA_rstB_rstC | - | *ctxB3* | CTX-1 | RS1-ET\|RS1-ET\|CTX-6\|RS1-ET | RS1-ET | CTX-1 |
| 1973 | A4 | ND | O1 Ogawa | + | :TLC: :RS1: :ctx: | - | *ctxB3* | CTX-1 | RS1-ET | RS1-ET | CTX-1 |
| 1974 | 1154-74 | India | O49 | - | :TLC: | - | *-* | - | - | - | - |
| 1974 | E1320 | China | O1 Ogawa | + | :TLC:_:TLC: | :CTX:_:CTX: | *ctxB12* | CTX-USGulf | CTX-cla | CTX-cla | CTX-cla |
| 1974 | E506 | United States | O1 Inaba | + | :TLC:_:TLC:_:CTX:_:CTX: | rstA_rstB_rstC_rstA_rstB_rstC | *ctxB1\|ctxB5* | CTX-USGulf | CTX-USGulf | CTX-USGulf | CTX-USGulf |
| 1974 | 3225-74 | Guam | NA | - | - | - | *-* | - | - | - | - |
| 1975 | N16961 | Bangladesh | O1 var Inaba | + | :TLC:_:TLC:_:CTX:_:RS1: | - | *ctxB3* | CTX-1 | RS1-ET\|CTX-1 | RS1-ET\|CTX-1 | CTX-1 |
| 1975 | 981-75 | India | O65 | + | rstR | rstB_cep_orfU_ace_zot | *-* | CTX-1 | CTX-2 | - | CTX-cla |
| 1976 | 8-76 | India | O77 | - | - | - | *-* | - | - | - | - |
| 1977 | 1421-77 | India | O80 | + | rstC | - | *-* | - | - | - | - |
| 1977 | A18 | India | O1 Inaba | + | :TLC: :CTX: rstC | - | *ctxB3* | CTX-1 | CTX-1 | CTX-1 | CTX-1 |
| 1977 | M2140 | Australia | O1 var | + | :TLC:_:TLC:_:CTX:_:CTX: | - | *ctxB2* | CTX-1 | CTX-1 | CTX-2 | CTX-cla |
| 1978 | GP143 | Bahrain | O1 var | + | :TLC: rstC rstR_rstA_rstB ace_orfU | - | *-* | - | RS1-ET | RS1-ET | CTX-1 |
| 1978 | GP140 | Malaysia | NA | + | :TLC: :CTX: rstB_rstA_rstR | - | *ctxB9* | CTX-1 | RS1-ET\|CTX-1 | RS1-ET | CTX-1 |
| 1978 | 116063 | Brazil | O1 Ogawa | - | - | - | *-* | - | - | - | - |
| 1978 | 2559-78 | United States | O1 Inaba | + | :TLC: :ctx:_rstB rstR | - | *ctxB1* | CTX-USGulf | CTX-USGulf | - | CTX-USGulf |
| 1978 | 2631-78 | United States | O1 Ogawa | - | - | - | *-* | - | - | - | - |
| 1978 | 2633-78 | Brazil | O1 Ogawa | - | - | - | *-* | - | - | - | - |
| 1978 | 1074-78 | Brazil | O1 Ogawa | - | - | - | *-* | - | - | - | - |
| 1978 | E7946 | Bahrain | O1 Ogawa | + | :TLC:_:TLC:_:CTX:_:RS1: | - | *ctxB3* | CTX-1 | RS1-ET\|CTX-1 | RS1-ET\|CTX-1 | CTX-1 |
| 1979 | GP145 | India | O1 Ogawa | + | :TLC: :RS1: :CTX: | - | *ctxB3* | CTX-1 | CTX-1\|RS1-ET | RS1-ET | CTX-1 |
| 1979 | GP152 | India | O1 Inaba | + | :TLC: rstB_rstC_rstR_rstA cep_orfU_ace ctxB_ctxA | - | *ctxB3* | CTX-1 | RS1-ET | RS1-ET | CTX-1 |
| 1979 | A10 | Bangladesh | O1 Ogawa | + | :TLC: :CTX: :RS1: | - | *ctxB3* | CTX-1 | CTX-1\|RS1-ET | RS1-ET | CTX-1 |
| 1979 | A22 | Bangladesh | O1 Inaba | + | :TLC:_:CTX:_:RS1: | - | *ctxB3* | CTX-1 | CTX-1\|CTX-6 | RS1-ET | CTX-1 |
| 1980 | TM 11079-80 | Brazil | O1 Ogawa | - | - | - | *-* | - | - | - | - |
| 1980 | PRL5 | India | O1 Ogawa | + | :TLC: :CTX:_rstC | - | *ctxB3* | CTX-1 | CTX-6 | RS1-ET | CTX-1 |
| 1980 | GP160 | India | O1 Ogawa | + | :TLC: :CTX:_:RS1: | - | *ctxB3* | CTX-1 | CTX-1\|RS1-ET | RS1-ET | CTX-1 |
| 1980 | 2740-80 | United States | O1 Inaba | + | :TLC:_:TLC: | - | *-* | - | - | - | - |
| 1981 | 984-81 | India | O89 | - | - | - | *-* | - | - | - | - |
| 1981 | VC22 | United States | O1 Ogawa | - | - | - | *-* | - | - | - | - |
| 1982 | TMA 21 | Brazil | Oa1 | - | - | - | *-* | - | - | - | - |
| 1982 | A76 | Bangladesh | O1 var Inaba | + | :TLC: rstB_rstA_rstR zot_ace_orfU_cep | ctxA_ctxB | *ctxB1* | CTX-cla | CTX-cla | CTX-cla | CTX-cla |
| 1982 | M1399 | Russia | O1 Ogawa | + | :TLC: | - | *-* | - | - | - | - |
| 1983 | 87395 | Mexico | Oa2 | - | - | - | *-* | - | - | - | - |
| 1984 | A213 | Georgia | O1 Inaba | + | :TLC: | - | *-* | - | - | - | - |
| 1985 | RC9 | Kenya | O1 Ogawa | + | :TLC: :RS1:_:ctx:_rstB_rstA | - | *ctxB3* | CTX-1 | RS1-ET\|CTX-1 | RS1-ET\|CTX-1 | CTX-1 |
| 1985 | 12129(1) | Australia | O1 var Inaba | - | - | - | *-* | - | - | - | - |
| 1986 | BX 330286 | Australia | O1 var | + | :TLC:_:CTX: rstA_rstB_rstC | - | *ctxB2* | CTX-1 | RS1-cla\|CTX-1 | RS1-env\|CTX-2 | CTX-cla |
| 1986 | 2512-86 | United States | O1 Inaba | + | :TLC: :CTX:_:CTX: | - | *ctxB1* | CTX-USGulf | CTX-USGulf | CTX-USGulf | CTX-USGulf |
| 1986 | 1496-86 | United States | O1 Inaba | + | :TLC: | - | *-* | - | - | - | - |
| 1986 | G_33 | Guinea | O1 Ogawa | + | :TLC: :ctx: rstC rstR_rstA | - | *ctxB3* | CTX-1 | - | RS1-ET | CTX-1 |
| 1987 | V51 | United States | NA | + | :RS1: ctxA_ctxB_rstR rstB_cep_orfU_ace_zot_ctxA rstR rstC_rstC rstC | rstB_rstC | *ctxB1* | CTX-USGulf | CTX-USGulf | CTX-USGulf | CTX-USGulf |
| 1987 | A389 | Bangladesh | O1 Ogawa | + | :TLC: :CTX: | - | *ctxB1* | CTX-cla | CTX-cla | CTX-cla | CTX-cla |
| 1987 | 95412 | Mexico | O1 Inaba | + | :TLC: :ctx: rstB_rstA_rstR | - | *ctxB1* | CTX-cla | CTX-cla | CTX-cla | CTX-cla |
| 1988 | 571-88 | China | NA | + | :CTX: | - | *ctxB12* | CTX-USGulf | CTX-4 | CTX-2 | CTX-1 |
| 1988 | P13762 | Uzbekistan | O1 Ogawa | + | :TLC: rstB_rstC rstB_cep_orfU_ace rstR ctxB_ctxA rstR | - | *ctxB1* | CTX-1 | RS1-ET\|CTX-1 | - | CTX-1\|CTX-cla |
| 1989 | YN89004 | China | O1 Ogawa | + | :TLC: rstR_rstA :ctx:_rstR_rstA | - | *ctxB3* | CTX-1 | - | RS1-ET | CTX-1 |
| 1989 | V5 | India | O1 Ogawa | + | - | - | *-* | - | - | - | - |
| 1989 | A245 | Vietnam | O1 Ogawa | + | :TLC: cep_orfU_ace_zot_ctxA rstR rstC_rstB_rstA | - | *-* | CTX-1 | RS1-ET | RS1-ET | CTX-1 |
| 1989 | A241 | Vietnam | O1 var Inaba | + | :TLC: rstA rstR :ctx: rstB_rstC | - | *ctxB3* | CTX-1 | RS1-ET | RS1-ET | CTX-1 |
| 1989 | A131 | India | O1 var | + | :TLC: cep_ace_zot_ctxA_ctxB rstA_rstR rstC | - | *ctxB3* | CTX-1 | - | CTX-1 | CTX-1 |
| 1989 | A5 | Angola | O1 Ogawa | + | :TLC: :RS1:_:ctx: | ctxA_ctxB | *ctxB3* | CTX-1 | RS1-ET | RS1-ET | CTX-1 |
| 1989 | 2521-89 | United States | Oa3 | - | - | - | *-* | - | - | - | - |
| 1990 | IEC224 | Brazil | O1 var Inaba | + | :TLC:_:TLC:_:CTX:_:RS1: | - | *ctxB3* | CTX-1 | RS1-ET\|CTX-5 | CTX-1\|RS1-ET | CTX-1 |
| 1990 | V109 | India | O1 var Ogawa | - | :TLC: rstA_rstR :ctx: rstC | - | *ctxB3* | CTX-1 | - | RS1-ET | CTX-1 |
| 1990 | A103 | ND | O1 var | + | :TLC: :CTX: | - | *ctxB1* | CTX-cla | CTX-cla | CTX-cla | CTX-cla |
| 1991 | CP1032(5) | Mexico | O1 Ogawa | + | :TLC:_:RS1: | :CTX: | *ctxB1* | CTX-1 | CTX-1\|RS1-ET | RS1-cla\|RS1-ET | CTX-cla\|CTX-1 |
| 1991 | CP1044(17) | Peru | O1 Ogawa | + | :TLC: :ctx: rstB_rstA_rstR rstC | - | *ctxB3* | CTX-1 | RS1-ET | CTX-1 | CTX-1 |
| 1991 | Amazonia | Brazil | O1 Ogawa | - | - | - | *-* | - | - | - | - |
| 1991 | A32 | Peru | O1 Inaba | + | :TLC: :ctx: | :RS1: | *ctxB3* | CTX-1 | RS1-ET | RS1-ET | CTX-1 |
| 1991 | A152 | Mozambique | O1 Ogawa | + | :TLC: :RS1: rstR_rstA_rstB_cep_orfU_ace_zot_ctxB | - | *ctxB3* | CTX-1 | CTX-1\|RS1-ET | CTX-1 | CTX-1 |
| 1991 | V212-1 | India | O1 Ogawa | + | :TLC: :ctx:_rstB_rstA rstR rstR rstC | - | *ctxB1* | CTX-1 | CTX-1 | CTX-2 | CTX-1\|CTX-cla |
| 1991 | A27 | Peru | O1 Inaba | + | :TLC: :ctx: rstB_rstA_rstR rstC | - | *ctxB3* | CTX-1 | RS1-ET | CTX-1 | CTX-1 |
| 1991 | MG116025 | Bangladesh | O1 Ogawa | + | :TLC: :RS1:_:CTX: | - | *ctxB3* | CTX-1 | RS1-ET\|CTX-1 | RS1-ET | CTX-1 |
| 1991 | A154 | Mozambique | O1 Ogawa | + | :TLC: :RS1: :CTX: | - | *ctxB3* | CTX-1 | RS1-ET\|CTX-1 | CTX-1 | CTX-1 |
| 1991 | A29 | Peru | O1 Inaba | + | :TLC: :CTX: rstC | - | *ctxB3* | CTX-1 | CTX-6 | CTX-1 | CTX-1 |
| 1991 | A31 | Peru | O1 Inaba | + | :TLC: :RS1:_:ctx: | - | *ctxB3* | CTX-1 | RS1-ET | CTX-1 | CTX-1 |
| 1991 | MG116226 | Bangladesh | O1 Ogawa | + | :TLC: :CTX:_:RS1: | - | *ctxB3* | CTX-1 | CTX-6\|CTX-1 | RS1-ET | CTX-1 |
| 1991 | AG-7404 | Bangladesh | O1 Ogawa | + | :TLC: :ctx: rstR rstC rstR | - | *ctxB1* | CTX-1 | - | - | CTX-1\|CTX-cla |
| 1991 | AG-8040 | Bangladesh | NA | + | :TLC: rstC rstR | rstR :ctx: | *ctxB1* | CTX-1 | - | - | CTX-1\|CTX-cla |
| 1991 | C6706 | Peru | O1 Inaba | + | :TLC: :ctx: rstC rstB rstR | - | *ctxB3* | CTX-1 | RS1-ET | - | CTX-1 |
| 1992 | MO10 | India | O139 | + | :TLC:_:CTX: | - | *ctxB3* | CTX-1 | CTX-1 | CTX-1 | CTX-1 |
| 1992 | 63-93 (MO45) | India | O139 | + | :TLC: rstB_:ctx: rstR rstB_rstC | - | *ctxB3* | CTX-1 | CTX-1\|RS1-ET | - | CTX-1 |
| 1992 | A201 | Argentina | O1 Inaba | + | :TLC: ctxB_ctxA_ace_orfU_cep rstR_rstA_rstB rstC | - | *ctxB3* | CTX-1 | CTX-5 | RS1-ET | CTX-1 |
| 1992 | PRL64 | India | O1 Ogawa | + | :TLC: :CTX: rstC_rstR_rstA_rstB | - | *ctxB1* | CTX-1 | CTX-5\|CTX-1 | RS1-ET | CTX-1 |
| 1992 | A193 | Bolivia | NA | + | :TLC: :ctx: rstC rstR_rstA_rstB | - | *ctxB3* | CTX-1 | RS1-ET | CTX-1 | CTX-1 |
| 1992 | A177 | Colombia | O1 Inaba | + | :TLC: :CTX: rstR_rstA_rstB rstC | - | *ctxB3* | CTX-1 | CTX-5 | RS1-ET | CTX-1 |
| 1992 | A185 | Colombia | O1 var Ogawa | + | :TLC: :CTX: rstR rstC rstB | - | *ctxB3* | CTX-1 | CTX-5 | RS1-ET | CTX-1 |
| 1992 | A200 | Argentina | O1 Ogawa | + | :TLC: :ctx:_rstR rstA_rstB rstC | - | *ctxB3* | CTX-1 | RS1-ET | CTX-1 | CTX-1 |
| 1992 | A186 | Argentina | O1 Ogawa | + | :TLC: :CTX: rstC | - | *ctxB3* | CTX-1 | CTX-6 | CTX-1 | CTX-1 |
| 1992 | 116059 | Brazil | O1 Ogawa | + | :TLC: :ctx: rstB_rstA_rstR rstC | - | *ctxB3* | CTX-1 | CTX-5 | CTX-1 | CTX-1 |
| 1992 | A1552 | Peru | O1 Inaba | + | :TLC:_:TLC:_:CTX:_:RS1: | - | *ctxB3* | CTX-1 | CTX-5\|RS1-ET | CTX-1\|RS1-ET | CTX-1 |
| 1992 | A1552 | Peru | O1 Inaba | + | :TLC:_:TLC:_:CTX:_:RS1: | - | *ctxB3* | CTX-1 | CTX-5\|RS1-ET | CTX-1\|RS1-ET | CTX-1 |
| 1992 | A1552 | Peru | O1 Inaba | + | :TLC:_:TLC:_:CTX:_:RS1: | - | *ctxB3* | CTX-1 | CTX-5\|RS1-ET | CTX-1\|RS1-ET | CTX-1 |
| 1993 | 234-93 | India | NA | + | rstR rstR rstC zot_ace rstB_rstA cep ctxB_ctxA | - | *ctxB1* | CTX-USGulf | CTX-USGulf | CTX-USGulf | CTX-USGulf\|CTX-O139 |
| 1993 | 254-93 | India | O144 | - | - | - | *-* | - | - | - | - |
| 1993 | 4260B | Bangladesh | O139 | + | :TLC: :ctx: rstA_rstR | - | *ctxB3* | CTX-1 | - | CTX-1 | CTX-1 |
| 1993 | A325 | Argentina | O1 var Inaba | - | - | - | *-* | - | - | - | - |
| 1993 | A330 | India | O139 var | + | :TLC: :CTX:_rstC rstR_rstA | - | *ctxB3* | CTX-1 | CTX-1 | RS1-ET\|CTX-1 | CTX-1 |
| 1993 | M1275 | Russia | O1 Ogawa | + | :TLC: :ctx:_rstB rstR rstB_rstC rstR | - | *ctxB1* | CTX-1 | RS1-ET\|CTX-1 | - | CTX-cla\|CTX-1 |
| 1993 | 169D | Russia | O1 Ogawa | + | :TLC: :ctx: rstR rstR rstC | - | *ctxB1* | CTX-1 | - | - | CTX-cla\|CTX-1 |
| 1994 | MJ-1236 | Bangladesh | O1 Inaba | + | - | :CTX: | *ctxB1* | CTX-1 | CTX-1 | RS1-cla | CTX-cla |
| 1994 | M-1293 | Russia | O1 Ogawa | + | :TLC: :ctx: rstR rstR rstA rstC | - | *ctxB1* | CTX-1 | - | RS1-cla | CTX-1\|CTX-cla |
| 1994 | MJ1485 | Bangladesh | NA | + | - | :CTX: | *ctxB8* | CTX-1 | CTX-1 | RS1-cla | CTX-cla |
| 1994 | A346(1) | Bangladesh | O1 Ogawa | + | :TLC: :ctx:_:RS1:_rstR_ctxA_ctxB rstR | rstC | *ctxB1* | CTX-1 | CTX-1 | RS1-cla | CTX-1\|CTX-cla |
| 1994 | I-1181 | Russia | NA | + | :TLC: rstC_rstB_rstA :ctx:_rstB_rstA | - | *ctxB1* | CTX-1 | RS1-ET\|CTX-1 | RS1-ET\|RS1-cla | - |
| 1994 | 43 | Ukraine | NA | + | :TLC: :ctx: rstC rstR rstA_rstB rstR | - | *ctxB1* | CTX-1 | RS1-ET | CTX-2 | CTX-cla\|CTX-1 |
| 1994 | 56 | Ukraine | O1 Ogawa | + | :CTX: | - | *ctxB1* | CTX-1 | CTX-1 | RS1-cla | CTX-cla |
| 1994 | I-1187 | Russia | NA | + | TLC1 TLC1 rstC_rstB :ctx:_rstB | - | *ctxB1* | CTX-1 | RS1-ET\|CTX-1 | - | - |
| 1994 | FC1817 | India | O139 | + | :TLC: :CTX:_:RS1: | - | *ctxB3* | CTX-1 | CTX-1 | CTX-1 | CTX-1 |
| 1994 | 56 | Ukraine | O1 Ogawa | + | :TLC: | - | *-* | - | - | - | - |
| 1994 | 16241D | Russia | NA | + | :TLC: :ctx: rstC rstB_rstA | - | *ctxB1* | CTX-1 | CTX-1 | RS1-cla | - |
| 1994 | 1270D | Russia | NA | + | :CTX: | - | *ctxB1* | CTX-1 | CTX-1 | RS1-cla | CTX-cla |
| 1994 | 146P | India | O139 | + | :TLC: cep TLC1 zot_ctxA_ctxB_rstR zot rstR rstC_rstB rstR | - | *ctxB3* | CTX-1 | CTX-1 | - | CTX-O139\|CTX-cla\|CTX-1 |
| 1994 | 146N | India | O139 | + | :TLC: | - | *-* | - | - | - | - |
| 1995 | CP1046(19) | Peru | O1 Ogawa | + | - | :TLC:_:RS1: | *-* | - | RS1-ET | CTX-1 | CTX-1 |
| 1995 | CP1047(20) | Peru | O1 Ogawa | + | :TLC: :CTX: rstC | - | *ctxB3* | CTX-1 | RS1-ET | CTX-1 | CTX-1 |
| 1995 | 4110 | Vietnam | NA | + | :CTX:_rstR_rstA_rstB_cep_orfU_ace_zot | - | *ctxB1* | CTX-1 | CTX-1 | RS1-cla | CTX-cla |
| 1995 | FC1877 | India | O139 | + | :TLC: rstC :ctx:_rstB rstR | - | *ctxB3* | CTX-1 | CTX-1 | - | CTX-1 |
| 1995 | 20-a_11 | Ukraine | NA | + | :TLC: :ctx: rstR rstR rstC | - | *ctxB1* | CTX-1 | - | - | CTX-1\|CTX-cla |
| 1997 | R17644 | Russia | O1 Ogawa | + | :TLC: rstB_rstA_rstR rstB_:ctx: ctxB rstB_rstC | - | *ctxB2\|ctxB1* | CTX-1 | CTX-USGulf\|CTX-4\|RS1-ET | RS1-ET | CTX-1 |
| 1997 | YN97083 | China | O1 Inaba | + | :CTX: | - | *ctxB1* | CTX-1 | CTX-1 | RS1-cla | CTX-cla |
| 1997 | I-1263 | Russia | NA | + | TLC1 rstC_rstB TLC1 :ctx:_rstB | - | *ctxB11* | CTX-1 | RS1-ET\|CTX-1 | - | - |
| 1997 | FC3611b | India | NA | + | :TLC: rstC rstA_rstB rstR rstR ctxB_ctxA | - | *ctxB5* | - | CTX-2 | CTX-2 | CTX-O139\|CTX-1 |
| 1997 | FC2271 | India | NA | + | :TLC: :ctx: rstB_rstA_rstR rstC | - | *ctxB5* | CTX-1 | CTX-1 | CTX-1 | CTX-1 |
| 1998 | YN98296 | China | O139 | + | :TLC: :ctx:_rstB rstC_rstB rstR_rstA | - | *ctxB3* | CTX-1 | CTX-1\|RS1-ET | CTX-1 | CTX-1 |
| 1998 | M1327 | Russia | O1 Ogawa | + | :TLC: rstR rstR :ctx: rstC | - | *ctxB1* | CTX-1 | - | - | CTX-cla\|CTX-1 |
| 1998 | FC2273 | India | O139 | + | :TLC: :ctx: rstB_rstA_rstR rstC | - | *ctxB5* | CTX-1 | CTX-1 | CTX-1 | CTX-1 |
| 1998 | 41D | Russia | O1 Inaba | + | :TLC: :ctx: rstR | - | *ctxB1* | CTX-1 | - | - | CTX-cla |
| 1999 | I-1300 | Russia | O1 var Inaba | + | rstC_rstB_:ctx:_rstB | - | *ctxB1* | CTX-1 | RS1-ET\|CTX-1 | - | - |
| 1999 | 4679 | Bangladesh | O1 var Inaba | + | :TLC:_rstR_rstA_rstB_cep_rstB_rstC_rstR_rstA orfU_ace_zot_ctxB | - | *ctxB1* | CTX-1 | RS1-ET\|CTX-1 | RS1-ET | CTX-1 |
| 1999 | FC3611a | India | O139 | + | :TLC: ctxB_ctxA rstA_rstB rstC rstR rstR | - | *ctxB5* | - | CTX-2 | CTX-1 | CTX-O139\|CTX-1 |
| 2000 | CP1033(6) | Mexico | O1 Ogawa | + | :TLC:_rstR_rstA | rstC_rstB ctxB_ctxA_zot_ace_orfU | *ctxB1* | - | RS1-ET | RS1-ET | CTX-1 |
| 2000 | 4672 | Bangladesh | NA | + | rstC_ctxA_zot_ace_orfU_cep_rstB_rstA rstR rstR | - | *-* | CTX-1 | CTX-4 | RS1-cla | CTX-cla\|CTX-1 |
| 2000 | TP | United States | Oa4 | - | rstB_rstA | - | *-* | - | CTX-USGulf | CTX-USGulf | - |
| 2000 | FC1384 | India | O139 | + | :TLC: ace_orfU_cep rstR rstR ctxB_ctxA rstB_rstA_rstR rstC | - | *ctxB5* | CTX-1 | CTX-2 | CTX-1 | CTX-1\|CTX-O139\|CTX-cla |
| 2001 | G4222 | South Africa | O1 Ogawa | + | - | :CTX: | *ctxB1* | CTX-1 | CTX-1 | RS1-cla | CTX-cla |
| 2001 | 4661 | Bangladesh | NA | + | :TLC: :TLC: ace_orfU_cep_rstB_rstA_rstR rstC | - | *-* | CTX-1 | CTX-1 | RS1-ET | CTX-1 |
| 2001 | 4663 | Bangladesh | O1 Inaba | + | - | :TLC:_:RS1:_:CTX: | *ctxB1* | CTX-1 | RS1-ET\|CTX-USGulf\|CTX-4 | RS1-ET | CTX-1 |
| 2001 | 4662 | Bangladesh | O1 Inaba | + | :TLC: :RS1: :ctx:_rstB | - | *ctxB1* | CTX-1 | RS1-ET\|CTX-1 | RS1-ET | CTX-1 |
| 2001 | 4675 | Bangladesh | NA | + | :TLC:_rstC_rstR_rstA_rstB_cep_orfU_ace | ctxB | *ctxB5* | CTX-1 | RS1-ET\|CTX-1 | RS1-ET | CTX-1 |
| 2001 | FC1225 | India | O139 | + | :TLC: ace rstC cep rstA_rstR_ctxB_ctxA rstR | - | *ctxB5* | CTX-1 | - | CTX-1 | CTX-O139\|CTX-1 |
| 2001 | VC0101557 | South Korea | O1 Inaba | + | rstC :CTX: | - | *ctxB1* | CTX-1 | CTX-1 | RS1-cla | CTX-cla |
| 2002 | CIRS 101 | Bangladesh | O1 Inaba | + | :TLC: :CTX:_rstC_rstB | - | *ctxB1* | CTX-1 | CTX-1\|RS1-ET | RS1-ET | CTX-1 |
| 2002 | A383 | Bangladesh | O139 | + | :TLC: rstC ctxB_ctxA rstR ace_cep_rstB_rstA_rstR | - | *ctxB5* | CTX-1 | CTX-2 | CTX-1 | CTX-1\|CTX-O139 |
| 2002 | 4111 | Vietnam | NA | + | - | :CTX: | *ctxB1* | CTX-1 | CTX-1 | RS1-cla | CTX-cla |
| 2002 | 877-163 | Bangladesh | O16 | - | - | - | *-* | - | - | - | - |
| 2002 | CISM_0035 | Mozambique | O1 Ogawa | + | :TLC: :RS1:_:CTX: | - | *ctxB1* | CTX-1 | CTX-4\|RS1-ET | RS1-ET | CTX-1 |
| 2002 | CISM_0034 | Mozambique | O1 Ogawa | + | :TLC: :RS1:_:CTX: | - | *ctxB1* | CTX-1 | CTX-1 | RS1-ET | CTX-1 |
| 2002 | CISM_0019 | Mozambique | O1 Ogawa | + | :TLC: :RS1:_:CTX: | - | *ctxB1* | CTX-1 | CTX-4\|RS1-ET | RS1-ET | CTX-1 |
| 2002 | CISM_0018 | Mozambique | O1 Ogawa | + | :TLC: :RS1:_:CTX: | - | *ctxB1* | CTX-1 | RS1-ET\|CTX-4 | RS1-ET | CTX-1 |
| 2002 | CISM_0017 | Mozambique | O1 Ogawa | + | :TLC: :RS1:_:CTX: | - | *ctxB1* | CTX-1 | CTX-4\|RS1-ET | RS1-ET | CTX-1 |
| 2002 | CISM_0016 | Mozambique | O1 Ogawa | + | :TLC: :RS1:_:CTX: | - | *ctxB1* | CTX-1 | CTX-1 | RS1-ET | CTX-1 |
| 2002 | CISM_0015 | Mozambique | O1 Ogawa | + | :TLC: :RS1:_:CTX: | - | *ctxB1* | CTX-1 | CTX-1 | RS1-ET | CTX-1 |
| 2002 | CISM_0014 | Mozambique | O1 Ogawa | + | :TLC: :RS1:_:CTX: | - | *ctxB1* | CTX-1 | CTX-4\|RS1-ET | RS1-ET | CTX-1 |
| 2002 | CISM_0010 | Mozambique | O1 Ogawa | + | :TLC: :RS1:_:CTX: | - | *ctxB1* | CTX-1 | CTX-4\|RS1-ET | RS1-ET | CTX-1 |
| 2002 | CISM_0005 | Mozambique | O1 Ogawa | + | :TLC: :RS1:_:CTX: | - | *ctxB1* | CTX-1 | CTX-1 | RS1-ET | CTX-1 |
| 2002 | FC1341 | India | O139 | + | :TLC: rstC_rstB rstA_rstR rstB_:ctx: | - | *ctxB4* | CTX-1 | CTX-2\|CTX-1 | CTX-1 | CTX-1 |
| 2003 | CP1038(11) | Zimbabwe | O1 Ogawa | + | :TLC: :ctx:_rstB rstC_rstB | - | *ctxB1* | CTX-1 | CTX-1 | - | - |
| 2003 | CP1037(10) | Mexico | O1 Ogawa | + | - | - | *-* | - | - | - | - |
| 2003 | 4113 | Vietnam | O1 Inaba | + | - | :CTX: | *ctxB1* | CTX-1 | CTX-1 | RS1-cla | CTX-cla |
| 2003 | Nep-21106 | Nepal | O1 Ogawa | + | :TLC: :ctx: rstC rstA_rstR | - | *ctxB1* | CTX-1 | - | RS1-ET | CTX-1 |
| 2003 | Nep-21113 | Nepal | O1 Ogawa | + | :TLC: :ctx: rstC rstA_rstR | - | *ctxB1* | CTX-1 | - | RS1-ET | CTX-1 |
| 2003 | CISM_196 | Mozambique | O1 Ogawa | + | :TLC: :RS1:_:CTX: | - | *ctxB1* | CTX-1 | CTX-1 | RS1-ET | CTX-1 |
| 2003 | CISM_191 | Mozambique | O1 Ogawa | + | :TLC: :RS1:_:CTX: | - | *ctxB1* | CTX-1 | CTX-1 | RS1-ET | CTX-1 |
| 2003 | CISM_188 | Mozambique | O1 Ogawa | + | :TLC: :RS1:_:CTX: | - | *ctxB1* | CTX-1 | CTX-1 | RS1-ET | CTX-1 |
| 2003 | CISM_189 | Mozambique | O1 Ogawa | + | :TLC: :RS1:_:CTX: | - | *ctxB1* | CTX-1 | CTX-1 | RS1-ET | CTX-1 |
| 2003 | CISM_179 | Mozambique | O1 Ogawa | + | :TLC: :RS1:_:CTX: | - | *ctxB1* | CTX-1 | CTX-1 | RS1-ET | CTX-1 |
| 2003 | CISM_178 | Mozambique | O1 Ogawa | + | :TLC: :RS1:_:CTX: | - | *ctxB1* | CTX-1 | CTX-1 | RS1-ET | CTX-1 |
| 2003 | CISM_152 | Mozambique | O1 Ogawa | + | :TLC: :RS1:_:CTX: | - | *ctxB1* | CTX-1 | CTX-4\|RS1-ET | RS1-ET | CTX-1 |
| 2003 | CISM_154 | Mozambique | O1 Ogawa | + | :TLC: :RS1:_:CTX: | - | *ctxB1* | CTX-1 | RS1-ET\|CTX-4 | RS1-ET | CTX-1 |
| 2003 | CISM_151 | Mozambique | O1 Ogawa | + | :TLC: :RS1:_:CTX: | - | *ctxB1* | CTX-1 | CTX-6\|RS1-ET | RS1-ET | CTX-1 |
| 2003 | CISM_153 | Mozambique | O1 Ogawa | + | :TLC: :RS1:_:CTX: | - | *ctxB1* | CTX-1 | RS1-ET\|CTX-4 | RS1-ET | CTX-1 |
| 2003 | CISM_147 | Mozambique | O1 Ogawa | + | :TLC: :RS1:_:CTX: | - | *ctxB1* | CTX-1 | CTX-1 | RS1-ET | CTX-1 |
| 2003 | CISM_146 | Mozambique | O1 Ogawa | + | :TLC:_:RS1:_:CTX: | - | *ctxB1* | CTX-1 | CTX-1 | RS1-ET | CTX-1 |
| 2003 | CISM_134 | Mozambique | O1 Ogawa | + | :TLC: :RS1:_:CTX: | - | *ctxB1* | CTX-1 | CTX-1 | RS1-ET | CTX-1 |
| 2003 | CISM_122 | Mozambique | O1 Ogawa | + | :TLC: :RS1:_:CTX: | - | *ctxB1* | CTX-1 | RS1-ET\|CTX-4 | RS1-ET | CTX-1 |
| 2003 | CISM_121 | Mozambique | O1 Ogawa | + | :TLC: :RS1:_:CTX: | - | *ctxB1* | CTX-1 | RS1-ET\|CTX-4 | RS1-ET | CTX-1 |
| 2003 | CISM_120 | Mozambique | O1 Ogawa | + | :TLC: :RS1:_:CTX: | - | *ctxB1* | CTX-1 | RS1-ET\|CTX-4 | RS1-ET | CTX-1 |
| 2003 | CISM_105 | Mozambique | O1 Ogawa | + | :TLC: :RS1:_:CTX: | - | *ctxB1* | CTX-1 | CTX-1 | RS1-ET | CTX-1 |
| 2003 | CISM_101 | Mozambique | O1 Ogawa | + | :TLC: :RS1:_:CTX: | - | *ctxB1* | CTX-1 | CTX-4\|RS1-ET | RS1-ET | CTX-1 |
| 2003 | CISM_100 | Mozambique | O1 Ogawa | + | :TLC: :RS1:_:CTX: | - | *ctxB1* | CTX-1 | CTX-1 | RS1-ET | CTX-1 |
| 2003 | CISM_0091 | Mozambique | O1 Ogawa | + | :TLC: :RS1:_:CTX: | - | *ctxB1* | CTX-1 | CTX-1 | RS1-ET | CTX-1 |
| 2003 | CISM_0079 | Mozambique | O1 Ogawa | + | :TLC: :RS1:_:CTX: | - | *ctxB1* | CTX-1 | RS1-ET\|CTX-4 | RS1-ET | CTX-1 |
| 2003 | CISM_0074 | Mozambique | O1 Ogawa | + | :TLC:_:RS1:_:CTX: | - | *ctxB1* | CTX-1 | CTX-1 | RS1-ET | CTX-1 |
| 2003 | FC1105 | India | O139 | + | :TLC: rstA_rstB rstR rstC ace ace cep rstR ctxB_ctxA | - | *ctxB3* | CTX-USGulf | CTX-2 | CTX-1 | CTX-1\|CTX-cla |
| 2003 | M1425 | Russia | O1 Ogawa | + | - | - | *-* | - | - | - | - |
| 2004 | CP1041(14) | Zambia | O1 Ogawa | + | :TLC:_:CTX: | - | *ctxB1* | CTX-1 | CTX-1 | RS1-ET | CTX-1 |
| 2004 | CP1040(13) | Zambia | O1 Ogawa | + | :TLC: rstA_rstR rstC :ctx: | - | *ctxB1* | CTX-1 | - | RS1-ET | CTX-1 |
| 2004 | B33 | Mozambique | O1 Ogawa | + | - | :CTX: | *ctxB1* | CTX-1 | CTX-1 | RS1-cla | CTX-cla |
| 2004 | 4121 | Vietnam | O1 Inaba | + | - | :CTX: | *ctxB1* | CTX-1 | CTX-1 | RS1-cla | CTX-cla |
| 2004 | 4322 | India | NA | + | :TLC: rstA_rstR ctxA_zot_ace_orfU_cep rstC | - | *-* | CTX-1 | - | RS1-ET | CTX-1 |
| 2004 | 4339 | India | O1 Ogawa | + | :TLC: :RS1: :CTX: | - | *ctxB1* | CTX-1 | CTX-6\|RS1-ET | RS1-ET | CTX-1 |
| 2004 | MBRN14 | India | O1 Ogawa | + | - | - | *-* | - | - | - | - |
| 2004 | MBN17 | India | O1 Inaba | + | :TLC: :CTX: rstC_rstR_rstA_rstB | - | *ctxB1* | CTX-1 | CTX-5\|CTX-1 | RS1-ET | CTX-1 |
| 2004 | VC35 | Malaysia | O1 Inaba | - | - | - | *-* | - | - | - | - |
| 2004 | P-18748 | Russia | O1 Ogawa | - | - | - | *-* | - | - | - | - |
| 2004 | Sa5Y | United States | NA | - | - | - | *-* | - | - | - | - |
| 2005 | FJ147 | China | O1 Inaba | + | :TLC:_:TLC:_:CTX:_:RS1: | - | *ctxB1* | CTX-1 | RS1-ET\|CTX-1 | RS1-ET | CTX-1 |
| 2005 | RND18826 | Russia | O1 Inaba | + | :TLC: :ctx: rstC rstB_rstA_rstR | - | *ctxB1* | CTX-1 | RS1-ET | RS1-ET | CTX-1 |
| 2005 | 4519 | India | O1 Inaba | + | :TLC: :CTX: rstC | - | *ctxB7* | CTX-1 | CTX-1 | RS1-ET | CTX-1 |
| 2005 | 6193 | Kenya | O1 Inaba | + | :TLC: :CTX: rstB_rstA_rstR_rstC | - | *ctxB1* | CTX-1 | CTX-1\|RS1-ET | RS1-ET | CTX-1 |
| 2005 | 1362 | Mozambique | O1 Ogawa | + | - | :CTX: | *ctxB1* | CTX-1 | CTX-1 | RS1-cla | CTX-cla |
| 2005 | 6191 | Kenya | O1 var Inaba | + | rstC rstA_rstR :TLC: :ctx: | - | *ctxB1* | CTX-1 | - | RS1-ET | CTX-1 |
| 2005 | 6215 | Kenya | O1 Inaba | + | :CTX: rstC_rstR_rstA_rstB | :TLC: | *ctxB1* | CTX-1 | CTX-1\|CTX-5 | RS1-ET | CTX-1 |
| 2005 | 1346 | Mozambique | O1 Ogawa | + | rstC | :CTX: | *ctxB1* | CTX-1 | CTX-1 | RS1-cla | CTX-cla |
| 2005 | 1627 | Mozambique | O1 Ogawa | + | rstC_rstB | :CTX: | *ctxB1* | CTX-1 | CTX-2\|CTX-1 | RS1-cla | CTX-cla |
| 2005 | P-18785 | Russia | O1 Ogawa | + | :TLC: | - | *-* | - | - | - | - |
| 2005 | CISM_0008 | Mozambique | O1 Ogawa | + | :TLC: :RS1:_:CTX: | - | *ctxB1* | CTX-1 | CTX-4\|RS1-ET | RS1-ET | CTX-1 |
| 2005 | P-18778 | Russia | O1 Ogawa | + | :TLC: | - | *-* | - | - | - | - |
| 2006 | P18899 | Russia | O1 Inaba | + | :TLC: :ctx: rstC rstB_rstA_rstR | - | *ctxB1* | CTX-1 | RS1-ET | RS1-ET | CTX-1 |
| 2006 | P18899-D | Russia | O1 Inaba | + | - | - | *-* | - | - | - | - |
| 2006 | RND18899 | Russia | O1 Inaba | + | - | - | *-* | - | - | - | - |
| 2006 | 4642 | India | NA | + | :TLC:_rstR_rstA_rstB_cep_orfU_ace_zot rstC | - | *-* | CTX-1 | CTX-1 | RS1-ET | CTX-1 |
| 2006 | A488(1) | Bangladesh | O1 Ogawa | + | :TLC:_:TLC: :CTX: rstC_rstR_rstA | - | *ctxB1* | CTX-1 | CTX-1 | RS1-ET | CTX-1 |
| 2006 | 4656 | India | O1 Ogawa | + | :TLC: :CTX: rstC | - | *ctxB1* | CTX-1 | CTX-1 | RS1-ET | CTX-1 |
| 2006 | 4488 | India | NA | + | :TLC: :CTX: :RS1: | - | *ctxB1* | CTX-1 | RS1-ET\|CTX-1 | RS1-ET | CTX-1 |
| 2006 | A488(2) | Bangladesh | O1 Ogawa | + | :TLC: rstC rstA_rstR :ctx: | - | *ctxB1* | CTX-1 | - | RS1-ET | CTX-1 |
| 2006 | L15 | Sweden | Oa6 | - | - | - | *-* | - | - | - | - |
| 2006 | ICDC-VC661 | China | O139 | + | :TLC:_:TLC:_:CTX:_cep_orfU_ace_zot_cep_orfU_ace_zot | zot_ace_orfU_cep | *ctxB3* | CTX-1\|CTX-USGulf | CTX-1 | CTX-1 | CTX-1 |
| 2006 | 11116 | Sweden | Oa6 | - | - | - | *-* | - | - | - | - |
| 2007 | 6194 | Kenya | O1 Inaba | + | :TLC: :ctx: :RS1: | - | *ctxB1* | CTX-1 | RS1-ET | RS1-ET | CTX-1 |
| 2007 | 4551 | India | O1 Ogawa | + | :TLC: :ctx: rstC_rstR_rstA_rstB rstR_rstA | - | *ctxB1* | CTX-1 | RS1-ET | RS1-ET | CTX-1 |
| 2007 | 6214 | Kenya | NA | + | :TLC: rstR_rstA_rstB_orfU_ace_zot ctxB | - | *ctxB1* | - | CTX-1 | RS1-ET | CTX-1 |
| 2007 | 4538 | India | O1 Inaba | + | :TLC: :CTX: rstC_rstR_rstA | - | *ctxB7* | CTX-1 | CTX-1 | RS1-ET | CTX-1 |
| 2007 | 4646 | India | O1 Ogawa | + | :TLC: :CTX: rstC_rstR_rstA_rstB | - | *ctxB1* | CTX-1 | CTX-1\|RS1-ET | RS1-ET | CTX-1 |
| 2007 | 6210 | Kenya | O1 Inaba | + | :TLC: :CTX: rstC_rstR_rstA_rstB | - | *ctxB1* | CTX-1 | CTX-1\|RS1-ET | RS1-ET | CTX-1 |
| 2007 | A487(1) | Bangladesh | O1 Inaba | + | :TLC: :CTX: rstA_rstR_rstC | - | *ctxB1* | CTX-1 | CTX-1 | RS1-ET | CTX-1 |
| 2007 | 6201 | Kenya | O1 Inaba | + | :TLC: :CTX: rstC | - | *ctxB1* | CTX-1 | CTX-1 | RS1-ET | CTX-1 |
| 2007 | 4593 | India | O1 Ogawa | + | :TLC: :ctx: rstA_rstR rstB_rstA_rstR_rstC | - | *ctxB1* | CTX-1 | CTX-5 | RS1-ET | CTX-1 |
| 2007 | 6212 | Kenya | NA | + | :TLC: :ctx: rstR_rstA | - | *ctxB1* | CTX-1 | - | RS1-ET | CTX-1 |
| 2007 | 4605 | India | O1 Ogawa | + | :TLC: :ctx: rstA_rstR rstC | - | *ctxB1* | CTX-1 | - | RS1-ET | CTX-1 |
| 2007 | 4552 | India | O1 Ogawa | + | :CTX: rstC_rstR_rstA_rstB | :TLC: | *ctxB1* | CTX-1 | RS1-ET\|CTX-1 | RS1-ET | CTX-1 |
| 2007 | 4600 | India | O1 Ogawa | + | :TLC: :CTX: rstC_rstR_rstA_rstB | - | *ctxB1* | CTX-1 | RS1-ET\|CTX-1 | RS1-ET | CTX-1 |
| 2007 | 4585 | India | O1 Ogawa | + | :TLC: :CTX: rstB_rstA_rstR_rstC | - | *ctxB1* | CTX-1 | RS1-ET\|CTX-1 | RS1-ET | CTX-1 |
| 2007 | 4122 | Vietnam | NA | + | :TLC: rstR_rstB zot | - | *-* | CTX-1 | CTX-5 | RS1-ET | CTX-1 |
| 2007 | 4623 | India | O1 Ogawa | + | :TLC: :RS1: | :CTX: | *ctxB1* | CTX-1 | RS1-ET\|CTX-6 | RS1-ET | CTX-1 |
| 2007 | 6197 | Kenya | NA | + | :TLC:_:RS1: | :CTX: | *ctxB1* | CTX-1 | RS1-ET\|CTX-1 | RS1-ET | CTX-1 |
| 2007 | 4536 | India | O1 Ogawa | + | :TLC: rstC_rstB :CTX: | - | *ctxB1* | CTX-1 | RS1-ET\|CTX-1 | RS1-ET | CTX-1 |
| 2007 | PIC018 | Bangladesh | O1 Inaba | + | :TLC: :CTX: rstC_rstR_rstA_rstB | - | *ctxB1* | CTX-1 | CTX-5\|CTX-1 | RS1-ET | CTX-1 |
| 2008 | MS6 | Myanmar | O1 Ogawa | + | :TLC:_:TLC:_:CTX:_:CTX:_rstC | - | *ctxB3* | CTX-1 | CTX-1 | CTX-1 | CTX-1 |
| 2008 | CP1030(3) | Mexico | O1 Inaba | + | :TLC: ctxB_ctxA_zot_ace cep_rstB_rstA_rstR | - | *ctxB3* | CTX-1 | CTX-5 | RS1-ET | CTX-1 |
| 2008 | VC4370 | Malaysia | O139 | + | :TLC:_:CTX: | - | *ctxB3* | CTX-1 | CTX-1 | CTX-1 | CTX-1 |
| 2008 | DL4211 | United States | Oa7 | - | - | - | *-* | - | - | - | - |
| 2008 | DL4215 | United States | Oa8 | - | - | - | *-* | - | - | - | - |
| 2008 | CISM_300205 | Mozambique | NA | + | TLC4 TLC2 | - | *-* | - | - | - | - |
| 2008 | CISM_300043 | Mozambique | O1 Ogawa | + | :TLC: :RS1:_:CTX: | - | *ctxB1* | CTX-1 | CTX-5\|RS1-ET | RS1-ET | CTX-1 |
| 2008 | CISM_300215 | Mozambique | O1 Ogawa | + | :TLC: :RS1:_:CTX: | - | *ctxB1* | CTX-1 | RS1-ET\|CTX-1 | RS1-ET | CTX-1 |
| 2008 | CISM_300209 | Mozambique | O1 Ogawa | + | :TLC: :RS1:_:CTX: | - | *ctxB1* | CTX-1 | CTX-4\|RS1-ET | RS1-ET | CTX-1 |
| 2008 | CISM_300208 | Mozambique | O1 Ogawa | + | :TLC: :RS1:_:CTX: | - | *ctxB1* | CTX-1 | CTX-4\|RS1-ET | RS1-ET | CTX-1 |
| 2009 | 2009V-1046 | United States | O1 Ogawa | + | :TLC: :ctx: rstC rstR_rstA | - | *ctxB1* | CTX-1 | - | RS1-ET | CTX-1 |
| 2009 | 2009V-1085 | United States | O1 Ogawa | + | :TLC: :ctx: rstR_rstA rstC | - | *ctxB7* | CTX-1 | - | RS1-ET | CTX-1 |
| 2009 | 2009V-1096 | United States | O1 Inaba | + | :TLC: :ctx: rstR_rstA rstC | - | *ctxB7* | CTX-1 | - | RS1-ET | CTX-1 |
| 2009 | 2009V-1116 | United States | O1 Ogawa | + | :TLC: rstC :ctx: rstR_rstA | - | *ctxB1* | CTX-1 | - | RS1-ET | CTX-1 |
| 2009 | 2009V-1131 | United States | NA | + | :TLC: rstC :ctx: rstR_rstA | - | *ctxB7* | CTX-1 | - | RS1-ET | CTX-1 |
| 2009 | 2011EL-1137 | Haiti | O1 Ogawa | + | :TLC: :ctx: rstR_rstA_rstB | - | *ctxB1* | CTX-1 | RS1-ET | RS1-ET | CTX-1 |
| 2009 | 7687 | Kenya | O1 Inaba | + | :TLC: :CTX: rstC_rstR_rstA_rstB | - | *ctxB1* | CTX-1 | CTX-5\|CTX-1 | RS1-ET | CTX-1 |
| 2009 | IDHO1_726 | India | O1 Ogawa | + | :TLC: :ctx: rstC_rstR_rstA rstR_rstA | - | *ctxB7* | CTX-1 | - | RS1-ET | CTX-1 |
| 2009 | 4784 | Tanzania | O1 Ogawa | + | :TLC: rstC_rstR_rstA_rstB :CTX: | - | *ctxB1* | CTX-1 | RS1-ET\|CTX-1 | RS1-ET | CTX-1 |
| 2009 | 7686 | Kenya | O1 var Inaba | + | :TLC: :ctx: rstR_rstA rstC | - | *ctxB1* | CTX-1 | - | RS1-ET | CTX-1 |
| 2009 | 7685 | Kenya | O1 var Inaba | + | :TLC: :RS1: :ctx:_rstB | - | *ctxB1* | CTX-1 | RS1-ET\|CTX-6 | RS1-ET | CTX-1 |
| 2009 | VC1761 | Malaysia | O1 Ogawa | + | :TLC: rstR_rstA_rstB rstC :ctx: | - | *ctxB1* | CTX-1 | RS1-ET | RS1-ET | CTX-1 |
| 2009 | YB4G06 | United States | Ob8 | - | - | - | *-* | - | - | - | - |
| 2009 | YB1A01 | United States | Ob6 | - | - | - | *-* | - | - | - | - |
| 2009 | YB3G04 | United States | Ob8 | - | - | - | *-* | - | - | - | - |
| 2009 | YB7A06 | United States | Ob6 | - | - | - | *-* | - | - | - | - |
| 2009 | YB4C07 | United States | Ob7 | - | - | - | *-* | - | - | - | - |
| 2009 | YB2A06 | United States | Ob8 | - | - | - | *-* | - | - | - | - |
| 2009 | YB5A06 | United States | Ob6 | - | - | - | *-* | - | - | - | - |
| 2009 | YB3B05 | United States | Ob7 | - | - | - | *-* | - | - | - | - |
| 2009 | YB4G05 | United States | Oc2 | - | - | - | *-* | - | - | - | - |
| 2009 | YB4H02 | United States | Ob8 | - | - | - | *-* | - | - | - | - |
| 2009 | YB4B03 | United States | Oc2 | - | - | - | *-* | - | - | - | - |
| 2009 | YB8E08 | United States | Ob7 | - | - | - | *-* | - | - | - | - |
| 2009 | YB2G01 | United States | Ob8 | - | - | - | *-* | - | - | - | - |
| 2009 | VC53 | United States | NA | - | - | - | *-* | - | - | - | - |
| 2009 | VC56 | United States | NA | - | - | - | *-* | - | - | - | - |
| 2009 | S12 | Australia | Ob5 | - | - | - | *-* | - | - | - | - |
| 2009 | CISM_302015 | Mozambique | O1 Ogawa | + | :TLC:_:RS1:_:CTX: | - | *ctxB1* | CTX-1 | CTX-1 | RS1-ET | CTX-1 |
| 2009 | CISM_302029 | Mozambique | O1 Ogawa | + | :TLC: :RS1:_:CTX: | - | *ctxB1* | CTX-1 | CTX-1 | RS1-ET | CTX-1 |
| 2009 | OYP2D07 | United States | Oa9 | - | - | - | *-* | - | - | - | - |
| 2009 | OYP6D06 | United States | Ob2 | - | - | - | *-* | - | - | - | - |
| 2009 | OYP7C09 | United States | Ob6 | - | - | - | *-* | - | - | - | - |
| 2009 | OYP2A12 | United States | Ob9 | - | - | - | *-* | - | - | - | - |
| 2009 | OYP6F10 | United States | Ob4 | - | - | - | *-* | - | - | - | - |
| 2009 | OYP6E07 | United States | Ob3 | + | - | - | *-* | - | - | - | - |
| 2009 | OYP8F12 | United States | Ob8 | - | - | - | *-* | - | - | - | - |
| 2009 | OYP3F10 | United States | Oc1 | - | - | - | *-* | - | - | - | - |
| 2009 | OYP5F10 | United States | Ob1 | - | - | - | *-* | - | - | - | - |
| 2009 | OYP8C06 | United States | Ob8 | - | - | - | *-* | - | - | - | - |
| 2010 | 2010EL-1786 | Haiti | O1 Ogawa | + | :TLC:_:RS1:_:CTX: | - | *ctxB7* | CTX-1 | CTX-6\|RS1-ET | RS1-ET | CTX-1 |
| 2010 | H1 | Haiti | O1 Ogawa | + | :TLC:_:TLC:_:RS1:_:CTX: | - | *ctxB7* | CTX-1 | CTX-6\|RS1-ET | RS1-ET | CTX-1 |
| 2010 | HC-49A2 | Haiti | O1 Ogawa | + | :TLC: :ctx: | - | *ctxB7* | CTX-1 | - | - | - |
| 2010 | RND19188 | Russia | O1 Ogawa | + | :TLC: rstA_rstB rstC rstR cep rstR ctxA_ctxB zot_ace | - | *ctxB7* | CTX-cla | CTX-1 | RS1-ET | CTX-1\|CTX-cla |
| 2010 | RND19191 | Russia | NA | + | :TLC: rstR_rstA_rstB orfU_ace_zot_ctxA_ctxB rstC cep | rstR | *ctxB7* | CTX-1 | CTX-1 | CTX-1 | CTX-cla\|CTX-1 |
| 2010 | HC-06A1 | Haiti | O1 Ogawa | + | :TLC: rstC rstB_rstA_rstR :ctx: | - | *ctxB7* | CTX-1 | RS1-ET | RS1-ET | CTX-1 |
| 2010 | HC-23A1 | Haiti | O1 Ogawa | + | :TLC: :ctx:_rstB rstC_rstB | - | *ctxB7* | CTX-1 | RS1-ET\|CTX-6 | - | - |
| 2010 | HC-28A1 | Haiti | O1 Ogawa | + | :TLC: :ctx: rstC rstB_rstA_rstR | - | *ctxB7* | CTX-1 | RS1-ET | RS1-ET | CTX-1 |
| 2010 | HC-43A1 | Haiti | O1 Ogawa | + | :TLC: :RS1:_TLC1 :ctx:_rstB | - | *ctxB7* | CTX-1 | CTX-6\|RS1-ET | RS1-ET | CTX-1 |
| 2010 | HC-1A2 | Haiti | Oc5 | - | - | - | *-* | - | - | - | - |
| 2010 | HC-61A2 | Haiti | Oc5 | - | - | - | *-* | - | - | - | - |
| 2010 | HC-46B1 | Haiti | Oc4 | - | - | - | *-* | - | - | - | - |
| 2010 | L-3226 | Russia | NA | + | :TLC: :ctx: rstB_rstA_rstR | - | *ctxB1* | CTX-1 | CTX-1 | RS1-ET | CTX-1 |
| 2010 | CP1042(15) | Thailand | O1 Ogawa | + | :ctx:_rstB rstC_rstB | - | *ctxB1* | CTX-1 | CTX-1\|RS1-ET | - | - |
| 2010 | CP1048(21) | Bangladesh | O1 Ogawa | + | :TLC: :ctx:_rstB rstC | - | *ctxB7* | CTX-1 | CTX-2 | RS1-ET | - |
| 2010 | HC-20A2 | Haiti | O1 Ogawa | + | :TLC:_:CTX: | - | *ctxB7* | CTX-1 | CTX-6 | RS1-ET | CTX-1 |
| 2010 | HC-43B1 | Haiti | Oc4 | - | - | - | *-* | - | - | - | - |
| 2010 | HC-46A1 | Haiti | O1 Ogawa | + | :TLC: :TLC: :ctx:_rstB rstC_rstB | - | *ctxB7* | CTX-1 | CTX-6\|RS1-ET | - | - |
| 2010 | HE-25 | Haiti | Oc6 | - | - | - | *-* | - | - | - | - |
| 2010 | HE-45 | Haiti | Oc7 | - | - | - | *-* | - | - | - | - |
| 2010 | HC-38A1 | Haiti | NA | + | :TLC: :TLC: :ctx: rstC rstB_rstA_rstR | - | *ctxB7* | CTX-1 | RS1-ET | RS1-ET | CTX-1 |
| 2010 | HC-19A1 | Haiti | O1 Ogawa | + | :TLC: rstB_rstA_rstR rstC :ctx: | - | *ctxB7* | CTX-1 | RS1-ET | RS1-ET | CTX-1 |
| 2010 | HC-21A1 | Haiti | O1 Ogawa | + | :TLC: :ctx: rstB_rstA_rstR rstC | - | *ctxB7* | CTX-1 | RS1-ET | RS1-ET | CTX-1 |
| 2010 | HC-22A1 | Haiti | O1 Ogawa | + | :TLC: :TLC: :ctx: rstB_rstA_rstR rstC | - | *ctxB7* | CTX-1 | RS1-ET | RS1-ET | CTX-1 |
| 2010 | HC-32A1 | Haiti | O1 Ogawa | + | :TLC: :ctx: rstC rstB_rstA_rstR | - | *ctxB7* | CTX-1 | RS1-ET | RS1-ET | CTX-1 |
| 2010 | HC-33A2 | Haiti | O1 Ogawa | + | :TLC: :TLC: :ctx: rstC rstB_rstA_rstR | - | *ctxB7* | CTX-1 | RS1-ET | RS1-ET | CTX-1 |
| 2010 | HC-48B2 | Haiti | O1 Ogawa | + | :TLC: ctxB_ctxA rstC zot_ace_orfU_cep_rstB_rstA_rstR | - | *ctxB7* | CTX-1 | CTX-6 | RS1-ET | CTX-1 |
| 2010 | HC-50A2 | Haiti | NA | + | :TLC: zot_ace_orfU_cep_rstB_rstA_rstR ctxB_ctxA | - | *ctxB7* | CTX-1 | CTX-6 | RS1-ET | CTX-1 |
| 2010 | CP1050(23) | Bangladesh | NA | + | :TLC: rstA_rstR :ctx: rstC | - | *ctxB7* | CTX-1 | - | RS1-ET | CTX-1 |
| 2010 | HC-39A1 | Haiti | NA | + | :TLC: rstC rstB_rstA_rstR :ctx: | - | *ctxB7* | CTX-1 | RS1-ET | RS1-ET | CTX-1 |
| 2010 | HC-41A1 | Haiti | NA | + | :TLC: rstC :ctx: rstB_rstA_rstR | - | *ctxB7* | CTX-1 | RS1-ET | RS1-ET | CTX-1 |
| 2010 | HC-42A1 | Haiti | NA | + | :TLC: rstB_rstA_rstR :ctx: rstC | - | *ctxB7* | CTX-1 | RS1-ET | RS1-ET | CTX-1 |
| 2010 | HC-47A1 | Haiti | NA | + | :TLC: rstB_rstA_rstR rstC :ctx: | - | *ctxB7* | CTX-1 | RS1-ET | RS1-ET | CTX-1 |
| 2010 | HC-56A2 | Haiti | NA | + | :TLC: :ctx: rstC rstB_rstA_rstR | - | *ctxB7* | CTX-1 | RS1-ET | RS1-ET | CTX-1 |
| 2010 | HC-57A2 | Haiti | O1 Ogawa | + | :TLC: :ctx: rstB_rstA_rstR rstC | - | *ctxB7* | CTX-1 | RS1-ET | RS1-ET | CTX-1 |
| 2010 | HC-81A2 | Haiti | O1 Ogawa | + | :TLC: :ctx: rstB_rstA_rstR rstC | - | *ctxB7* | CTX-1 | RS1-ET | RS1-ET | CTX-1 |
| 2010 | HC-64A1 | Haiti | O1 Ogawa | + | :TLC: :TLC: :ctx: rstB_rstA_rstR rstC | - | *ctxB7* | CTX-1 | RS1-ET\|CTX-5\|RS1-ET | RS1-ET | CTX-1 |
| 2010 | HC-65A1 | Haiti | O1 Ogawa | + | :TLC: :ctx: rstB_rstA_rstR rstC | - | *ctxB7* | CTX-1 | RS1-ET\|CTX-5\|RS1-ET | RS1-ET | CTX-1 |
| 2010 | HC-67A1 | Haiti | O1 Ogawa | + | :TLC: :ctx: rstB_rstA_rstR rstC | - | *ctxB7* | CTX-1 | CTX-6\|RS1-ET\|RS1-cla | RS1-ET | CTX-1 |
| 2010 | HC-68A1 | Haiti | O1 Ogawa | + | :TLC: :ctx: rstB_rstA_rstR rstC | - | *ctxB7* | CTX-1 | CTX-6\|RS1-ET\|RS1-cla | RS1-ET | CTX-1 |
| 2010 | HC-71A1 | Haiti | O1 Ogawa | + | :TLC: :ctx: rstB_rstA_rstR rstC | - | *ctxB7* | CTX-1 | CTX-6\|RS1-ET\|RS1-cla | RS1-ET | CTX-1 |
| 2010 | HC-72A2 | Haiti | O1 Ogawa | + | :TLC: :ctx: rstB_rstA_rstR rstC | - | *ctxB7* | CTX-1 | CTX-6\|RS1-ET\|RS1-cla | RS1-ET | CTX-1 |
| 2010 | HC-7A1 | Haiti | O1 Ogawa | + | :TLC: :TLC:_:CTX: | - | *ctxB7* | CTX-1 | CTX-6 | RS1-ET | CTX-1 |
| 2010 | HC-80A1 | Haiti | O1 Ogawa | + | :TLC: :TLC: :ctx:_rstB rstB_rstA_rstR rstC_rstB | - | *ctxB7* | CTX-1 | RS1-ET\|RS1-cla\|CTX-6 | RS1-ET | CTX-1 |
| 2010 | HC-81A1 | Haiti | O1 Ogawa | + | :TLC: :TLC:_:CTX: | - | *ctxB7* | CTX-1 | CTX-6 | RS1-ET | CTX-1 |
| 2010 | HC-36A1 | ND | Oc5 | - | - | - | *-* | - | - | - | - |
| 2010 | 2010EL-1798 | Haiti | O1 Ogawa | + | :TLC: :ctx: rstR_rstA_rstB | - | *ctxB7* | CTX-1 | RS1-ET | RS1-ET | CTX-1 |
| 2010 | 2010EL-1792 | Haiti | O1 Ogawa | + | :TLC: :ctx: rstB_rstA_rstR rstC | - | *ctxB7* | CTX-1 | RS1-ET | RS1-ET | CTX-1 |
| 2010 | 2010EL-1749 | Cameroon | O1 Ogawa | + | :TLC: :ctx: rstR rstB | - | *ctxB7* | CTX-1 | RS1-ET | - | CTX-1 |
| 2010 | 2010EL-1961 | Haiti | NA | + | :TLC: :ctx: rstR_rstA_rstB | - | *ctxB7* | CTX-1 | RS1-ET | RS1-ET | CTX-1 |
| 2010 | 2010EL-2010H | Haiti | O1 Ogawa | + | :TLC: :ctx: rstR_rstA_rstB | - | *ctxB7* | CTX-1 | RS1-ET | RS1-ET | CTX-1 |
| 2010 | 2010EL-2010N | Haiti | O1 Ogawa | + | :TLC: :ctx: rstR_rstA_rstB | - | *ctxB7* | CTX-1 | RS1-ET | RS1-ET | CTX-1 |
| 2010 | 2010V-1014 | Haiti | O1 Ogawa | + | :TLC: :ctx: rstR_rstA_rstB | - | *ctxB1* | CTX-1 | CTX-1 | RS1-ET | CTX-1 |
| 2010 | 2011EL-1089 | Haiti | O1 Ogawa | + | :TLC: :ctx: rstR_rstA_rstB | - | *ctxB7* | CTX-1 | RS1-ET | RS1-ET | CTX-1 |
| 2010 | 2010AA-143 | Haiti | O1 Ogawa | + | :TLC: :ctx: rstB_rstA_rstR rstC | - | *ctxB7* | CTX-1 | RS1-ET | RS1-ET | CTX-1 |
| 2010 | 2010AA-142 | Haiti | O1 Ogawa | + | :TLC: :ctx: rstB_rstA_rstR rstC | - | *ctxB7* | CTX-1 | RS1-ET | RS1-ET | CTX-1 |
| 2010 | 2010AA-144 | Haiti | O1 Ogawa | + | :TLC: :ctx: rstB_rstA_rstR rstC | - | *ctxB7* | CTX-1 | RS1-ET | RS1-ET | CTX-1 |
| 2010 | 2010AA-145 | Haiti | O1 Ogawa | + | :TLC: :ctx: rstC rstB_rstA_rstR | - | *ctxB7* | CTX-1 | RS1-ET | RS1-ET | CTX-1 |
| 2010 | 2010AA-146 | Haiti | O1 Ogawa | + | :TLC: :ctx: rstC rstB_rstA_rstR | - | *ctxB7* | CTX-1 | RS1-ET | RS1-ET | CTX-1 |
| 2010 | 2010AA-147 | Haiti | O1 Ogawa | + | :TLC: :ctx: rstC rstR_rstA_rstB | - | *ctxB7* | CTX-1 | RS1-ET | RS1-ET | CTX-1 |
| 2010 | 2010AA-148 | Haiti | O1 Ogawa | + | :TLC: :ctx: rstA_rstR rstC | - | *ctxB7* | CTX-1 | - | RS1-ET | CTX-1 |
| 2010 | 2010AA-150 | Haiti | O1 Ogawa | + | :TLC: :ctx: rstC rstR_rstA_rstB | - | *ctxB7* | CTX-1 | RS1-ET | RS1-ET | CTX-1 |
| 2010 | 2010AA-151 | Haiti | O1 Ogawa | + | :TLC: :ctx: rstC rstR_rstA_rstB | - | *ctxB7* | CTX-1 | RS1-ET | RS1-ET | CTX-1 |
| 2010 | RND19187 | Russia | O1 Ogawa | + | :TLC: :ctx: rstC rstB_rstA_rstR | - | *ctxB7* | CTX-1 | CTX-1 | RS1-ET | CTX-1 |
| 2010 | TSY216 | Thailand | O1 Ogawa | + | :TLC:_:TLC:_:RS1:_:CTX: | - | *ctxB1* | CTX-1 | CTX-1\|RS1-ET | RS1-ET | CTX-1 |
| 2010 | EM-1543 | Bangladesh | O1 Ogawa | + | :TLC: :RS1:_:CTX: | - | *ctxB1* | CTX-1 | RS1-ET\|CTX-4 | RS1-ET | CTX-1 |
| 2010 | NHCC-011 | Bangladesh | O1 Ogawa | + | :TLC: :RS1:_:CTX: | - | *ctxB7* | CTX-1 | CTX-1 | RS1-ET | CTX-1 |
| 2010 | NHCC-04 | Bangladesh | O1 Ogawa | + | :TLC: :RS1:_:CTX: | - | *ctxB1* | CTX-1 | RS1-ET\|CTX-1 | RS1-ET | CTX-1 |
| 2010 | NHCC-048 | Bangladesh | O1 Ogawa | + | :TLC: :RS1:_:CTX: | - | *ctxB7* | CTX-1 | CTX-1 | RS1-ET | CTX-1 |
| 2010 | NHCC-021 | Bangladesh | O1 Ogawa | + | :TLC: :RS1:_:CTX: | - | *ctxB7* | CTX-1 | CTX-1 | RS1-ET | CTX-1 |
| 2010 | NHCC-042 | Bangladesh | O1 Ogawa | + | :TLC: :RS1:_:CTX: | - | *ctxB7* | CTX-1 | CTX-1 | RS1-ET | CTX-1 |
| 2010 | NHCC-019 | Bangladesh | NA | + | :TLC: :RS1:_:CTX: | - | *ctxB7* | CTX-1 | CTX-1 | RS1-ET | CTX-1 |
| 2010 | NHCC-05 | Bangladesh | O1 Ogawa | + | :TLC: :RS1:_:CTX: | - | *ctxB7* | CTX-1 | CTX-4\|RS1-ET | RS1-ET | CTX-1 |
| 2010 | NHCM-01 | Bangladesh | O1 Ogawa | + | :TLC: :RS1:_:CTX: | - | *ctxB1* | CTX-1 | CTX-4\|RS1-ET | RS1-ET | CTX-1 |
| 2010 | NHCC-068 | Bangladesh | O1 Ogawa | + | :TLC: :RS1:_:CTX: | - | *ctxB7* | CTX-1 | CTX-4\|RS1-ET | RS1-ET | CTX-1 |
| 2010 | EM-1542 | Bangladesh | O1 Ogawa | + | :TLC: :RS1:_:CTX: | - | *ctxB1* | CTX-1 | CTX-4\|RS1-ET | RS1-ET | CTX-1 |
| 2010 | HC-17A1 | Haiti | O1 Ogawa | + | :TLC: :ctx: rstC rstB_rstA_rstR | - | *ctxB7* | CTX-1 | RS1-ET | RS1-ET | CTX-1 |
| 2010 | HC-41B1 | Haiti | Oc4 | - | - | - | *-* | - | - | - | - |
| 2010 | HC-62A1 | Haiti | O1 Ogawa | + | :TLC: :TLC: :ctx: rstB_rstA_rstR rstC | - | *ctxB7* | CTX-1 | RS1-ET | RS1-ET | CTX-1 |
| 2010 | HC-77A1 | Haiti | O1 Ogawa | + | :TLC: :ctx: rstB_rstA_rstR rstC | - | *ctxB7* | CTX-1 | RS1-ET | RS1-ET | CTX-1 |
| 2010 | HE-40 | Haiti | Oc3 | - | - | - | *-* | - | - | - | - |
| 2010 | HC-02C1 | Haiti | Oc5 | - | - | - | *-* | - | - | - | - |
| 2010 | HC-59B1 | Haiti | Oc5 | - | - | - | *-* | - | - | - | - |
| 2010 | HC-44C1 | Haiti | Oc4 | - | - | - | *-* | - | - | - | - |
| 2010 | HC-37A1 | Haiti | O1 Ogawa | + | :TLC: :TLC: :ctx: rstB_rstA_rstR rstC | - | *ctxB7* | CTX-1 | RS1-ET | RS1-ET | CTX-1 |
| 2010 | HC-62B1 | Haiti | O1 Ogawa | + | :TLC: :ctx: :RS1: | - | *ctxB7* | CTX-1 | RS1-ET | RS1-ET | CTX-1 |
| 2010 | HC-17A2 | Haiti | O1 Ogawa | + | :TLC: :ctx: :RS1: | - | *ctxB7* | CTX-1 | RS1-ET | RS1-ET | CTX-1 |
| 2010 | HC-69A1 | Haiti | O1 Ogawa | + | :TLC: :ctx: :RS1: | - | *ctxB7* | CTX-1 | RS1-ET | RS1-ET | CTX-1 |
| 2010 | EC-0009 | Bangladesh | O1 Ogawa | + | :TLC: :ctx: rstA_rstR rstC | - | *ctxB7* | CTX-1 | - | RS1-ET | CTX-1 |
| 2010 | EC-0012 | Bangladesh | O1 Ogawa | + | :TLC: :ctx: rstA_rstR rstC | - | *ctxB7* | CTX-1 | - | RS1-ET | CTX-1 |
| 2010 | EDC-020 | Bangladesh | O1 Ogawa | + | :TLC: :ctx: rstC rstA_rstR | - | *ctxB7* | CTX-1 | - | RS1-ET | CTX-1 |
| 2010 | EDC-022 | Bangladesh | O1 Ogawa | + | :TLC: :ctx: rstC rstA_rstR | - | *ctxB1* | CTX-1 | - | RS1-ET | CTX-1 |
| 2010 | EM-1546 | Bangladesh | O1 Ogawa | + | :TLC: :ctx: rstC rstA_rstR | - | *ctxB7* | CTX-1 | - | RS1-ET | CTX-1 |
| 2010 | EM-1536 | Bangladesh | O1 Ogawa | + | :TLC: :CTX: rstB_rstA_rstR_rstC | - | *ctxB1* | CTX-1 | CTX-1\|RS1-ET | RS1-ET | CTX-1 |
| 2010 | NHCC-004A | Bangladesh | O1 Ogawa | + | :TLC: :TLC: :ctx: rstA_rstR rstC | - | *ctxB7* | CTX-1 | - | RS1-ET | CTX-1 |
| 2010 | NHCC-006C | Bangladesh | O1 Ogawa | + | :TLC: :ctx:_rstB rstA_rstR rstC_rstB | - | *ctxB7* | CTX-1 | RS1-ET\|CTX-1 | RS1-ET | CTX-1 |
| 2010 | NHCC-010F | Bangladesh | O1 Ogawa | + | :TLC: :ctx: rstC rstA_rstR | - | *ctxB7* | CTX-1 | - | RS1-ET | CTX-1 |
| 2010 | PCS-023 | Bangladesh | O1 Ogawa | + | :TLC: :ctx: rstC rstA_rstR | - | *ctxB7* | CTX-1 | - | RS1-ET | CTX-1 |
| 2010 | EC-051 | Bangladesh | O1 Ogawa | + | :TLC: :RS1: :ctx: | - | *ctxB3* | CTX-1 | RS1-ET | CTX-1 | CTX-1 |
| 2010 | CMR001 | Cameroon | NA | + | :TLC: zot_ace_orfU_cep_rstB ctxB rstB_rstC rstA_rstR | - | *ctxB7* | CTX-1 | CTX-1\|RS1-ET | RS1-ET | CTX-1 |
| 2010 | CMR004 | Cameroon | NA | + | :TLC: rstC rstR_rstA_cep_orfU_ace_zot | - | *-* | CTX-1 | - | RS1-ET | CTX-1 |
| 2010 | CMR007 | Cameroon | NA | + | :TLC: :RS1:_:ctx: | - | *ctxB7* | CTX-1 | CTX-1 | RS1-ET | CTX-1 |
| 2010 | CMR008 | Cameroon | O1 Ogawa | + | :TLC: rstB_:ctx: rstC_rstB rstR_rstA | - | *ctxB7* | CTX-1 | CTX-1\|RS1-ET | RS1-ET | CTX-1 |
| 2010 | CMR009 | Cameroon | O1 Ogawa | + | :TLC: rstR_rstA rstC | :ctx: | *ctxB7* | CTX-1 | - | RS1-ET | CTX-1 |
| 2010 | CMR010 | Cameroon | NA | + | :TLC: rstC rstR rstA ctxA cep_orfU_ace | - | *-* | CTX-1 | - | RS1-ET | CTX-1 |
| 2010 | CISM_1020234.0 | Mozambique | O1 Ogawa | + | :TLC: :RS1:_:CTX: | - | *ctxB1* | CTX-1 | RS1-ET\|CTX-4 | RS1-ET | CTX-1 |
| 2010 | CISM_1020231.9 | Mozambique | O1 Ogawa | + | :TLC: :RS1:_:CTX: | - | *ctxB1* | CTX-1 | CTX-4\|RS1-ET | RS1-ET | CTX-1 |
| 2010 | CISM_1020229.6 | Mozambique | O1 Ogawa | + | :TLC: :RS1:_:CTX: | - | *ctxB1* | CTX-1 | CTX-1 | RS1-ET | CTX-1 |
| 2010 | CISM_1019829.2 | Mozambique | O1 Ogawa | + | :TLC: :RS1:_:CTX: | - | *ctxB1* | CTX-1 | CTX-4\|RS1-ET | RS1-ET | CTX-1 |
| 2010 | CISM_1019828.5 | Mozambique | O1 Ogawa | + | :TLC: :RS1:_:CTX: | - | *ctxB1* | CTX-1 | RS1-ET\|CTX-4 | RS1-ET | CTX-1 |
| 2011 | CP1110 | United States | NA | + | rstB_rstA orfU_ace_zot rstR rstR ctxB_ctxA | - | *ctxB1* | - | CTX-USGulf | CTX-USGulf | CTX-O139\|CTX-USGulf |
| 2011 | CP1115 | United States | NA | + | rstR rstA_rstB rstC ctxA_ctxB orfU_ace_zot rstR | - | *ctxB1* | - | CTX-USGulf | CTX-USGulf | CTX-O139\|CTX-USGulf |
| 2011 | CP1111 | United States | NA | + | ctxA_ctxB rstR zot_ace_orfU rstC rstB_rstA rstR | - | *ctxB1* | - | CTX-USGulf | CTX-USGulf | CTX-O139\|CTX-USGulf |
| 2011 | CP1112 | United States | NA | + | ctxB_ctxA rstB_rstA_rstR zot_ace_orfU rstR | - | *ctxB1* | - | CTX-USGulf | CTX-USGulf | CTX-O139\|CTX-USGulf |
| 2011 | CP1113 | United States | NA | + | rstA_rstB rstR rstR zot_ace_orfU ctxB_ctxA | - | *ctxB1* | - | CTX-USGulf | CTX-USGulf | CTX-O139\|CTX-USGulf |
| 2011 | CP1114 | United States | NA | + | orfU_ace_zot rstR rstR ctxA_ctxB rstB | - | *ctxB1* | - | CTX-USGulf | - | CTX-O139\|CTX-USGulf |
| 2011 | CP1117 | United States | NA | + | orfU_ace_zot rstR rstA_rstB rstR ctxA_ctxB | - | *ctxB1* | - | CTX-USGulf | CTX-USGulf | CTX-O139\|CTX-USGulf |
| 2011 | CP1116 | United States | NA | + | rstR rstC zot_ace_orfU ctxB_ctxA rstB_rstA rstR | - | *ctxB1* | - | CTX-USGulf | CTX-USGulf | CTX-O139\|CTX-USGulf |
| 2011 | I-1471 | Russia | O1 var Inaba | + | :TLC: | - | *-* | - | - | - | - |
| 2011 | PhVC-326 | Philippines | O1 Ogawa | + | :TLC: rstC_rstB rstR :ctx:_rstB rstR | - | *ctxB1* | CTX-1 | RS1-ET\|CTX-1 | - | CTX-1\|CTX-cla |
| 2011 | PhVE-5 | Philippines | O1 Ogawa | + | :TLC: :ctx: rstR rstC rstR | - | *ctxB1* | CTX-1 | - | - | CTX-1\|CTX-cla |
| 2011 | PhVC-311 | Philippines | O1 Ogawa | + | :TLC: :ctx: rstR rstR rstA_rstB | - | *ctxB1* | CTX-1 | CTX-1 | CTX-1 | CTX-1\|CTX-cla |
| 2011 | YN2011004 | China | O1 Ogawa | + | :TLC: :ctx: rstA_rstR rstC | - | *ctxB1* | CTX-1 | - | RS1-ET | CTX-1 |
| 2011 | 2011V-1021 | Dominican Republic | O1 Ogawa | + | :TLC: :ctx: rstR_rstA_rstB | - | *ctxB7* | CTX-1 | RS1-ET | RS1-ET | CTX-1 |
| 2011 | 2011EL-301 | Russia | O1 Inaba | + | :TLC: rstC :ctx: rstR_rstA_rstB | - | *ctxB1* | CTX-1 | CTX-2 | RS1-ET | CTX-1 |
| 2011 | EM-1727 | Bangladesh | O1 Ogawa | + | :TLC: :ctx: rstC rstB_rstA_rstR | - | *ctxB3* | CTX-1 | CTX-5 | CTX-1 | CTX-1 |
| 2011 | EM-1626 | Bangladesh | O1 Ogawa | + | :TLC: :RS1:_:CTX: | - | *ctxB1* | CTX-1 | CTX-1\|RS1-ET | RS1-ET | CTX-1 |
| 2011 | NHCC-078 | Bangladesh | O1 Ogawa | + | :TLC: :RS1:_:CTX: | - | *ctxB7* | CTX-1 | RS1-ET\|CTX-4 | RS1-ET | CTX-1 |
| 2011 | EM-1652A | Bangladesh | O1 Ogawa | + | :TLC: :RS1:_:CTX: | - | *ctxB1* | CTX-1 | CTX-1\|RS1-ET | RS1-ET | CTX-1 |
| 2011 | EM-1688 | Bangladesh | O1 Ogawa | + | :TLC: :RS1:_:CTX: | - | *ctxB1* | CTX-1 | CTX-4\|RS1-ET | RS1-ET | CTX-1 |
| 2011 | EM-1654 | Bangladesh | O1 Ogawa | + | :TLC: :RS1:_:CTX: | - | *ctxB1* | CTX-1 | CTX-4\|RS1-ET | RS1-ET | CTX-1 |
| 2011 | EM-1690A | Bangladesh | O1 Ogawa | + | :TLC: :RS1:_:CTX: | - | *ctxB1* | CTX-1 | RS1-ET\|CTX-4 | RS1-ET | CTX-1 |
| 2011 | EM-1690 | Bangladesh | O1 Ogawa | + | :TLC: :RS1:_:CTX: | - | *ctxB1* | CTX-1 | CTX-1\|RS1-ET | RS1-ET | CTX-1 |
| 2011 | EM-1706 | Bangladesh | O1 Ogawa | - | :TLC: rstR rstB_rstA rstC | rstR :ctx: | *ctxB1* | CTX-1 | RS1-ET | CTX-2 | CTX-1\|CTX-cla |
| 2011 | NHCC-081 | Bangladesh | O1 Ogawa | + | :TLC: :RS1:_:CTX: | - | *ctxB7* | CTX-1 | RS1-ET\|CTX-4 | RS1-ET | CTX-1 |
| 2011 | NHCC-083 | Bangladesh | O1 Ogawa | + | :TLC: :RS1:_:CTX: | - | *ctxB7* | CTX-1 | CTX-1 | RS1-ET | CTX-1 |
| 2011 | NHCM-02 | Bangladesh | O1 Ogawa | + | :TLC: :RS1:_:CTX: | - | *ctxB1* | CTX-1 | CTX-1\|RS1-ET | RS1-ET | CTX-1 |
| 2011 | NHCM-06 | Bangladesh | O1 Ogawa | + | :TLC: :RS1:_:CTX: | - | *ctxB1* | CTX-1 | CTX-4\|RS1-ET | RS1-ET | CTX-1 |
| 2011 | NHCM-04 | Bangladesh | O1 Ogawa | + | :TLC: :RS1:_:CTX: | - | *ctxB1* | CTX-1 | CTX-4\|RS1-ET | RS1-ET | CTX-1 |
| 2011 | NHCM-012 | Bangladesh | O1 Ogawa | + | :TLC: :RS1:_:CTX: | - | *ctxB1* | CTX-1 | CTX-4\|RS1-ET | RS1-ET | CTX-1 |
| 2011 | NHCM-013 | Bangladesh | O1 Ogawa | + | :TLC: :RS1:_:CTX: | - | *ctxB1* | CTX-1 | CTX-1\|RS1-ET | RS1-ET | CTX-1 |
| 2011 | NHCM-016A | Bangladesh | O1 Ogawa | + | :TLC: :RS1:_:CTX: | - | *ctxB1* | CTX-1 | CTX-4\|RS1-ET | RS1-ET | CTX-1 |
| 2011 | NHCM-017 | Bangladesh | O1 Ogawa | + | :TLC: ctxB rstR_rstA rstA rstC | - | *ctxB9* | - | - | RS1-ET | CTX-1 |
| 2011 | NHCM-029 | Bangladesh | NA | + | :TLC: | :CTX:_:RS1: | *ctxB1* | CTX-1 | CTX-4\|RS1-ET | RS1-ET | CTX-1 |
| 2011 | NHCM-044 | Bangladesh | O1 Ogawa | + | :TLC: :RS1:_:CTX: | - | *ctxB7* | CTX-1 | CTX-4\|RS1-ET | RS1-ET | CTX-1 |
| 2011 | NHCM-037 | Bangladesh | O1 Ogawa | + | :TLC: :RS1:_:CTX: | - | *ctxB1* | CTX-1 | RS1-ET\|CTX-1 | RS1-ET | CTX-1 |
| 2011 | NHCM-033 | Bangladesh | O1 Ogawa | + | :TLC: :RS1:_:CTX: | - | *ctxB1* | CTX-1 | CTX-4\|RS1-ET | RS1-ET | CTX-1 |
| 2011 | NHCM-043 | Bangladesh | O1 Ogawa | + | :TLC: :RS1:_:CTX: | - | *ctxB7* | CTX-1 | RS1-ET\|CTX-1 | RS1-ET | CTX-1 |
| 2011 | NHCM-045 | Bangladesh | O1 Ogawa | + | :TLC: :RS1:_:CTX: | - | *ctxB7* | CTX-1 | CTX-2\|RS1-ET | RS1-ET | CTX-1 |
| 2011 | NHCM-053 | Bangladesh | O1 Ogawa | + | :TLC: :RS1:_:CTX: | - | *ctxB1* | CTX-1 | CTX-1\|RS1-ET | RS1-ET | CTX-1 |
| 2011 | NHCM-047 | Bangladesh | O1 Ogawa | + | :TLC: :RS1:_:CTX: | - | *ctxB7* | CTX-1 | CTX-4\|RS1-ET | RS1-ET | CTX-1 |
| 2011 | NHCM-054 | Bangladesh | O1 Ogawa | + | :TLC: :RS1:_:CTX: | - | *ctxB7* | CTX-1 | CTX-1\|RS1-ET | RS1-ET | CTX-1 |
| 2011 | NHCM-048 | Bangladesh | O1 Ogawa | + | :TLC: :RS1:_:CTX: | - | *ctxB1* | CTX-1 | CTX-1 | RS1-ET | CTX-1 |
| 2011 | NHCC-079 | Bangladesh | O1 Ogawa | + | :TLC: :RS1:_:CTX: | - | *ctxB7* | CTX-1 | CTX-1 | RS1-ET | CTX-1 |
| 2011 | NHCC-080 | Bangladesh | O1 Ogawa | + | :TLC: :RS1:_:CTX: | - | *ctxB7* | CTX-1 | CTX-1 | RS1-ET | CTX-1 |
| 2011 | NHCM-03 | Bangladesh | O1 Ogawa | + | :TLC: :RS1:_:CTX: | - | *ctxB1* | CTX-1 | CTX-4\|RS1-ET | RS1-ET | CTX-1 |
| 2011 | MZO-2 | Bangladesh | O14 | - | - | - | *-* | - | - | - | - |
| 2011 | HCUF01 | Haiti | O1 Ogawa | + | :TLC:_:RS1: :TLC: :CTX: | - | *ctxB7* | CTX-1 | RS1-ET\|CTX-6 | RS1-ET | CTX-1 |
| 2011 | HE-46 | Haiti | Oc3 | - | - | - | *-* | - | - | - | - |
| 2011 | EC-0027 | Bangladesh | O1 Ogawa | + | :TLC: :ctx: rstA_rstR rstC | - | *ctxB7* | CTX-1 | - | RS1-ET | CTX-1 |
| 2011 | EC-0051 | Bangladesh | O1 Ogawa | + | :TLC: :TLC: :ctx: rstB_rstA_rstR rstC | - | *ctxB3* | CTX-1 | RS1-ET | CTX-1 | CTX-1 |
| 2011 | EM-1676A | Bangladesh | Oc8 | - | - | - | *-* | - | - | - | - |
| 2011 | 31 | Ukraine | O1 Ogawa | + | :TLC: :ctx: rstC rstB_rstA_rstR | - | *ctxB7* | CTX-1 | RS1-ET | RS1-ET | CTX-1 |
| 2011 | 39 | Ukraine | NA | + | :TLC: :ctx: rstC rstB_rstA_rstR | - | *ctxB7* | CTX-1 | RS1-ET | RS1-ET | CTX-1 |
| 2011 | M1501 | Russia | O1 Ogawa | + | :TLC: rstC | - | *-* | - | - | - | - |
| 2011 | CMR021 | Cameroon | NA | + | :TLC: :CTX: rstA_rstR_rstC | - | *ctxB7* | CTX-1 | CTX-1 | RS1-ET | CTX-1 |
| 2011 | CMR022 | Cameroon | NA | + | :TLC: rstR_rstA rstB_rstC rstB_:ctx: | - | *ctxB7* | CTX-1 | RS1-ET\|CTX-1 | RS1-ET | CTX-1 |
| 2011 | CMR011 | Cameroon | NA | + | :TLC: ctxA_zot_ace_cep_rstB_rstA rstC | - | *-* | CTX-1 | CTX-5 | RS1-ET | - |
| 2011 | CMR012 | Cameroon | NA | + | :TLC: rstA_rstR rstC | :ctx: | *ctxB7* | CTX-1 | - | RS1-ET | CTX-1 |
| 2011 | CMR013 | Cameroon | NA | + | :TLC: :CTX: rstC_rstB | - | *ctxB7* | CTX-1 | CTX-1\|RS1-ET | RS1-ET | CTX-1 |
| 2011 | CMR014 | Cameroon | NA | + | :TLC: ctxA_ctxB rstA cep_orfU_ace_zot rstC_rstB rstR | - | *ctxB7* | CTX-1 | RS1-ET | RS1-ET | CTX-1 |
| 2011 | CMR015 | Cameroon | NA | + | :TLC: rstB_rstA_rstR rstC ctxA_ctxB ace_orfU_cep | - | *ctxB7* | CTX-1 | RS1-ET | RS1-ET | CTX-1 |
| 2011 | CMR016 | Cameroon | NA | + | :TLC: ctxA_ctxB rstC rstR_rstA_rstB zot_ace_orfU_cep | - | *ctxB7* | CTX-1 | CTX-5 | RS1-ET | CTX-1 |
| 2011 | CMR017 | Cameroon | NA | + | :TLC: :ctx: rstR_rstA_rstB rstC | - | *ctxB7* | CTX-1 | RS1-ET | RS1-ET | CTX-1 |
| 2011 | 76 | Ukraine | NA | + | :TLC: :ctx: rstA_rstR rstC | - | *ctxB7* | CTX-1 | - | RS1-ET | CTX-1 |
| 2011 | TEM/12/12-001 | Tanzania | O1 Ogawa | + | :TLC: cep rstC cep zot_ace cep rstR cep | - | *-* | CTX-1 | - | - | CTX-1 |
| 2011 | 39 | Ukraine | NA | + | :TLC: :ctx: rstC rstB_rstA_rstR | - | *ctxB7* | CTX-1 | RS1-ET | RS1-ET | CTX-1 |
| 2011 | 153 | Ukraine | NA | + | :TLC: :ctx: rstR_rstA rstC | - | *ctxB7* | CTX-1 | - | RS1-ET | CTX-1 |
| 2011 | 186 | Ukraine | O1 Ogawa | + | :TLC: :ctx: rstA_rstR | - | *ctxB7* | CTX-1 | - | RS1-ET | CTX-1 |
| 2012 | 2012EL-2176 | Haiti | O1 Ogawa | + | :TLC:_:RS1:_:CTX: | - | *ctxB7* | CTX-1 | CTX-6\|RS1-ET | RS1-ET | CTX-1 |
| 2012 | 2012EL-1759 | Haiti | Oc9 | + | :TLC:_:TLC:_rstR cep_orfU_ace_zot rstC rstB | rstC | *-* | CTX-USGulf | CTX-USGulf | - | CTX-O139 |
| 2012 | 2012Env-2 | Haiti | Od2 | - | - | - | *-* | - | - | - | - |
| 2012 | 2012Env-131 | Haiti | O1 Ogawa | + | :TLC: :ctx: rstC rstR_rstA_rstB | - | *ctxB7* | CTX-1 | RS1-ET | RS1-ET | CTX-1 |
| 2012 | 2012Env-32 | Haiti | Od3 | - | - | - | *-* | - | - | - | - |
| 2012 | 2012Env-326 | Haiti | O1 Ogawa | + | :TLC: rstR_rstA_rstB ctxB_ctxA ace_orfU_cep rstC | - | *ctxB7* | CTX-1 | RS1-ET | RS1-ET | CTX-1 |
| 2012 | 2012Env-90 | Haiti | O1 Ogawa | + | :TLC: rstC rstR_rstA_rstB :ctx: | - | *ctxB7* | CTX-1 | RS1-ET | RS1-ET | CTX-1 |
| 2012 | 2012Env-92 | Haiti | Od1 | - | - | - | *-* | - | - | - | - |
| 2012 | 2012HC-25 | Haiti | Od5 | - | - | - | *-* | - | - | - | - |
| 2012 | 2012HC-24 | Haiti | O1 Ogawa | + | :TLC: :ctx: rstC rstB_rstA_rstR | - | *ctxB7* | CTX-1 | RS1-ET | RS1-ET | CTX-1 |
| 2012 | 2012HC-34 | Haiti | O1 Ogawa | + | :TLC: :ctx: rstC rstB_rstA_rstR | - | *ctxB7* | CTX-1 | RS1-ET | RS1-ET | CTX-1 |
| 2012 | 2012HC-31 | Haiti | O1 Ogawa | + | :TLC: :ctx: rstC rstB_rstA_rstR | - | *ctxB7* | CTX-1 | RS1-ET | RS1-ET | CTX-1 |
| 2012 | 2012Env-94 | Haiti | O1 Ogawa | + | :TLC: :ctx: rstC rstB_rstA_rstR | - | *ctxB7* | CTX-1 | RS1-ET | RS1-ET | CTX-1 |
| 2012 | 2012HC-07 | Haiti | O1 Ogawa | + | :TLC: :ctx: rstC rstB_rstA_rstR | - | *ctxB7* | CTX-1 | RS1-ET | RS1-ET | CTX-1 |
| 2012 | 2012HC-18 | Haiti | O1 Ogawa | + | :TLC: :ctx: rstC rstB_rstA_rstR | - | *ctxB7* | CTX-1 | RS1-ET | RS1-ET | CTX-1 |
| 2012 | 2012HC-17 | Haiti | O1 Ogawa | + | :TLC: :ctx: rstC rstB_rstA_rstR | - | *ctxB7* | CTX-1 | RS1-ET | RS1-ET | CTX-1 |
| 2012 | 2012HC-32 | Haiti | O1 Ogawa | + | :TLC: :ctx: rstC rstB_rstA_rstR | - | *ctxB7* | CTX-1 | RS1-ET | RS1-ET | CTX-1 |
| 2012 | 2012HC-35 | Haiti | O1 Ogawa | + | :TLC: :ctx: rstC rstB_rstA_rstR | - | *ctxB7* | CTX-1 | RS1-ET | RS1-ET | CTX-1 |
| 2012 | 2012HC-33 | Haiti | O1 Ogawa | + | :TLC: :ctx: rstC rstB_rstA_rstR | - | *ctxB7* | CTX-1 | RS1-ET | RS1-ET | CTX-1 |
| 2012 | 2012HC-21 | Haiti | O1 Ogawa | + | :TLC: :ctx: rstC rstB_rstA_rstR | - | *ctxB7* | CTX-1 | RS1-ET | RS1-ET | CTX-1 |
| 2012 | 2012HC-22 | Haiti | O1 Ogawa | + | :TLC: :ctx: rstC rstB_rstA_rstR | - | *ctxB7* | CTX-1 | RS1-ET | RS1-ET | CTX-1 |
| 2012 | 2012HC-11 | Haiti | O1 Ogawa | + | :TLC: :ctx: rstC rstB_rstA_rstR | - | *ctxB7* | CTX-1 | RS1-ET | RS1-ET | CTX-1 |
| 2012 | 2012HC-16 | Haiti | NA | + | :TLC: :ctx: rstC rstB_rstA_rstR | - | *ctxB7* | CTX-1 | RS1-ET | RS1-ET | CTX-1 |
| 2012 | 2012HC-15 | Haiti | O1 Ogawa | + | :TLC: :ctx: rstC rstB_rstA_rstR | - | *ctxB7* | CTX-1 | RS1-ET | RS1-ET | CTX-1 |
| 2012 | 2012HC-19 | Haiti | NA | + | :TLC: :ctx: rstC rstB_rstA_rstR | - | *ctxB7* | CTX-1 | RS1-ET | RS1-ET | CTX-1 |
| 2012 | 2012HC-08 | Haiti | O1 Ogawa | + | :TLC: :ctx: rstC rstB_rstA_rstR | - | *ctxB7* | CTX-1 | RS1-ET | RS1-ET | CTX-1 |
| 2012 | 2012HC-10 | Haiti | O1 Ogawa | + | :TLC: :ctx: rstC rstB_rstA_rstR | - | *ctxB7* | CTX-1 | RS1-ET | RS1-ET | CTX-1 |
| 2012 | 2012HC-12 | Haiti | O1 Ogawa | + | :TLC: :ctx: rstC rstB_rstA_rstR | - | *ctxB7* | CTX-1 | RS1-ET | RS1-ET | CTX-1 |
| 2012 | RND6878 | Russia | O1 Ogawa | + | :TLC: :ctx: rstC rstB_rstA_rstR | - | *ctxB7* | CTX-1 | RS1-ET | RS1-ET | CTX-1 |
| 2012 | M1518 | Russia | O1 Ogawa | + | :TLC: | - | *-* | - | - | - | - |
| 2012 | 2012Env-9 | Haiti | O1 Ogawa | + | - | - | *-* | - | - | - | - |
| 2012 | Env-390 | Haiti | O1 Ogawa | + | - | - | *-* | - | - | - | - |
| 2012 | TEM/29/01-003 | Tanzania | O1 Ogawa | + | :TLC: cep cep cep rstC rstA_rstB cep zot_ace rstR | - | *-* | CTX-1 | CTX-1 | CTX-1 | CTX-1 |
| 2012 | TEM/15/01-005 | Tanzania | O1 Ogawa | + | :TLC: rstR zot_ace cep rstA_rstB cep cep cep rstC | - | *-* | CTX-1 | CTX-1 | RS1-ET | CTX-1 |
| 2012 | TEM/25/01-004 | Tanzania | O1 Ogawa | + | :TLC: rstA_rstB rstC zot_ace cep rstR | - | *-* | CTX-1 | CTX-1 | CTX-1 | CTX-1 |
| 2012 | CISM_1163068.5 | Mozambique | Od4 | - | - | - | *-* | - | - | - | - |
| 2012 | TEM/10/01-002 | Tanzania | O1 Ogawa | + | :TLC: :RS1:_:CTX: | - | *ctxB1* | CTX-1 | CTX-1\|RS1-ET | RS1-ET | CTX-1 |
| 2012 | TEM/04/01-001 | Tanzania | O1 Ogawa | + | :TLC: cep rstC rstR zot_ace rstA_rstB | - | *-* | CTX-1 | CTX-1 | CTX-1 | CTX-1 |
| 2013 | InDRE 4354 | Mexico | O1 Ogawa | + | :TLC: :ctx:_rstB rstC_rstB | - | *ctxB7* | CTX-1 | RS1-ET\|CTX-6 | - | - |
| 2013 | InDRE 4262 | Mexico | O1 Ogawa | + | :TLC: :ctx:_rstB rstC_rstB | - | *ctxB7* | CTX-1 | CTX-6\|RS1-ET | - | - |
| 2013 | InDRE 3140 | Mexico | O1 Ogawa | + | :TLC: :ctx:_rstB_rstR_rstC_rstB | - | *ctxB7* | CTX-1 | CTX-6\|RS1-ET | - | CTX-1 |
| 2013 | E306 | China | O139 | + | :TLC:_:RS1:_:CTX: | - | *ctxB3* | CTX-1 | CTX-2\|RS1-ET | RS1-ET | CTX-1 |
| 2013 | Drakes2013 | United States | Od6 | - | - | - | *-* | - | - | - | - |
| 2013 | W4-13 | India | NA | + | :TLC:_:CTX: | - | *ctxB7* | CTX-1 | CTX-6 | RS1-ET | CTX-1 |
| 2013 | S002502 | Bangladesh | O1 Ogawa | + | :TLC: :RS1:_:CTX: | - | *ctxB1* | CTX-1 | CTX-1 | RS1-ET | CTX-1 |
| 2013 | S002300_E | Bangladesh | O1 Ogawa | + | :TLC: :RS1:_:CTX: | - | *ctxB1* | CTX-1 | CTX-1 | RS1-ET | CTX-1 |
| 2013 | S000600_C10 | Bangladesh | O1 Ogawa | + | :TLC: :RS1:_:CTX: | - | *ctxB1* | CTX-1 | CTX-1 | RS1-ET | CTX-1 |
| 2013 | 22043300_C6 | Bangladesh | O1 Ogawa | + | :TLC: :RS1:_:CTX: | - | *ctxB1* | CTX-1 | CTX-1 | RS1-ET | CTX-1 |
| 2013 | 330033_C1 | Bangladesh | O1 Ogawa | + | :TLC: :RS1:_:CTX: | - | *ctxB1* | CTX-1 | CTX-1 | RS1-ET | CTX-1 |
| 2013 | S000100_C5 | Bangladesh | NA | + | :TLC: :RS1:_:CTX: | - | *ctxB1* | CTX-1 | RS1-ET\|CTX-1 | RS1-ET | CTX-1 |
| 2013 | S081300_C2 | Bangladesh | O1 Ogawa | + | :TLC: :RS1:_:CTX: | - | *ctxB1* | CTX-1 | CTX-1 | RS1-ET | CTX-1 |
| 2013 | 331721_C1 | Bangladesh | NA | + | :TLC: :RS1:_:CTX: | - | *ctxB1* | CTX-1 | CTX-1 | RS1-ET | CTX-1 |
| 2013 | 22043204_C1 | Bangladesh | O1 Ogawa | + | :TLC: :RS1:_:CTX: | - | *ctxB1* | CTX-1 | CTX-1 | RS1-ET | CTX-1 |
| 2013 | 330013_C1 | Bangladesh | NA | + | :TLC: :RS1:_:CTX: | - | *ctxB1* | CTX-1 | CTX-1 | RS1-ET | CTX-1 |
| 2013 | S002506 | Bangladesh | O1 Ogawa | + | :TLC: :RS1:_:CTX: | - | *ctxB1* | CTX-1 | CTX-1 | RS1-ET | CTX-1 |
| 2013 | 330073_A | Bangladesh | O1 Ogawa | + | :TLC: :RS1:_:CTX: | - | *ctxB1* | CTX-1 | CTX-1 | RS1-ET | CTX-1 |
| 2013 | S002300_B | Bangladesh | O1 Ogawa | + | :TLC: :RS1:_:CTX: | - | *ctxB1* | CTX-1 | CTX-1 | RS1-ET | CTX-1 |
| 2013 | 330920_B | Bangladesh | O1 Ogawa | + | :TLC: :RS1:_:CTX: | - | *ctxB1* | CTX-1 | CTX-1 | RS1-ET | CTX-1 |
| 2013 | 330920_A | Bangladesh | O1 Ogawa | + | :TLC: :RS1:_:CTX: | - | *ctxB1* | CTX-1 | CTX-1 | RS1-ET | CTX-1 |
| 2013 | 330073_B | Bangladesh | O1 Ogawa | + | :TLC: :RS1:_:CTX: | - | *ctxB1* | CTX-1 | CTX-1 | RS1-ET | CTX-1 |
| 2013 | 330440_C1 | Bangladesh | O1 Ogawa | + | :TLC: :RS1:_:CTX: | - | *ctxB1* | CTX-1 | CTX-1 | RS1-ET | CTX-1 |
| 2013 | 22087102_C2 | Bangladesh | O1 Ogawa | + | :TLC: :RS1:_:CTX: | - | *ctxB1* | CTX-1 | CTX-1 | RS1-ET | CTX-1 |
| 2013 | 22043202_C1 | Bangladesh | O1 Ogawa | + | :TLC: :RS1:_:CTX: | - | *ctxB1* | CTX-1 | CTX-1 | RS1-ET | CTX-1 |
| 2013 | 330898_C2 | Bangladesh | O1 Ogawa | + | :TLC: :RS1:_:CTX: | - | *ctxB1* | CTX-1 | CTX-1 | RS1-ET | CTX-1 |
| 2013 | 22044108_C3 | Bangladesh | O1 Ogawa | + | :TLC: :RS1:_:CTX: | - | *ctxB1* | CTX-1 | CTX-1 | RS1-ET | CTX-1 |
| 2013 | S040602_C1 | Bangladesh | O1 Ogawa | + | :TLC: :RS1:_:CTX: | - | *ctxB1* | CTX-1 | CTX-1 | RS1-ET | CTX-1 |
| 2013 | 22087500_C9 | Bangladesh | O1 Ogawa | + | :TLC: :RS1:_:CTX: | - | *ctxB1* | CTX-1 | CTX-1 | RS1-ET | CTX-1 |
| 2013 | S042100 | Bangladesh | NA | + | :TLC: :RS1:_:CTX: | - | *ctxB1* | CTX-1 | CTX-1 | RS1-ET | CTX-1 |
| 2013 | 22043200_C1 | Bangladesh | O1 Ogawa | + | :TLC: :RS1:_:CTX: | - | *ctxB1* | CTX-1 | CTX-1 | RS1-ET | CTX-1 |
| 2013 | 4295STDY6534232 | Bangladesh | O139 | + | :TLC:_:TLC:_:TLC:_:RS1:_:CTX:_:CTX:_rstR_rstA_rstB_cep_orfU_ace_zot | - | *ctxB4\|ctxB5* | CTX-1 | CTX-1\|CTX-2 | CTX-1\|RS1-cla | CTX-1\|CTX-O139 |
| 2014 | RND81 | Russia | NA | + | :TLC: ctxA_ctxB rstC cep_orfU_ace_zot rstR_rstA | - | *ctxB1* | CTX-1 | - | RS1-ET | CTX-1 |
| 2014 | 3265/80 | Russia | O1 Ogawa | + | :TLC: rstC :ctx: rstR_rstA | - | *ctxB7* | CTX-1 | - | RS1-ET | CTX-1 |
| 2014 | 81 | Russia | O1 Inaba | + | :TLC: rstC :ctx: rstR_rstA | - | *ctxB1* | CTX-1 | - | RS1-ET | CTX-1 |
| 2014 | FORC_055 | South Korea | Od7 | - | - | - | *-* | - | - | - | - |
| 2014 | 330590 | Bangladesh | O1 Ogawa | + | :TLC: :RS1:_:CTX: | - | *ctxB1* | CTX-1 | RS1-ET\|CTX-1 | RS1-ET | CTX-1 |
| 2014 | S003806 | Bangladesh | O1 Ogawa | + | :TLC: :RS1:_:CTX: | - | *ctxB1* | CTX-1 | CTX-1 | RS1-ET | CTX-1 |
| 2014 | S042408 | Bangladesh | O1 Ogawa | + | :TLC: :RS1:_:CTX: | - | *ctxB1* | CTX-1 | CTX-1 | RS1-ET | CTX-1 |
| 2014 | 330113 | Bangladesh | O1 Ogawa | + | :TLC: :RS1:_:CTX: | - | *ctxB1* | CTX-1 | CTX-1 | RS1-ET | CTX-1 |
| 2014 | 220076-6 | Bangladesh | O1 Ogawa | + | :TLC: :RS1:_:CTX: | - | *ctxB1* | CTX-1 | CTX-1 | RS1-ET | CTX-1 |
| 2014 | 220075-6 | Bangladesh | O1 Ogawa | + | :TLC: :RS1:_:CTX: | - | *ctxB1* | CTX-1 | CTX-1 | RS1-ET | CTX-1 |
| 2014 | 330110 | Bangladesh | O1 Ogawa | + | :TLC: :RS1:_:CTX: | - | *ctxB1* | CTX-1 | CTX-1 | RS1-ET | CTX-1 |
| 2014 | S023208 | Bangladesh | O1 Ogawa | + | - | - | *-* | - | - | - | - |
| 2014 | S003202 | Bangladesh | O1 Ogawa | + | :TLC: :RS1:_:CTX: | - | *ctxB1* | CTX-1 | CTX-1 | RS1-ET | CTX-1 |
| 2014 | 330081 | Bangladesh | O1 Ogawa | + | :TLC: :RS1:_:CTX: | - | *ctxB1* | CTX-1 | CTX-1 | RS1-ET | CTX-1 |
| 2014 | S023202 | Bangladesh | O1 Ogawa | + | :TLC: :RS1:_:CTX: | - | *ctxB1* | CTX-1 | RS1-ET\|CTX-1 | RS1-ET | CTX-1 |
| 2014 | S002604 | Bangladesh | O1 Ogawa | + | :TLC: :RS1:_:CTX: | - | *ctxB1* | CTX-1 | CTX-1 | RS1-ET | CTX-1 |
| 2014 | S003008 | Bangladesh | O1 Ogawa | + | :TLC: :RS1:_:CTX: | - | *ctxB1* | CTX-1 | CTX-1 | RS1-ET | CTX-1 |
| 2014 | HC1037 | Haiti | O1 Ogawa | + | :TLC:_:RS1:_:CTX: | - | *ctxB7* | CTX-1 | CTX-6\|RS1-ET | RS1-ET | CTX-1 |
| 2014 | 8 | Russia | O1 Ogawa | - | - | - | *-* | - | - | - | - |
| 2014 | 4295STDY6534216 | Bangladesh | O139 | + | :TLC:_:TLC:_:TLC:_:RS1:_:CTX:_:CTX:_rstR_rstA_rstB_cep_orfU_ace_zot | - | *ctxB4\|ctxB5* | CTX-1 | CTX-1\|CTX-2 | CTX-1\|RS1-cla | CTX-1\|CTX-O139 |
| 2014 | 4295STDY6534248 | Bangladesh | O139 | + | :TLC:_:TLC:_:TLC:_:RS1:_:CTX:_:CTX:_rstR_rstA_rstB_cep_orfU_ace_zot | - | *ctxB4\|ctxB5* | CTX-1 | CTX-1\|CTX-2 | CTX-1\|RS1-cla | CTX-1\|CTX-O139 |
| 2014 | UG026 | Uganda | O1 Ogawa | + | :TLC: :RS1:_:CTX: | - | *ctxB7* | CTX-1 | CTX-1\|RS1-ET | RS1-ET | CTX-1 |
| 2014 | UG071 | Uganda | O1 Inaba | + | :TLC: :RS1:_:CTX: | - | *ctxB1* | CTX-1 | CTX-1\|RS1-ET | RS1-ET | CTX-1 |
| 2014 | UG060 | Uganda | O1 Inaba | + | :TLC: :RS1:_:CTX: | - | *ctxB1* | CTX-1 | CTX-1\|RS1-ET | RS1-ET | CTX-1 |
| 2015 | 17609 | Tanzania | O1 Ogawa | + | :TLC: :CTX:_:RS1: | - | *ctxB1* | CTX-1 | CTX-1\|RS1-ET | RS1-ET | CTX-1 |
| 2015 | 20390 | Tanzania | O1 Ogawa | + | :TLC: :CTX:_:RS1: | - | *ctxB1* | CTX-1 | CTX-1\|RS1-ET | RS1-ET | CTX-1 |
| 2015 | 39361 | Tanzania | O1 Ogawa | + | :TLC: :CTX:_:RS1: | - | *ctxB7* | CTX-1 | CTX-1\|RS1-ET | RS1-ET | CTX-1 |
| 2015 | 43Ki | Tanzania | O1 Inaba | + | :TLC: :CTX:_:RS1: | - | *ctxB1* | CTX-1 | CTX-1\|RS1-ET | RS1-ET | CTX-1 |
| 2015 | J8YRS KAGUNGA | Tanzania | O1 Ogawa | + | :TLC: :CTX:_:RS1: | - | *ctxB1* | CTX-1 | CTX-1\|RS1-ET | RS1-ET | CTX-1 |
| 2015 | 19886 | Tanzania | O1 Ogawa | + | :TLC: :CTX:_:RS1: | - | *ctxB1* | CTX-1 | CTX-1\|RS1-ET | RS1-ET | CTX-1 |
| 2015 | 21027 | Tanzania | O1 Ogawa | + | :TLC: :CTX:_:RS1: | - | *ctxB1* | CTX-1 | CTX-1\|RS1-ET | RS1-ET | CTX-1 |
| 2015 | 36KI | Tanzania | O1 Inaba | + | :TLC: rstB_rstC_:CTX: | - | *ctxB1* | CTX-1 | CTX-1\|RS1-ET | RS1-ET | CTX-1 |
| 2015 | O1S | Tanzania | O1 Ogawa | + | :TLC: :CTX:_:RS1: | - | *ctxB7* | CTX-1 | CTX-1\|RS1-ET | RS1-ET | CTX-1 |
| 2015 | O7S | Tanzania | O1 Ogawa | + | :TLC: :CTX:_:RS1: | - | *ctxB7* | CTX-1 | CTX-1\|RS1-ET | RS1-ET | CTX-1 |
| 2015 | O9S | Tanzania | O1 Ogawa | + | :TLC: :CTX:_:RS1: | - | *ctxB7* | CTX-1 | CTX-1\|RS1-ET | RS1-ET | CTX-1 |
| 2015 | O2 | Tanzania | O1 Inaba | + | :TLC: :CTX:_:RS1: | - | *ctxB1* | CTX-1 | CTX-1\|RS1-ET | RS1-ET | CTX-1 |
| 2015 | OO4 | Tanzania | O1 Ogawa | + | :TLC: :CTX:_:RS1: | - | *ctxB7* | CTX-1 | CTX-1\|RS1-ET | RS1-ET | CTX-1 |
| 2015 | 47623 | Tanzania | O1 Ogawa | + | :TLC: :CTX:_:RS1: | - | *ctxB7* | CTX-1 | CTX-1\|RS1-ET | RS1-ET | CTX-1 |
| 2015 | 47610 | Tanzania | O1 Ogawa | + | :TLC: :CTX:_:RS1: | - | *ctxB7* | CTX-1 | CTX-1\|RS1-ET | RS1-ET | CTX-1 |
| 2015 | 8Mo | Tanzania | O1 Ogawa | + | :TLC: :CTX:_:RS1: | - | *ctxB7* | CTX-1 | CTX-1\|RS1-ET | RS1-ET | CTX-1 |
| 2015 | 11S | Tanzania | O1 Ogawa | + | :TLC: :RS1:_:CTX: | - | *ctxB7* | CTX-1 | CTX-1\|RS1-ET | RS1-ET | CTX-1 |
| 2015 | O6MU | Tanzania | O1 Ogawa | + | :TLC: :RS1:_:CTX: | - | *ctxB7* | CTX-1 | CTX-1\|RS1-ET | RS1-ET | CTX-1 |
| 2015 | 48055 | Tanzania | O1 Ogawa | + | :TLC: :RS1:_:CTX: | - | *ctxB7* | CTX-1 | CTX-1\|RS1-ET | RS1-ET | CTX-1 |
| 2015 | 7714 | Tanzania | O1 Ogawa | + | :TLC: :RS1:_:CTX: | - | *ctxB7* | CTX-1 | CTX-1\|RS1-ET | RS1-ET | CTX-1 |
| 2015 | 1Mo | Tanzania | O1 Ogawa | + | :TLC: :RS1:_:CTX: | - | *ctxB7* | CTX-1 | CTX-1\|RS1-ET | RS1-ET | CTX-1 |
| 2015 | O3MU | Tanzania | O1 Ogawa | + | :TLC: :RS1:_:CTX: | - | *ctxB7* | CTX-1 | CTX-1\|RS1-ET | RS1-ET | CTX-1 |
| 2015 | 39Ki | Tanzania | O1 Inaba | + | :TLC: :RS1:_:CTX: | - | *ctxB1* | CTX-1 | CTX-1\|RS1-ET | RS1-ET | CTX-1 |
| 2015 | 31Ki | Tanzania | O1 Inaba | + | :TLC: :RS1:_:CTX: | - | *ctxB1* | CTX-1 | CTX-1\|RS1-ET | RS1-ET | CTX-1 |
| 2015 | O7MU | Tanzania | O1 Ogawa | + | :TLC: :RS1:_:CTX: | - | *ctxB7* | CTX-1 | CTX-1\|RS1-ET | RS1-ET | CTX-1 |
| 2015 | O3S | Tanzania | O1 Ogawa | + | :TLC: :RS1:_:CTX: | - | *ctxB7* | CTX-1 | CTX-1\|RS1-ET | RS1-ET | CTX-1 |
| 2015 | O5MU | Tanzania | O1 Ogawa | + | :TLC: :RS1:_:CTX: | - | *ctxB7* | CTX-1 | CTX-1\|RS1-ET | RS1-ET | CTX-1 |
| 2015 | 7Mo | Tanzania | O1 Ogawa | + | :TLC: :RS1:_:CTX: | - | *ctxB7* | CTX-1 | CTX-1\|RS1-ET | RS1-ET | CTX-1 |
| 2015 | 9Mo | Tanzania | O1 Ogawa | + | :TLC: :RS1:_:CTX: | - | *ctxB7* | CTX-1 | CTX-1\|RS1-ET | RS1-ET | CTX-1 |
| 2015 | 5Mo | Tanzania | O1 Ogawa | + | :TLC: :RS1:_:CTX: | - | *ctxB7* | CTX-1 | CTX-1\|RS1-ET | RS1-ET | CTX-1 |
| 2015 | 2Mo | Tanzania | O1 Ogawa | + | :TLC: :RS1:_:CTX: | - | *ctxB7* | CTX-1 | CTX-1\|RS1-ET | RS1-ET | CTX-1 |
| 2015 | 21B | Tanzania | O1 Inaba | + | :TLC: :CTX:_:RS1: | - | *ctxB1* | CTX-1 | CTX-1\|RS1-ET | RS1-ET | CTX-1 |
| 2015 | 20478 | Tanzania | O1 Ogawa | + | :TLC: :CTX:_:RS1: | - | *ctxB1* | CTX-1 | CTX-1\|RS1-ET | RS1-ET | CTX-1 |
| 2015 | 2688 | Russia | O1 Ogawa | + | :TLC: | - | *-* | - | - | - | - |
| 2015 | 2613 | Russia | O1 Ogawa | + | :TLC: | - | *-* | - | - | - | - |
| 2015 | 124 | Russia | O1 Ogawa | + | :TLC: | - | *-* | - | - | - | - |
| 2015 | UG054 | Uganda | O1 Inaba | + | :TLC: rstB_rstA_rstR rstC :ctx: | - | *ctxB1* | CTX-1 | RS1-ET | RS1-ET | CTX-1 |
| 2015 | UG042 | Uganda | O1 Inaba | + | :TLC: ctxB_ctxA rstC rstA ace_orfU_cep rstR rstR | - | *ctxB1* | CTX-USGulf | - | CTX-1 | CTX-1\|CTX-cla |
| 2015 | UG046 | Uganda | O1 Inaba | + | :TLC: :ctx: rstC rstB_rstA_rstR | - | *ctxB1* | CTX-1 | CTX-4 | RS1-ET | CTX-1 |
| 2015 | UG086 | Uganda | O1 Inaba | + | :TLC: :RS1:_:CTX: | - | *ctxB1* | CTX-1 | CTX-1\|RS1-ET | RS1-ET | CTX-1 |
| 2015 | UG040 | Uganda | O1 Inaba | + | :TLC: :RS1:_:CTX: | - | *ctxB1* | CTX-1 | CTX-1\|RS1-ET | RS1-ET | CTX-1 |
| 2015 | 2015V-1076 | United States | Oa1 | - | - | - | *-* | - | - | - | - |
| 2016 | 147 | Ukraine | NA | + | :TLC: rstR rstC | :ctx: rstR | *ctxB1* | CTX-1 | - | - | CTX-1\|CTX-cla |
| 2016 | 89 | Ukraine | O1 Ogawa | + | :TLC: rstR rstB_:ctx: rstC_rstB | rstR | *ctxB1* | CTX-1 | CTX-1\|RS1-ET | - | CTX-1\|CTX-cla |
| 2016 | 28 | Ukraine | O1 Ogawa | + | :TLC: rstR rstR rstC rstB :ctx: | - | *ctxB1* | CTX-1 | RS1-ET | - | CTX-1\|CTX-cla |
| 2016 | 114 | Ukraine | O1 Ogawa | - | - | - | *-* | - | - | - | - |
| 2016 | BC1071 | Germany | NA | - | - | - | *-* | - | - | - | - |
| 2016 | UG020 | Uganda | O1 Ogawa | + | :TLC:_:RS1:_:CTX: | - | *ctxB7* | CTX-1 | CTX-1\|RS1-ET | RS1-ET | CTX-1 |
| 2016 | UG010 | Uganda | O1 Ogawa | + | - | - | *-* | - | - | - | - |
| 2016 | 2016V-1062 | United States | Oa1 | - | - | - | *-* | - | - | - | - |
| 2016 | 2016V-1018 | United States | Oa1 | - | - | - | *-* | - | - | - | - |
| 2018 | 09_113 | Brazil | NA | - | - | - | *-* | - | - | - | - |
| ND | HC-40A1 | Haiti | NA | + | :TLC: rstB_rstA_rstR rstC ctxA zot_ace_orfU_cep | - | *-* | CTX-1 | RS1-ET | RS1-ET | CTX-1 |
| ND | HC-48A1 | Haiti | O1 Ogawa | + | :TLC: rstC ctxB_ctxA zot_ace_orfU_cep rstB_rstA_rstR | - | *ctxB7* | CTX-1 | RS1-ET | RS1-ET | CTX-1 |
| ND | HC-70A1 | Haiti | O1 Ogawa | + | :TLC: :ctx: rstC rstB_rstA_rstR | - | *ctxB7* | CTX-1 | RS1-ET | RS1-ET | CTX-1 |
| ND | HFU-02 | Haiti | O1 Ogawa | + | :TLC: rstC ctxB_ctxA_zot_orfU_cep rstB_rstA_rstR | - | *ctxB7* | CTX-1 | RS1-ET | RS1-ET | CTX-1 |
| ND | VL426 | United Kingdom | Od8 | - | - | - | *-* | - | - | - | - |
| ND | AM-19226 | ND | O39 | - | - | - | *-* | - | - | - | - |
| ND | 623-39 | ND | Oa5 | - | - | - | *-* | - | - | - | - |
| ND | V52 | Sudan | O37 | + | :TLC:_rstR_rstA_rstB_cep_orfU_ace_zot_ctxA_rstC ctxB_ctxA_zot ctxB_cep_orfU | rstC | *ctxB9\|ctxB8* | CTX-USGulf | CTX-1 | CTX-cla | CTX-1 |
| ND | MZO-3 | ND | O37 | - | - | - | *-* | - | - | - | - |
| ND | HE39 | ND | Oc3 | - | - | - | *-* | - | - | - | - |
| ND | HC-61A1 | ND | O1 Ogawa | + | :TLC:_RS1:_:CTX: | - | *ctxB7* | CTX-1 | CTX-6\|RS1-ET | RS1-ET | CTX-1 |
| ND | PCS-022 | ND | O1 Ogawa | + | :TLC: :RS1: :CTX: | - | *ctxB1* | CTX-1 | - | RS1-ET | CTX-1 |
| ND | KW3 | ND | O1 Ogawa | + | :TLC:_:TLC:_:RS1:_:CTX: | - | *ctxB7* | CTX-1 | CTX-6\|RS1-ET | RS1-ET | CTX-1 |
| ND | CMR020 | Cameroon | NA | + | zot_orfU_cep :TLC: rstC rstA | - | *-* | CTX-1 | - | RS1-ET | - |
| ND | CMR019 | Cameroon | NA | + | :TLC: :CTX: rstC_rstR_rstA_rstB | - | *ctxB7* | CTX-1 | CTX-1\|RS1-ET | RS1-ET | CTX-1 |
| ND | CMR018 | Cameroon | NA | + | :TLC: rstR_rstA_cep_orfU_ace rstC | ctxA_ctxB | *ctxB7* | CTX-1 | - | RS1-ET | CTX-1 |
| ND | CISM_S/Nida | Mozambique | O1 Ogawa | + | :TLC: :RS1:_:CTX: | - | *ctxB1* | CTX-1 | CTX-1 | RS1-ET | CTX-1 |
| ND | CISM_780298.0 | Mozambique | O1 Ogawa | + | :TLC: :RS1:_:CTX: | - | *ctxB1* | CTX-1 | CTX-1 | RS1-ET | CTX-1 |
| ND | CISM_770180.8 | Mozambique | O1 Ogawa | + | :TLC: :RS1:_:CTX: | - | *ctxB1* | CTX-1 | CTX-1 | RS1-ET | CTX-1 |
| ND | CISM_770067.4 | Mozambique | O1 Ogawa | + | :TLC: :RS1:_:CTX: | - | *ctxB1* | CTX-1 | CTX-1 | RS1-ET | CTX-1 |
| ND | CISM_710180.8 | Mozambique | O1 Ogawa | + | :TLC: :RS1:_:CTX: | - | *ctxB1* | CTX-1 | CTX-1 | RS1-ET | CTX-1 |
| ND | CISM_740115.4 | Mozambique | O1 Ogawa | + | :TLC: :RS1:_:CTX: | - | *ctxB1* | CTX-1 | CTX-4\|RS1-ET | RS1-ET | CTX-1 |
| ND | CISM_655665.0 | Mozambique | O1 Ogawa | + | :TLC: :RS1:_:CTX: | - | *ctxB1* | CTX-1 | CTX-1 | RS1-ET | CTX-1 |
| ND | CISM_769845.7 | Mozambique | O1 Ogawa | + | :TLC: :RS1:_:CTX: | - | *ctxB1* | CTX-1 | CTX-1 | RS1-ET | CTX-1 |
| ND | CISM_655630.3 | Mozambique | O1 Ogawa | + | :TLC: :RS1:_:CTX: | - | *ctxB1* | CTX-1 | CTX-4\|RS1-ET | RS1-ET | CTX-1 |
| ND | CISM_511 | Mozambique | O1 Ogawa | + | :TLC: :RS1:_:CTX: | - | *ctxB1* | CTX-1 | CTX-1 | RS1-ET | CTX-1 |
| ND | CISM_655664.3 | Mozambique | O1 Ogawa | + | :TLC: :RS1:_:CTX: | - | *ctxB1* | CTX-1 | CTX-1 | RS1-ET | CTX-1 |
| ND | CISM_510 | Mozambique | O1 Ogawa | + | :TLC: :RS1:_:CTX: | - | *ctxB1* | CTX-1 | CTX-4\|RS1-ET | RS1-ET | CTX-1 |
| ND | CISM_420 | Mozambique | O1 Ogawa | + | :TLC: :RS1:_:CTX: | - | *ctxB1* | CTX-1 | CTX-1 | RS1-ET | CTX-1 |
| ND | CISM_505 | Mozambique | O1 Ogawa | + | :TLC: :RS1:_:CTX: | - | *ctxB1* | CTX-1 | CTX-4\|RS1-ET | RS1-ET | CTX-1 |
| ND | CISM_399 | Mozambique | O1 Ogawa | + | :TLC: :RS1:_:CTX: | - | *ctxB1* | CTX-1 | CTX-4\|RS1-ET | RS1-ET | CTX-1 |
| ND | CISM_398 | Mozambique | NA | + | :TLC: :RS1:_:CTX: | - | *ctxB1* | CTX-1 | CTX-4\|RS1-ET | RS1-ET | CTX-1 |
| ND | CISM_382 | Mozambique | O1 Ogawa | + | :TLC: :RS1:_:CTX: | - | *ctxB1* | CTX-1 | CTX-1 | RS1-ET | CTX-1 |
| ND | CISM_375 | Mozambique | O1 Ogawa | + | :TLC: :RS1:_:CTX: | - | *ctxB1* | CTX-1 | CTX-1 | RS1-ET | CTX-1 |
| ND | CISM_374 | Mozambique | O1 Ogawa | + | :TLC: :RS1:_:CTX: | - | *ctxB1* | CTX-1 | CTX-4\|RS1-ET | RS1-ET | CTX-1 |
| ND | CISM_347 | Mozambique | O1 Ogawa | + | :TLC: :RS1:_:CTX: | - | *ctxB1* | CTX-1 | CTX-1 | RS1-ET | CTX-1 |
| ND | CISM_326 | Mozambique | O1 Ogawa | + | :TLC: :RS1:_:CTX: | - | *ctxB1* | CTX-1 | CTX-1 | RS1-ET | CTX-1 |
| ND | CISM_296 | Mozambique | O1 Ogawa | + | :TLC: :RS1:_:CTX: | - | *ctxB1* | CTX-1 | CTX-1 | RS1-ET | CTX-1 |
| ND | CISM_091 | Mozambique | O1 Ogawa | + | :TLC: :RS1:_:CTX: | - | *ctxB1* | CTX-1 | CTX-4\|RS1-ET | RS1-ET | CTX-1 |
| ND | 155 | Ukraine | O1 Ogawa | + | :TLC: rstR :ctx: rstC rstR | - | *ctxB1* | CTX-1 | - | - | CTX-1\|CTX-cla |
| ND | 85 | Ukraine | O1 Ogawa | + | :TLC: | - | *-* | - | - | - | - |
| ND | M1344 | Russia | O1 Ogawa | + | :TLC: :ctx: rstC rstR_rstA | - | *ctxB1* | CTX-1 | - | RS1-ET | CTX-1 |
| ND | M1030 | Turkmenistan | O1 Inaba | + | :TLC: :ctx: rstB_rstA_rstR rstC | - | *ctxB3* | CTX-1 | RS1-ET | RS1-ET | CTX-1 |
| ND | M1337 | Russia | O1 Ogawa | - | - | - | *-* | - | - | - | - |
| ND | FDAARGOS_103 | Germany | Od8 | - | - | - | *-* | - | - | - | - |
| ND | ATCC 11629 | ND | O1 Ogawa | + | :TLC:_:TLC:_:TLC: :CTX:_rstB_rstA_rstR_:CTX:_rstB_rstA_rstR :TLC:_:TLC: | :TLC:_:TLC:_:TLC:_:CTX: | *ctxB1* | CTX-cla | CTX-cla | CTX-cla | CTX-cla |
| ND | FDAARGOS_223 | United States | O1 var Inaba | + | :TLC:_:TLC:_:CTX:_:RS1:_:RS1: | - | *ctxB3* | CTX-1 | CTX-1\|RS1-ET | CTX-1\|RS1-ET | CTX-1 |
