# Supplementary material for: VicPred: A Vibrio cholerae Genotype Prediction Tool
Source: Front Microbiol. 2021 Sep 9;12:691895. doi: 10.3389/fmicb.2021.691895 (PMC8458814; doi:10.3389/fmicb.2021.691895)
Supplement: Supplementary file 10 [file Table_5.docx]

**Supplementary Table 5. Nucleotide polymorphism of *wbeT* genes of O1 serogroup.**

| Serotype | biotype | | Rep.  strain^*^ | Year | Nucleotide polymorphism | | | | | | | | | | | | | | | | | | | | | | | | | | | | |
| --- | --- | --- | --- | --- | --- | --- | --- | --- | --- | --- | --- | --- | --- | --- | --- | --- | --- | --- | --- | --- | --- | --- | --- | --- | --- | --- | --- | --- | --- | --- | --- | --- | --- |
| **Ogawa** | | Classical | M66-2 | 1937 | 45 | 63 | 66-70 | 99 | 137 | 145 | 179 | 193 | 199 | 227 | 284 | 325 | 345 | 349 | 358 | 396-405 | 437 | 514 | 518 | 529 | 530 | 536 | 568 | 582 | 631 | 644 | 728 | 796 | 901 |
|  |  |  |  |  | G | C | TGTAC | T | T | G | G | A | C | A | C | G | T | C | A | TATTAGGATG | T | T | C | C | A | A | C | A | A | G | C | G | T |
|  |  | Classical | M29 | 1942 | · | · | · | · | · | · | · | · | · | · | · | · |  | · | · | · | · | · | · | A | · | · | · | · | · | · | · | · | · |
|  |  | Classical | O395 | 1965 | · | · | · | · | · | · | T | · | · | · | · | · | *del* | CC | · | · | · | · | · | A | · | · | · | · | · | · | · | · | · |
|  |  | ElTor | MG116226 | 1991 | · | · | · | · | · | · | · | · | · | · | · | · |  | · | · | · | G | · | · | · | · | · | · | · | · | · | · | · | · |
|  |  | ElTor | A152 | 1991 | · | · | · | · | · | A | · | · | · | · | · | · |  | · | · | · | · | · | · | · | · | · | · | · | · | · | · | · | · |
|  |  | ElTor | 2010EL-1749 | 2010 | · | · | · | · | G | · | · | · | · | · | · | · |  | · | · | · | · | · | · | · | · | · | · | · | · | · | · | · | · |
|  |  | ElTor | 6/67 | 1967 | · | · | · | · | · | · | · | · | · | · | · | · |  | · | · | · | · | · | · | · | · | · | · | · | · | A | · | · | · |
| **Inaba mut.** | | Classical | A68 | 1949 | · | · | · | · | · | · | T | · | · | · | · | · |  | CC | · | · | · | · | · | A | · | · | · | · | · | · | · | · | · |
|  |  | Classical | A60 | 1958 | · | · | · | · | · | · | T | · | · | · | · | · |  | · | · | *del* | · | · | · | · | · | · | · | · | · | · | · | · | · |
|  |  | Classical | A61 | 1970 | · | · | · | · | · | · | T | · | · | · | · | · |  | · | · | · | · | · | · | A | · | *del* | · | · | · | · | · | · | · |
|  |  | ElTor | FJ147 | 2005 | · | · | · | · | · | · | · | · | · | · | · | · |  | · | · | · | · | C | · | · | · | · | · | · | · | · | · | · | · |
|  |  | ElTor | MJ-1236 | 1994 | · | · | · | TTGATGCT | · | · | · | · | · | · | · | · |  | · | · | · | · | · | · | · | · | · | · | · | · | · | · | · | · |
|  |  | ElTor | 2740-80 | 1980 | · | · | · | · | · | · | · | · | · | · | · | · |  | T | · | · | · | · | · | · | · | · | · | · | · | · | A | · | · |
|  |  | ElTor | 6210 | 2007 | · | · | · | · | · | · | · | T | · | · | · | · |  | · | · | · | · | · | · | · | · | · | · | · | · | · | · | · | · |
|  |  | ElTor | 4113 | 2003 | A | · | · | · | · | · | · | · | · | · | · | · |  | · | · | · | · | · | · | · | · | · | · | · | · | · | · | · | · |
|  |  | ElTor | YN97083 | 1997 | · | · | · | · | · | · | · | · | · | · | · | · |  | · | · | · | · | · | · | · | · | · | T | · | · | · | · | · | · |
|  |  | ElTor | A22 | 1979 | · | · | · | · | · | · | · | · | · | · | · | · |  | · | · | · | C | · | · | · | · | · | · | · | · | · | · | · | · |
|  |  | ElTor | A18 | 1977 | · | · | · | · | · | · | · | · | · | · | · | · |  | · | · | · | · | · | · | · | · | · | · | · | · | · | · | T | · |
|  |  | ElTor | A177 | 1992 | · | · | · | · | · | · | · | · | · | · | · | · |  | · | · | · | · | · | · | · | · | *del* | · | · | · | · | · | · | · |
|  |  | ElTor | 7687 | 2009 | · | · | · | · | · | · | · | · | · | · | · | · |  | · | · | · | · |  | T | · | · | · | · | · | · | · | · | · | · |
|  |  | ElTor | M818 | 1970 | · | · | · | · | · | · | · | · | T | · | · | · |  | · | · | · | · | · | · | · | · | · | · | · | · | · | · | · | · |
|  |  | ElTor | P18899 | 2006 | · | · | · | · | · | · | · | · | · | · | · | · |  | · | · | · | · | C | · | · | · | · | · | · | AA | · | · | · | · |
|  |  | ElTor | 2009V-1096 | 2009 | · | · | · | · | · | · | · | · | · | · | · | · |  | · | · | · | · | · | · | · | · | *del* | · | · | · | · | · | · | C |
|  |  | ElTor | MAK_97 | 1937 | · | · | · | · | · | · | · | · | · | · | · | · |  | · | · | · | · | · | · | · | · | · | · | · | AA | · | · | · | · |
|  |  | ElTor | 12129(1) | 1985 | · | · | · | · | · | · | · | · | · | · | CATGACAC | · |  | · | · | · | · | · | · | · | · | · | · | · | · | · | · | · | · |
|  |  | ElTor | 3569-08 | 2008 | · | · | · | · | · | · | · | · | · | · | · | · |  | T | *del* | · | · | · | · | · | · | *del* |  | · | · | · | A | · | · |
|  |  | ElTor | VC35 | 2004 | · | · | · | · | · | · | · | · | · | · | · | T |  | · | · | · | · | · | · | · | · | · | · | · | · | · | · | · | · |
|  |  | ElTor | M988 | 1972 | · | CA | · | · | · | · | · | · | · | · | · | · |  | · | · | · | · | · | · | · | · | · | · | · | · | · | · | · | · |
|  |  | ElTor | M299 | 1965 | · | · | · | · | · | · | · | · | · | C | *del* | · |  | · | · | · | · | · | · | · | · | · | · | · | · | · | · | · | · |
|  |  | ElTor | 43Ki | 2015 | · | · | *del* | · | · | · | · | · | · | · | · | · |  | · | · | · | · | · | · | · | · | · | · | · | · | · | · | · | · |
| **Inaba truncated** | | ElTor | A325 | 1993 | *transposase insertion p81* | | | · | · | · | · | · | · | · | · | · |  | · | · | · | · | · | · | · | · | · | · | G | · | · | · | · | · |
|  |  |  |  |  | Below SNPs are referred N16961 | | | | | | | | | |  | | | | | | | Below SNPs are referred M66-2 Ogawa | | | | | | | | | | | |
|  |  | ElTor | N16961 | 1975 | 73 | 122 | 130 | 167 | 171 | 172 | 176 | 453 | 468 | 469 |  | | | | | | | 514 | 518 | 529 | 530 | 536 | 568 | 582 | 631 | 644 | 728 | 796 | 901 |
|  |  |  |  |  | A | T | G | T | A | A | T | T | C | A |  |  |  |  |  |  |  | · | · | · | · | · | · | · | · | · | · | · | · |
|  |  | ElTor | IEC224 | 1990 | · | · | · | · | · | · | · | · | T | C |  |  |  |  |  |  |  | · | · | T | C | · | · | · | · | · | · | · | · |
|  |  | ElTor | I-1471 | 2011 | · | · | GGT | · | · | *del* | · | · | · | · |  |  |  |  |  |  |  | · | · | · | · | · | · | · | · | · | · | · | · |
|  |  | ElTor | I-1300 | 1999 | AA | TT | · | TT | G | *del* | *del* | · | · | · |  |  |  |  |  |  |  | · | · | · | · | · | · | · | · | · | · | · | · |

*Rep. strain: Representative strain
